# Identification of potential quality markers of Zishen Yutai pill based on spectrum–effect relationship analysis

**DOI:** 10.3389/fphar.2023.1211304

**Published:** 2023-06-15

**Authors:** Sijia Wu, Qiuling Huang, Feiya Sheng, Lele Zhang, Liang Zou, Lele Yang, Jiliang Cao, Xiufei Pang, Na Ning, Peng Li

**Affiliations:** ^1^ State Key Laboratory of Quality Research in Chinese Medicine, Institute of Chinese Medical Sciences, University of Macau, Macau, China; ^2^ Guangzhou Baiyunshan Zhongyi Pharmaceutical Co., Ltd., Guangzhou, Guangdong, China; ^3^ School of Basic Medical Sciences, Chengdu University, Chengdu, China; ^4^ School of Food and Biological Engineering, Chengdu University, Chengdu, China; ^5^ College of Pharmacy, Shenzhen Technology University, Shenzhen, China

**Keywords:** Zishen Yutai pill, quality markers, offline two-dimensional liquid chromatography-mass spectrometry, *in vitro*, *in vivo*, spectrum–effect relationship, traditional Chinese medicine

## Abstract

**Introduction:** The current quality evaluation of traditional Chinese medicine (TCM) is difficult to attribute to clinical efficacy due to the complexity of TCM. Zishen Yutai pill (ZYP), a well-known traditional Chinese patent medicine, has been widely used to prevent recurrent miscarriage and treat threatened abortion. However, the chemical components of ZYP are unknown, and there is no convincing quality control method applied on ZYP. Although ZYP has been found to promote endometrial receptivity and treat impending abortion, the substantial basis of the therapeutic effects is unclear. The aim of this study was to clarify the quality markers correlated with the potential medicinal activities and provide a theoretical foundation for scientific quality control and product quality improvement of ZYP.

**Methods:** The chemical constituents of ZYP were comprehensively analyzed by offline two-dimensional liquid chromatography-mass spectrometry (2DLC-LTQ-Orbitrap-MS). The efficacy of the 27 ZYP orthogonal groups was investigated using the HTR-8/SVneo oxidative damage model and migration model *in vitro*, as well as the endometrial receptivity disorder mouse model and premature ovarian failure mouse model *in vivo*. Based on the efficacy and mass spectral results, spectrum–effect relationship analysis was used to identify the chemical components with corresponding pharmacological activities.

**Results:** A total of 589 chemical components were found in ZYP, of which 139 were not identified in the literature. The potential quality markers for ZYP were successfully identified through orthogonal design and spectrum–effect relationship analysis. By combining mass spectrum data and pharmacological results of 27 orthogonal groups, 39 substances were identified as potential quality markers.

**Conclusion:** The approaches used in this study will provide a feasible strategy for the discovery of quality markers with bioactivity and further investigation into the quality evaluation of TCM.

## 1 Introduction

Zishen Yutai pill (ZYP) has been shown to play an important role in treating various reproductive disorders in pharmacological investigations and assisted reproductive technology medications (Maharajan et al., 2021), especially in improving endometrial receptivity (Gao et al., 2015; Li et al., 2021; Chen et al., 2022) and treating threatened abortion (Zhang et al., 2019; Zhang et al., 2019), luteal phase deficiency menstrual problem (Shi et al., 2013; Zheng et al., 2015; Chen et al., 2016), and premature ovarian failure (Xie and Chen, 2017; Yang et al., 2020). There are fifteen Chinese herbal medicines contained in ZYP, including *Cuscuta chinensis* Lam. (CC; WFO accession number: 0001296656), *Panax ginseng* C.A.Mey. (PG; WFO accession number: 0000263606), *Dipsacus inermis* Wall. (DI; WFO accession number: 0000651030), *Eucommia ulmoides* Oliv. (EU; WFO accession number: 0000681176), *Taxillus chinensis* (DC.) Danser (TC; WFO accession number: 0000413163), *Morinda officinalis* F.C.How (MO; WFO accession number: 0000246041), *Codonopsis pilosula* Nannf. (CP; WFO accession number: 0000830580), *Atractylodes macrocephala* Koidz. (AM; WFO accession number: 0000046429), and Asini Corii Colla (ACC; skin gelatin of *Equus asinus* L. donkey). The complicated compound composition of ZYP and the unknown components of Chinese medicine itself have made quality control challenging. For multi-component systems, comprehensive two-dimensional liquid chromatography (2DLC) is an effective separation approach (Shang et al., 2021). 2DLC technology has been widely used in the analysis of complex TCM through online and offline modes (Cao et al., 2018; Dong et al., 2021; Wu et al., 2022). However, because the background noise from the online 2DLC approach would drown out many chemical components in ZYP, the chemical components in ZYP could be enriched by using the offline 2DLC method. Offline 2DLC can also solve the common solvent incompatibility problem in online 2DLC analysis, which is more suitable for ZYP analysis.

In terms of methods for determining quality markers, it has gradually evolved from the early belief that the unique chemicals in Chinese medicinal materials are quality markers to combining pharmacological activities to determine quality markers (Yang et al., 2021). The plans are becoming more rational, and the methods used are becoming more sophisticated. The quality evaluation method of ZYP was mainly achieved by using fingerprint with HPSEC-MALL-RID, FT-IR, and HPLC strategies (Cao et al., 2020; Li et al., 2020). Network pharmacology has also been used to investigate the mechanism of ZYP in the treatment of premature ovaries (Konac et al., 2009; Feng et al., 2021; Dang et al., 2022). No one has employed chemometrics and spectrum–effect relationship analysis to investigate its potential quality markers yet. By integrating UHPLC with pharmacological activity *in vitro* and *in vivo*, complemented by the PLS model, we hope to create 27 orthogonal groups based on various ratios of the nine main medicinal materials in ZYP and obtain potential quality markers.

ZYP is commonly used in the treatment of threatened abortion, recurrent abortion, infertility, and other diseases. Both the mouse model of endometrial receptivity disorder (Catalano et al., 2007) and the mouse model of premature ovarian failure (Lai et al., 2015) are proven and reliable models for studying associated disorders. Therefore, in the model of endometrial receptivity disorder, we chose the expression of genes involved in implantation, such as leukemia inhibitory factor (LIF), matrix metalloproteinases (MMPs) and their natural inhibitors (tissue inhibitors of metalloproteinases, TIMPs), and epidermal growth factor (EGF) (Ajdary et al., 2020) to evaluate the influence of ZYP on the model of endometrial receptivity disorder. Then, we chose gamma-interferon (IFN-γ) (Yin et al., 2018) and tumor necrosis factor-α (TNF-α) (Fu et al., 2012) related to ovarian dysfunction for the model of premature ovarian failure to detect their gene expression, as well as the expression levels of luteinizing hormone (LH), follicle-stimulating hormone (FSH), estrogen (E2) (Keremu et al., 2019), and anti-mullerian hormone (AMH) (Massin et al., 2008), which are features of premature ovarian failure. Trophoblast cells are critical in the development of placental structure and normal function. The ability of trophoblast cells to proliferate, fuse, and invade plays an important role in ensuring a healthy pregnancy (Knofler et al., 2019). As a commonly used method for the development and evaluation of drugs, wound healing assay measures cell invasion in scratch repair, such as cell proliferation or invasion under different culture conditions in wound healing. The placenta is hypoxic during the first trimester of pregnancy, and oxygen concentrations in the placenta rapidly increase after embryo–placenta–maternal blood circulation is fully established. When a mother has pregnancy problems or internal or surgical disorders that damage the antioxidant system of the body, active oxygen free radicals created by the cells are dramatically enhanced, causing oxidative stress to worsen and natural abortion to occur (Chiarello et al., 2020). Therefore, using HTR-8/SVneo cells, the king of trophoblast cells, as the *in vitro* model, the pre-protection effect of ZYP on oxidative stress has been explored using the tert-butyl hydroperoxide (t-BHP)-induced oxidative damage model. In addition, the regulatory effect of ZYP on migration ability of HTR-8/SVneo cells has been investigated using the wound healing model. Finally, based on the mass spectrometry data and the pharmacological results, spectrum–effect relationship analysis was used to identify the potential quality markers of ZYP.

## 2 Materials and methods

### 2.1 Chemicals and reagents

The ZYP product and the powders of nine kinds of medicinal materials, namely, CCL, PGC, DIW, EUO, TCD, MOF, CPN, AMK, and ACC were provided by Guangzhou Baiyunshan Zhongyi Pharmaceutical Co., Ltd (Guangzhou, Guangdong Province, China). HPLC-grade acetonitrile, methanol, and formic acid were purchased from Merck (Darmstadt, Germany). Purified water (18.2 MΩ cm at 25°C) was prepared using a Milli-Q purification system (Millipore, Bedford, MA, United States). RPMI-1640 medium, penicillin–streptomycin, fetal bovine serum (FBS), 0.25% trypsin-EDTA, DMSO, chloroform, isopropanol, ethanol, and reagents used for reverse transcription (RT) were purchased from Thermo Fisher Scientific Inc., and 70% t-BHP was purchased from Macklin Inc. Reactive oxygen species assay kit and DEPC-treated water were bought from Beyotime Biotechnology.

### 2.2 Sample preparation and comprehensive acquisition using offline 2DLC-LTQ-Orbitrap

The extraction method of ZYP product is the same as previously described (Cao et al., 2020). The UHPLC system (Dionex UltiMate 3000 ×2 Dual RSLC, Thermo Fisher Scientific Inc.) was used for two-dimensional liquid sample analysis and preparation, which included the DGP-3600RS dual system pump, WPS-3000TRS autosampler, TCC-3000RS column thermostat, SRD-3600 degasser, RS variable wavelength UV detector, and Chromeleon chromatography management software (SR7.2).

For first-dimensional (1D) LC sample analysis and preparation, an amino column (Waters XBridge BEH Amide column 130 Å, 4.6 × 150 mm, 3.5 μm) served as the first-dimensional preparation column at 40°C column temperature. Following a gradient elution program, a binary mobile phase consisting of 0.1% aqueous formic acid (A) and acetonitrile (B) was utilized at a flow rate of 1.0 mL/min: 0–10 min: 95%–90% (B); 10–20 min: 90% (B); 20–55 min: 90%–60% (B); and 55–60 min: 60% (B). The UV wavelength was set at three channels, 203, 254, and 280 nm, respectively. The 1D LC collected the fractions every 3 min using the automatic partial collector and took 20 consecutive injections of 40 μL injection volume. The fractions were combined separately, concentrated, reconstituted with 200 μL of acetonitrile, and refrigerated at 4°C as the second-dimensional (2D) LC sample.

The 2D LC analysis used the C18 column (Thermo Fisher Hypersil GOLDTM C18, 150 mm × 2.1 mm, 1.9 μm) at 45°C column temperature. The mobile phase was the same as 1D LC, but the flow rate was 0.4 mL/min and the elution gradient differed as follows: 0–5 min, 5% (B); 5–10 min, 5%–20% (B); 10–55 min, 20%–70% (B); 55–60 min, 70%–100% (B); and 60–65 min, 100% (B). The UV wavelength was set at three channels, 203, 254, and 280 nm, respectively.

To analyze the 20 fractions obtained, a Thermo Fisher LTQ-Orbitrap XL mass spectrometer (Thermo Fisher Scientific) equipped with an electrospray ionization (ESI) source was connected to the UPLC system. The ESI source was operated in positive and negative modes. The other MS conditions were set as follows: ion-spray voltage, 4.5 kV/−4.0 kV; capillary temperature, 380/380°C; capillary voltage, 35/−35 V; and tube lens voltage, 100/−100 V in the positive/negative mode. Xcalibur 2.1 software (Thermo Fisher Scientific) was used for data acquisition and processing.

### 2.3 Preparation and metabolomic profiling of the ZYP orthogonal test group

#### 2.3.1 Preparation of ZYP orthogonal test group samples

The nine medicinal materials in ZYP are based on the high-, medium-, and low-quality ratios, and the orthogonal experiment design method of nine factors and three levels is adopted, with the quality ratio adjusted according to Baiyunshan Company’s recommendations. According to the L_27_ (3^9^) orthogonal test method, nine kinds of medicinal materials were combined to form 27 test groups, as indicated in Table 1. Then, the weighed medicinal material powders were mixed and extracted with methanol at a concentration of 0.1 g/mL as described in the previous article (Cao et al., 2020).

**TABLE 1:** L_27_(3^9^) orthogonal test method.

No.	Quality ratio/%								
	CC	PG	DI	EU	TC	MO	CP	AM	ACC
1	8.932	2.977	5.955	11.909	3.573	7.146	14.886	5.955	2.047
2	8.932	2.977	5.955	11.909	10.719	21.438	44.658	17.865	6.141
3	8.932	2.977	5.955	11.909	32.157	64.314	133.974	53.595	18.423
4	8.932	8.931	17.865	35.727	3.573	7.146	14.886	17.865	6.141
5	8.932	8.931	17.865	35.727	10.719	21.438	44.658	53.595	18.423
6	8.932	8.931	17.865	35.727	32.157	64.314	133.974	5.955	2.047
7	8.932	26.793	53.595	107.181	3.573	7.146	14.886	53.595	18.423
8	8.932	26.793	53.595	107.181	10.719	21.438	44.658	5.955	2.047
9	8.932	26.793	53.595	107.181	32.157	64.314	133.974	17.865	6.141
10	26.796	2.977	17.865	107.181	3.573	21.438	133.974	5.955	6.141
11	26.796	2.977	17.865	107.181	10.719	64.314	14.886	17.865	18.423
12	26.796	2.977	17.865	107.181	32.157	7.146	44.658	53.595	2.047
13	26.796	8.931	53.595	11.909	3.573	21.438	133.974	17.865	18.423
14	26.796	8.931	53.595	11.909	10.719	64.314	14.886	53.595	2.047
15	26.796	8.931	53.595	11.909	32.157	7.146	44.658	5.955	6.141
16	26.796	26.793	5.955	35.727	3.573	21.438	133.974	53.595	2.047
17	26.796	26.793	5.955	35.727	10.719	64.314	14.886	5.955	6.141
18	26.796	26.793	5.955	35.727	32.157	7.146	44.658	17.865	18.423
19	80.388	2.977	53.595	35.727	3.573	64.314	44.658	5.955	18.423
20	80.388	2.977	53.595	35.727	10.719	7.146	133.974	17.865	2.047
21	80.388	2.977	53.595	35.727	32.157	21.438	14.886	53.595	6.141
22	80.388	8.931	5.955	107.46	3.573	64.314	44.658	17.865	2.047
23	80.388	8.931	5.955	107.46	10.719	7.146	133.974	53.595	6.141
24	80.388	8.931	5.955	107.46	32.157	21.438	14.886	5.955	18.423
25	80.388	26.793	17.865	11.909	3.573	64.314	44.658	53.595	6.141
26	80.388	26.793	17.865	11.909	10.719	7.146	133.974	5.955	18.423
27	80.388	26.793	17.865	11.909	32.157	21.438	14.886	17.865	2.047

CC, *Cuscuta chinensis* Lam.; PG, *Panax ginseng* C.A.Mey.; DI, *Dipsacus inermis* Wall.; EU, *Eucommia ulmoides* Oliv.; TC, *Taxillus chinensis* (DC.) Danser; MO, *Morinda officinalis* F.C.How; CP, *Codonopsis pilosula* Nannf.; AM, *Atractylodes macrocephala* Koidz.; ACC, Asini Corii Colla.

#### 2.3.2 UHPLC-MS analysis of ZYP orthogonal test group samples

The 27 orthogonal groups were injected with 5 μL using the UHPLC-MS system, which was the same as the system used for 2D LC, but with different mass spectrometry settings. The sole variation is the resolution, which is 30,000 in the 2D LC system and 15,000 in the orthogonal experiment.

### 2.4 Oxidative damage and migration assay of ZYP 27 orthogonal groups on HTR-8/SVneo cells

#### 2.4.1 HTR-8/SVneo cell culture

HTR-8/SVneo cells were obtained from Shanghai Honsun Biological Technology Co., Ltd., with STR cell line authentication. HTR-8/SVneo cells were cultured in RPMI-1640 medium containing 10% FBS and 1% penicillin–streptomycin, and the cells were cultured at 37°C and 5% CO_2_ in a cell incubator. After the cells were grown to adhere to the wall, they were digested with 0.25% trypsin-0.02% EDTA solution and seeded in a 96-well culture plate with a seeding density of 5 × 10^4^/mL, 100 μL/well and grown in an incubator for 24 h until cells adhered to the wall. Afterward, different concentrations of drugs were incubated on the cells after replacing the original medium. After 24 h of incubation, different models were made on the cells, and then the corresponding detection methods were used for detection.

#### 2.4.2 Establishment of the t-BHP oxidative damage model, migration model, and ROS model on HTR-8/SVneo cells

The t-BHP-induced oxidative damage model and migration model were applied to HTR-8/SVneo cells. First, HTR-8/SVneo cells were incubated with 100, 50, 25, and 12.5 μg/mL ZYP for 24 h as pre-protection, and then the t-BHP oxidative damage model was built by incubating with 800 μM t-BHP for 2 h. After incubation with CCK-8 solution for 0.5–1.0 h, the absorbance at 450 nm was measured using a multifunctional enzyme marker (FlexStation 3 Multi-Mode Microplate Reader, Molecular Devices). The survival rate of HTR-8/SVneo cells was calculated using the following formula:

Survival rate (%) = (absorbance value of administration group − absorbance value of blank hole)/(absorbance value of no administration group − absorbance value of blank hole) × 100%

After treatment with 100, 50, 25, and 12.5 μg/mL of ZYP for 24 h as pre-protection for HTR-8/SVneo cells, probe DCFH-DA was diluted at 1:1000 with RPMI-1640 basal medium, the cell culture medium was discarded, and 100 μL/well diluted DCFH-DA working solution was added and incubated for 20 min at 37°C and 5% CO_2_ in a cell incubator. Cells were washed three times with RPMI-1640 basal medium to adequately remove DCFH-DA that did not enter the cells. The administration group and model group were injured by adding 800 μM t-BHP for 2 h, and the same amount of the basal medium was added into the control group. The fluorescence intensity was detected using a microplate reader at the excitation wavelength of 488 nm and the emission wavelength of 525 nm. The fluorescence reduction rate was calculated using the following formula:

Fluorescence reduction rate = (fluorescence value of model group − fluorescence value of administration group)/fluorescence value of model group × 100%

The pre-administration concentration of the migration model is set to 100, 50, 25, and 12.5 μg/mL of ZYP as same as other models. After 24 h of incubation, WoundMaker was used to perform a scratch experiment on the cells. After discarding the orthogonal test group drug solution, 100 μL/well of new medium (without serum) was added, and pictures were taken immediately and 24 h later using Incucyte. The scar width was obtained using Incucyte software, and the scar reduction rate was calculated using the following formula:

Scar reduction rate (%) = scar reduction/scar width at 0 h × 100%

### 2.5 Treatment of ZYP 27 orthogonal groups using the endometrial receptivity disorder mouse model and premature ovarian failure mouse model

#### 2.5.1 Mouse endometrial receptivity disorder model method

All mice studies were approved by the Experimental Animal Ethics Committee of Chengdu University in accordance with the guidelines for the care and use of laboratory animals (approval number: 2002001). A total of 350 female and 150 male SPF-grade Kunming strains of mice, weighing 20 ± 2 g, were purchased from Chengdu Dossy Experimental Animals Co., Ltd. After mice were adaptively raised for 1 week and then screened for estrous cycles by vaginal smear, the mice with normal estrous cycle were randomly divided into 30 groups (9–10 mice per group), including the blank group, model group, whole formula group, and 27 dosing groups with specified ratios. At 9: 00 a.m. daily, the whole prescription group and the dosing group with the specified ratio were administered with the corresponding medicine intragastrically (i.g.). The mice of the control group and the model group were administered with the same volume of saline i.g. for successive 15 days. At 16:00 daily, except for the blank control group, mice in each group were administered with 400 mg/kg hydroxyurea i.g. for 10 consecutive days. On the 11th day, five male rats were added into each group’s cage at 20:00. Then, the vaginal plug was examined at 8: 00 a.m. the next day. The results showed that the day of the vaginal plug was the first day of pregnancy. On the fourth day of pregnancy, the female rats in the blank control group were administered with 8.3 mg/kg mifepristone i.g at 21:00. The mice were euthanized at 9:00 a.m. on the fifth day, and the uteri were dissected, separated, and stored in RNA protection solution and then in the refrigerator at −80°C until further analysis.

#### 2.5.2 RNA extraction, reverse transcription, and RT-qPCR detection in mice

We extracted RNA from the mouse uterine tissue with the QIAGEN kit extraction method. Afterward, we performed RNA reverse transcription using the RevertAid First Strand cDNA Synthesis kit (Thermo Fisher Scientific), and cDNA obtained by reverse transcription was stored in a refrigerator at −80°C. Finally, RT-qPCR was performed using the FastStart Universal SYBR Green Master (Rox) kit (Hoffmann-La Roche Ltd.). Primer sequences used in qPCR detection are listed in Supplementary Table S1.

#### 2.5.3 Mouse premature ovarian failure model

A total of 300 female SPF-grade Kunming strains of mice, weighing 18 ± 2 g, were purchased from Chengdu Dossy Experimental Animals Co., Ltd. The mice were randomly assigned into 30 groups with 9–10 animals in each group after 1 week of adaptive feeding, including a blank group, a model group, a full prescription group, and 27 predetermined dosage groups. Mice in the other groups except the blank group were administered with 90 mg/kg cyclophosphamide intraperitoneally on days 1, 4, and 7 to imitate the animal model of follicular development abnormality caused by the reduced ovarian reserve. For 15 days, the mice in the prescription group and the designated administration group were given corresponding dosage of full prescription or 27 orthogonal samples by gavage every morning at 10:00, while the mice in the control group and the model group were given an equal volume of normal saline by gavage at the same time. On the 15th day, the mice were sedated 2 h after the treatment, the blood was drawn and serum was separated, and the mice’s bilateral ovaries were dissected and separated, preserved in RNA protection solution, and stored at −80°C for subsequent use.

#### 2.5.4 RNA extraction, reverse transcription, and RT-qPCR detection in mice

To extract mouse RNA, we processed the mouse ovarian tissue using the QIAGEN kit extraction technique. Afterward, RNA was reverse-transcribed using the RevertAid First Strand cDNA Synthesis Kit (Thermo Fisher Scientific), and cDNA generated by reverse transcription was stored at −80°C.

### 2.6 Spectrum–effect relationship analysis of 27 ZYP orthogonal groups

Compound Discoverer software was used to extract peak area and retention time from mass spectrometry data, and the analysis procedure was carried out as illustrated in Supplementary Figure S1. The data were divided into four groups based on methanol extraction/water extraction and positive/negative ions and then imported into the software for analysis, with the matching mass spectrometry peak areas, retention time, and MW information predicted by the software. After obtaining mass spectral peak area data, the data were imported into the MetaboAnalyst website and processed through the statistical analysis module to produce standardized peak area data.

After analyzing the mass spectrometry data, they were combined with the data from the single pharmacological model and uploaded into SIMCA software. The peak area was taken as x, and the pharmacological model data including the survival rate, scar reduction rate, fluorescence reduction rate in the HTR-8/SVneo cell model, and mean levels of E2, FSH, LH, AMH, *Tnf-α*, and *Ifn-γ* in the premature ovarian failure mouse model and mean levels of *Mmp9*, *Timp3*, *Lif*, and *Egf* in the endometrial receptivity disorder mouse model were taken as y. The PLS model was used to fit the data and determine the VIP and coefficient values. VIP was greater than one, with a significance level of 0.05. The top 15 were screened and compared using Compound Discoverer software with retention time and MW, and the corresponding compounds were inferred from the MS/MS data.

### 2.7 Data analysis

All data were expressed as the mean standard deviation (mean ± SD). GraphPad Prism 8 software was used for data statistics and graphing, and one-way ANOVA was used to compare the two groups. The statistical significance was defined as *p* < 0.05.

## 3 Results

### 3.1 2DLC-LTQ-Orbitrap MS structure identification

In the creation of a two-dimensional liquid system, the choice of the chromatographic column is critical. To maximize column capacity and improve separation efficiency, the chromatographic column should satisfy orthogonality as much as possible. According to our orthogonal column tests, the amino column was employed in the first-dimension chromatography separation of fractions to increase the retention and separation ability of strong polar compounds, as well as to improve the orthogonality. The classic C18 chromatographic column has a high ability to separate and differentiate weakly polar compounds and is mass spectrometry compatible. As a result, the C18 column was chosen as the second column in this investigation. When comparing the organic phases of acetonitrile and methanol, acetonitrile produces a more stable baseline and detects more mass spectral peaks with better peak shape. In addition, adding 0.1% formic acid to the water phase can improve the ionization intensity, peak shape, and column efficiency. As a result, the acetonitrile–0.1% formic acid water system was chosen for this study.

**FIGURE 1:**
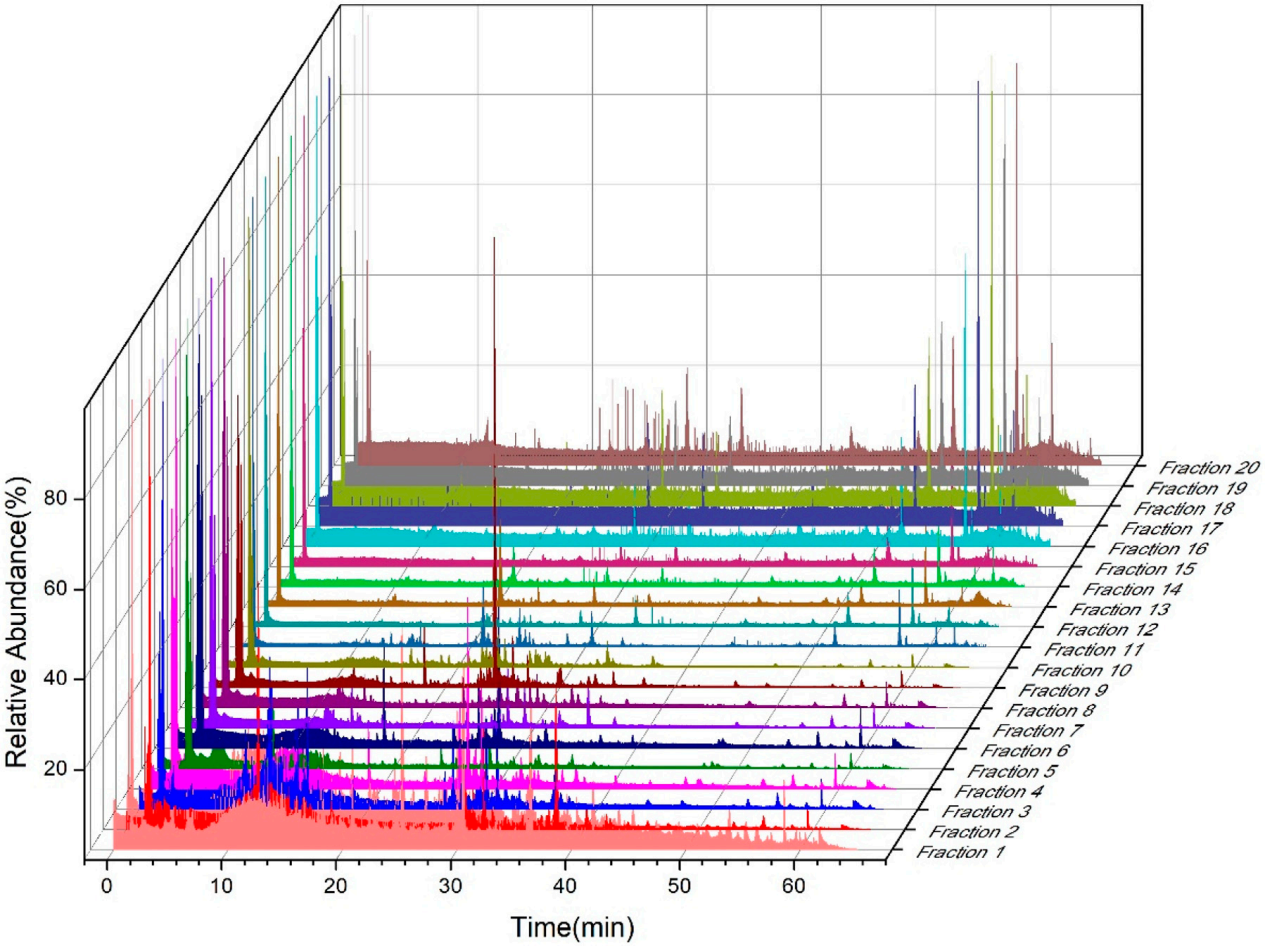
Mass spectra of 20 fractions in the second dimension of offline 2DLC-MS.

According to the separation results of first-dimension liquid chromatography, a fraction was taken every 3 min for the whole retention duration of 60 min, yielding a total of 20 fractions, which were concentrated by nitrogen blowing and freeze-dried. As shown in Figure 1, first-dimension liquid chromatography separated 20 fractions, which were re-dissolved in acetonitrile and evaluated using second-dimension liquid chromatography. Supplementary Tables S2, S3 provide the list of 589 chemicals that can be found in ZYP, of which 221 were found in the positive ion mode of the mass spectrometry and the remaining 368 were found in the negative ion mode. Among them, hyperoside, astragalin, quercetin, kaempferol, isorhamnetin, ginsenoside Rg1, ginsenoside Rb2, ginsenoside Rb3, ginsenoside Rd, ginsenoside F2, ginsenoside Rg2, akebia saponin D, sweroside, chlorogenic acid, 3,5-dicaffeoylquinic acid, 3,4-dicaffeoylquinic acid, 4,5-dicaffeoylquinic acid, pinoresinol di-O-β-D-glucopyranoside, geniposidic acid, nystose, atractylenolide I, atractylenolide II, atractylenolide III, ferulic acid, 5-hydroxymethylfurfural, 2,3,5,4′-tetrahydroxystilbene-2-O-β-Dglucoside, emodin, jaceosidin, eupatilin, and protocatechuic acid were confirmed by standard materials; other compounds were speculated by comparing with the literature report. Furthermore, although 139 compounds found by 2DLC-MS are inconsistent with the data of the reported ingredients, they had the same molecular mass as certain known compounds. This is why the word “isomer” is added when they are reported in Supplementary Tables S2, S3, indicating that they may be new compounds.

In addition to that, the spectra of the 27 orthogonal groups of methanol and water extracts were analyzed using UHPLC-MS, and the positive and negative mode results are shown in Supplementary Table S2, S3. Afterward, *in vitro* and *in vivo* tests were conducted on 27 methanol extracts and 27 water extracts, respectively, and the spectrum–effect connection was then merged.

### 3.2 Pre-protective effect of ZYP orthogonal groups on the oxidative damage and migration model of HTR-8/SVneo cells

**FIGURE 2:**
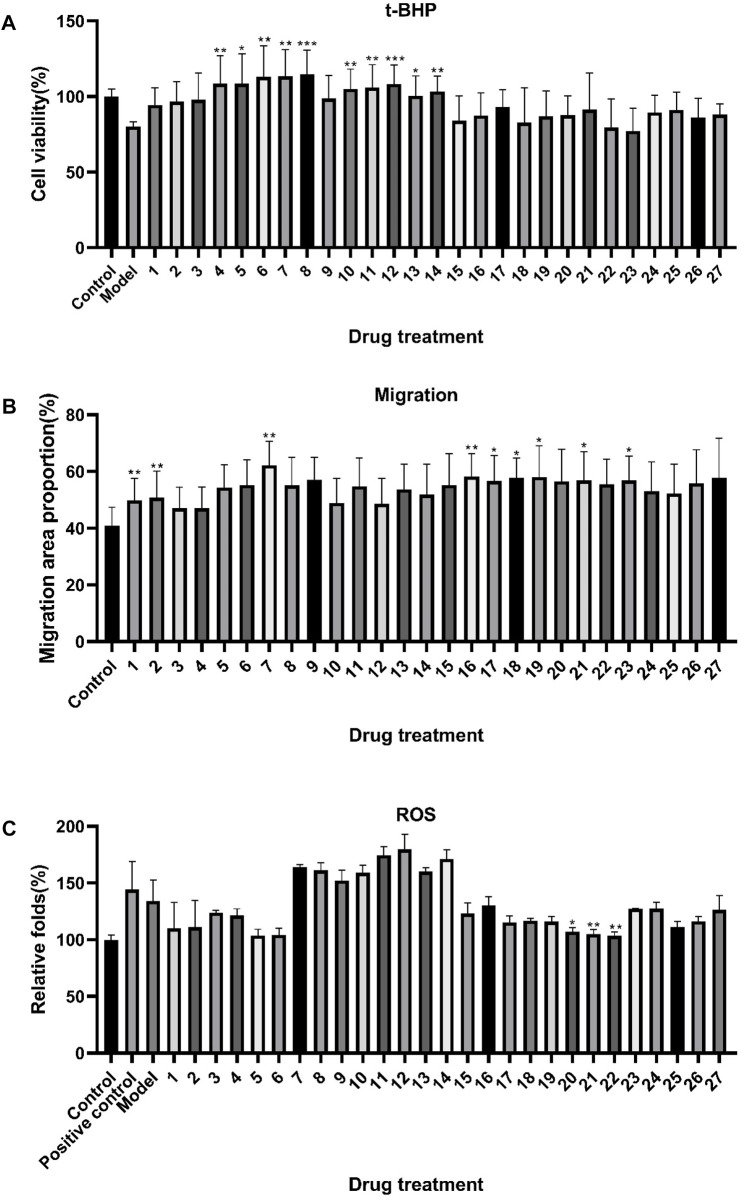
Effects of ZYP orthogonal treatment on the oxidative damage model and migration model of HTR-8/SVneo cells. The results of the **(A)** t-BHP oxidative model, **(B)** migration model, and **(C)** ROS model. The aforementioned values represent the means of three experiments. Data shown represent means ± SD; * *p* < 0.05, ** *p* < 0.01, *** *p* < 0.001 compared to model **(A)**, control **(B)**, and model **(C)** groups.

HTR-8/SVneo cells were incubated with 100, 50, 25, and 12.5 μg/mL ZYP orthogonal groups for 24 h as pre-protection and then treated with t-BHP, WoundMaker, and ROS kit for model building. The results are shown in Supplementary Figures S3, S4, S5. Since the 50 μg/mL concentration had the best overall efficacy among the four concentration groups, the mean value of the 50 μg/mL result was used for the subsequent spectrum–effect relationship analysis. As demonstrated in Figure 2A, t-BHP modeling reduced cell viability by an average of 19.8%, but incubation with 50 μg/mL ZYP orthogonal groups 24 h before modeling increased cell viability to various degrees in each group. The cell survival of the orthogonal test groups 3, 4, 5, 6, 7, 8, 10, 11, 12, 13, and 14 was considerably improved (*p* < 0.05) compared to that of the model group.

In the migration model, all orthogonal groups demonstrated higher HTR-8/SVneo cell wound healing than the control group, as shown in Figure 2B, with groups 1, 2, 7, 16, 17, 18, 19, 21, and 23 being significant (*p* < 0.05). Afterward, the ROS model results shown in Figure 2C indicated that most of the orthogonal groups were able to reduce ROS, except for groups 7–14. In comparison to the model group, the orthogonal groups 20, 21, and 22 showed a considerable effect on reducing ROS (*p* < 0.05).

### 3.3 Effects of ZYP orthogonal groups on the mouse endometrial receptivity disorder model and mouse premature ovarian failure model

**FIGURE 3:**
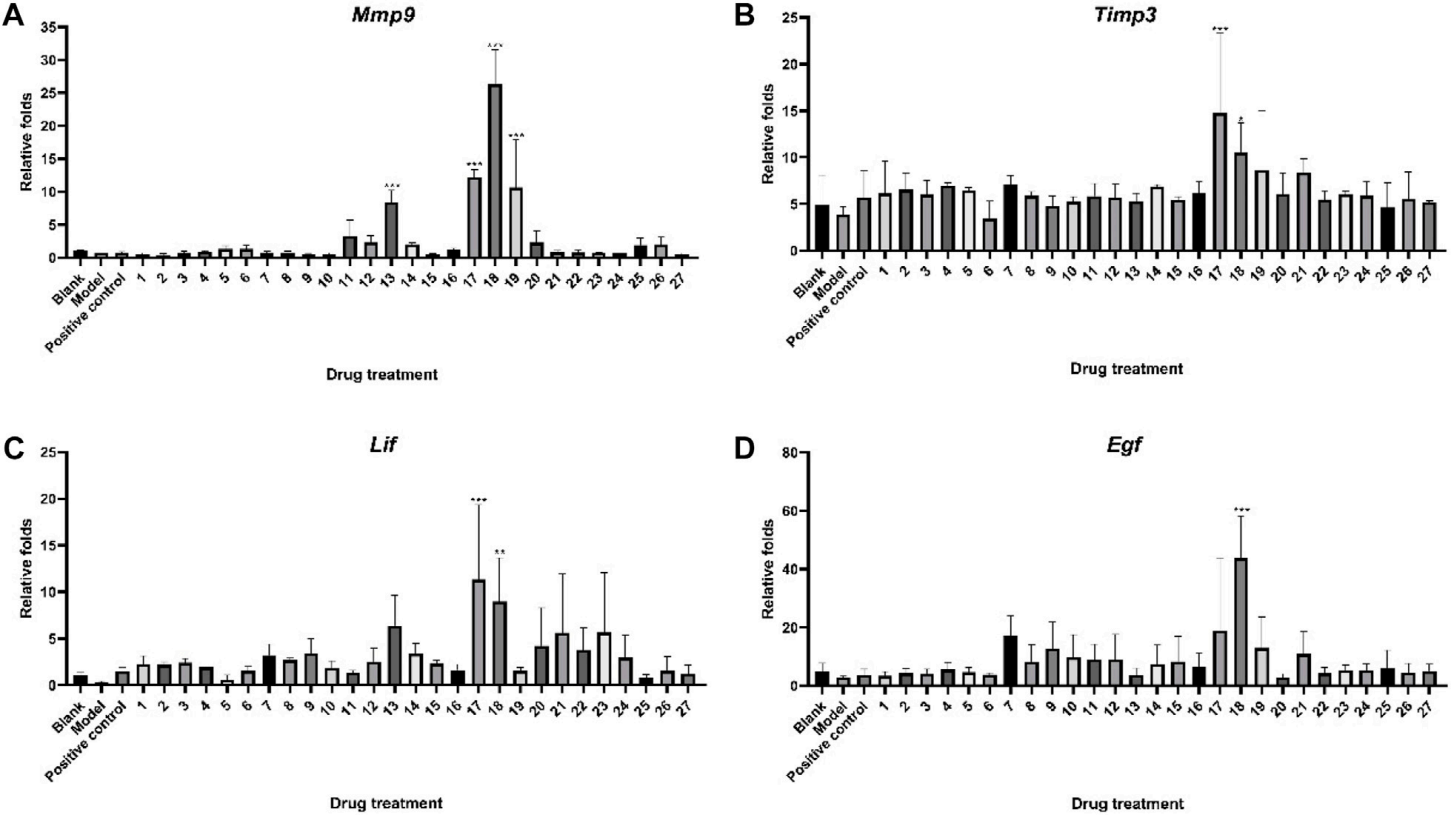
Protective effect of ZYP orthogonal groups on endometrial receptivity disorder in mice. RT-qPCR results of **(A)** *Mmp9*, **(B)** *Timp3*, **(C)** *Lif*, and **(D)** *Egf* in different ZYP orthogonal groups compared with the blank and model groups. The aforementioned values represent the means of three experiments. Data shown represent means ± SD; * *p* < 0.05, ** *p* < 0.01, *** *p* < 0.001 compared to the model group.

Figure 3 depicts the impact of 27 ZYP orthogonal groups on endometrial receptivity disease model mice. *Mmp9*, *Timp3*, *Lif*, and *Egf* mRNA in the mouse uterine tissue could be decreased after modeling. Following administration, some orthogonal groups demonstrated an inhibition of damage caused by modeling medicines, indicating a protective effect. The experimental results showed that groups 17 (*p* < 0.001) and 18 (*p* < 0.01) significantly increased *Lif* mRNA expression in the uterus of endometrial receptivity disorder model mice, implying that the protective action of this part of the administration group may be associated with *Lif*-related targets. The amount of *Egf* mRNA in the uterus of mice in (*p* < 0.001) administration group 18 was likewise considerably greater than that in the model group, implying that administration group 18 (*p* < 0.001) may exercise its medicinal effect via modulating growth factor-related pathways.

**FIGURE 4:**
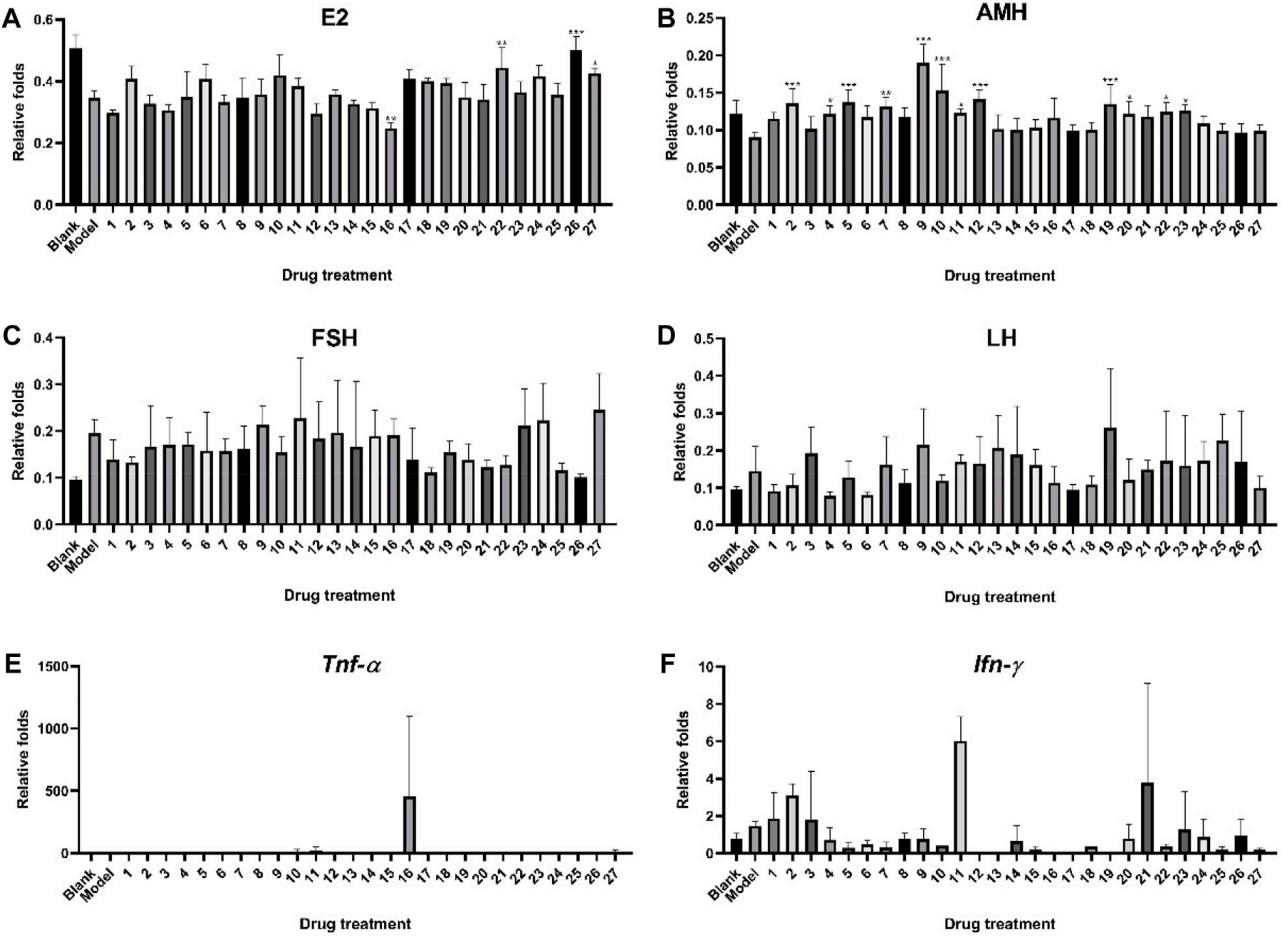
Protective effect of ZYP orthogonal groups on premature ovarian failure in mice. ELISA results of **(A)** E2, **(B)** AMH, **(C)** FSH, and **(D)** LH and RT-qPCR results of **(E)** *Tnf-α* and **(F)** *Ifn-γ* in ZYP orthogonal groups compared with the blank and model groups. The aforementioned values represent the means of three experiments. Data shown represent means ± SD; * *p* < 0.05, ** *p* < 0.01, *** *p* < 0.001 compared to the model group.

Figure 4 illustrates the protecting impact of ZYP 27 orthogonal test groups on a mouse model of premature ovarian failure. The levels of E2 and AMH in the serum of mice were dramatically reduced after continuous modeling treatment with cyclophosphamide, whereas the levels of LH and FSH were significantly increased. As shown in Figure 4A, the serum E2 levels of mice in groups 16, 22 (*p* < 0.01), 26 (*p* < 0.001), and 27 (*p* < 0.05) were considerably greater than those in the model group after treatment, implying that ovarian function is protected by inhibiting E2 reduction. Figure 4B indicates that groups 4, 11, 20, 22, and 23 (*p* < 0.05); 7 (*p* < 0.01); and 2, 5, 9, 10, 12, and 19 (*p* < 0.001) were able to significantly increase cyclophosphamide-caused AMH reduction, implying that these groups may be able to prevent premature ovarian failure by maintaining or increasing AMH release. Cyclophosphamide could considerably raise FSH and LH secretion levels, as shown in Figures 4C, D, which is consistent with the symptoms of premature ovarian failure. Most administration groups exhibited an impact of suppressing excessive FSH and LH secretion after delivery. In the mouse ovarian tissue, cyclophosphamide can generate an increase in the expression levels of *Tnf-α* and *Ifn-γ* mRNA, as demonstrated in Figures 4E, F, and most of the administration groups showed a downward trend. However, because the expression levels of *Tnf-α* and *Ifn-γ* genes in the mouse ovarian tissue were low or not significantly activated, they were below the limit of quantification, or the divergence within the group was too great, and significant differences could not be acquired in the real experimental detection.

### 3.4 Spectrum–effect relationship to obtain potential quality markers

**FIGURE 5:**
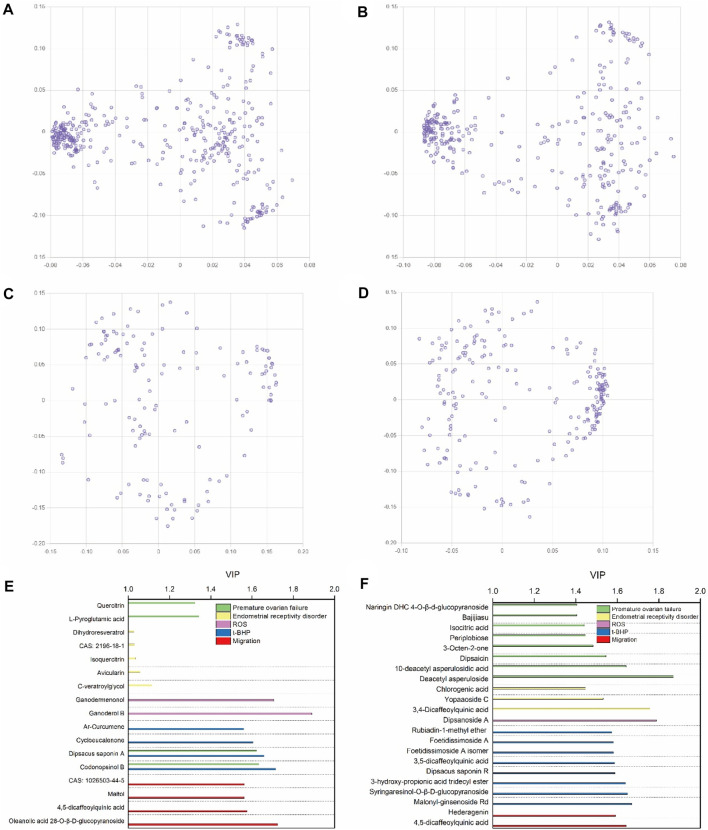
Multivariate data analyses among ZYP orthogonal groups. PCA loading plots for the 27 orthogonal groups’ UHPLC-MS peaks of methanol extracts in positive **(A)** and negative **(B)** modes, and 27 orthogonal groups’ UHPLC-MS peaks of water extracts in positive **(C)** and negative **(D)** modes. The VIP-score plot of 27 orthogonal groups’ UHPLC-MS results in positive **(E)** and negative **(F)** modes combined with pharmacological results.

After the UHPLC-MS and pharmacological experiments of the 27 ZYP orthogonal groups were performed, the quality markers could be inferred by combining the spectrum–effect relationship. Figures 5A, B, C, D show the results of PCA of 27 orthogonal groupings of mass spectrum peaks. The acquired VIP value data are shown in Figures 5E, F when integrated with one single pharmacological model for PLS fitting. A total of 39 potential quality markers were predicted, including 17 in the positive ion mode and 22 in the negative ion mode. Dipsacus saponin A and codonopsinol B are strong predictors in both the HTR-8/SVneo cell model of t-BHP oxidative damage and the premature ovarian failure mouse model.

## 4 Discussion

ZYP is widely used in the treatment of threatened miscarriage, recurrent miscarriage, infertility, and other diseases as a well-known traditional Chinese medicine. The main reason for restricting its industrialization and modernization process is because the pharmacodynamic material basis for its therapeutic effects is unknown, as is the *in vivo* process and method of action. The quality marker proposed by Liu et al. (2016) created a new paradigm for developing the TCM quality control system. It is used to control the manufacturing process and product quality as well as to ensure the clinical efficacy and safety of TCM because of its measurability, specificity, transitivity, effect correlation, and correlation with the theory of TCM (Lu et al., 2022). The strategy of finding quality markers has also made great progress in recent years, from the original chemical consistency evaluation to the consistency evaluation of drug efficacy and safety. As a good example, Liang et al. (2021) evaluated the anti-pancreatic activity *in vitro* and *in vivo* and used linear regression analysis or a weighted least squares model to predict the effective content of chemical markers based on quantitative data between validated batches and other batches of Chaiqin Chengqi decoction. As a result, the lower and upper bounds of the effective and antagonistic constituents were determined. Recently, the quality control of ZYP has been quantitatively assessed by the method of fingerprinting (Cao et al., 2020), but it lacks efficacy evidence to back it up. Therefore, we combined the pharmacological activities *in vivo* and *in vitro* to create a thorough assessment of which components in ZYP have the most impact on the therapeutic effect using the approach of spectrum–effect combination. A good platform for the analysis of ZYP chemical constituents could also be provided by the employment of 2DLC technology, which would also help uncover the chemical ingredients of preparations that are actually active.

Oxidative stress occurs when oxidative and antioxidant components in cells are out of balance, resulting in the formation of more ROS than the removal of ROS, causing cellular and tissue damage. Large levels of ROS build in the placenta under ischemia–hypoxic circumstances, and trophoblast cells are subjected to oxidative stress when pregnancy complications are present (Burton and Jauniaux, 2011). Therefore, reducing or maintaining the level of ROS may be the possible mechanism for ZYP to exert its therapeutic effect. Many molecular markers have been proposed to identify this stage of endometrial receptivity, including integrins, *Egf*, and *Lif* (Aghajanova et al., 2008). ZYP regulates the balance of *Mmp9*/*Timp3* expression, and the mechanism of *Lif*- and *Egf*-related pathways ameliorates endometrial receptivity impairment in mice.

Tables 2, 3 indicate the potential quality markers for investigation after combining the mass spectrum data with the pharmacological model result. We discovered that 39 potential quality markers were screened out using the strategy of combining chemical data with pharmacological models. 4,5-Dicaffeoylquinic acid (Kim et al., 2005), syringaresinol-O-β-D-glucopyranoside (Morgan et al., 2015), 3,5-dicaffeoylquinic acid (Hyun et al., 2019), 3,4-dicaffeoylquinic acid (Hyun et al., 2019), chlorogenic acid (Feng et al., 2016), bajijiasu (Wu et al., 2015), isocitric acid (Morgunov et al., 2018), maltol (Wang et al., 2019), avicularin (Zhang et al., 2020), isoquercitrin (Li et al., 2016), dihydroresveratrol (Torres-Santiago et al., 2021), and quercitrin (Bose et al., 2013) have been reported in pharmacological model experiments related to oxidative damage, while hederagenin (Zeng et al., 2018), rubiadin-1-methyl ether (Mohr et al., 2019), yopaaoside C (Gadicherla et al., 2019), oleanolic acid 28-O-β-D-glucopyranoside (Lin et al., 2016), cycloeucalenone (Lopes et al., 2014), avicularin (Zhang et al., 2020), dihydroresveratrol (Torres-Santiago et al., 2021), and quercitrin (Bose et al., 2013) have been found to be associated with anti-inflammatory mechanisms. Other pharmacological activities reported among the rest of potential quality markers were antimycobacterial activity, cytotoxicity, antiviral, anti-complement effects, and so on. In addition to that, some other compounds have not been reported in related pharmacological activities or their effects need to work in synergy with other compounds, all of which need further verification.

**TABLE 2:** Potential quality markers in ZYP by spectrum–effect relationship (negative ion mode).

Pharmacological model	Identification	Chemical formula	Herb	Pharmacological activity
Migration model	4,5-Dicaffeoylquinic acid	C_25_H_24_O_12_	CC, DI, TC	Inhibits the apoptosis induced by H_2_O_2_
	Hederagenin	C_30_H_48_O_4_	DI	Anti-tumor, anti-inflammatory, anti-depressant, and so on
t-BHP oxidative model	Malonyl-ginsenoside Rd	C_51_H_84_O_21_	PG	Anti-diabetic
	Syringaresinol-O-β-D-glucopyranoside	C_28_H_36_O_13_	TC	Peroxyl radical-scavenging capacities
	3-Hydroxy-propionic acid tridecyl ester	C_16_H_32_O_3_	CP	—
	*Dipsacus* saponin R	C_53_H_86_O_21_	DI	—
	3,5-Dicaffeoylquinic acid	C_25_H_24_O_12_	CC, DI, TC	Inhibits apoptosis induced by H_2_O_2_
	Foetidissimoside A isomer	C_52_H_82_O_22_	—	—
	Foetidissimoside A	C_52_H_82_O_22_	MO	Attenuates amyloid-β-induced memory impairment in mice
	Rubiadin-1-methyl ether	C_16_H_12_O_4_	EU	Anti-inflammatory
ROS model	Dipsanoside A	C_66_H_90_O_37_	DI	—
Endometrial receptivity disorder model	3,4-Dicaffeoylquinic acid	C_25_H_24_O_12_	CC, DI, TC	Scavenged environmental UVB and PM2.5-induced intracellular ROS
	Chlorogenic acid	C_16_H_18_O_9_	CC, DI, TC, MO	*In vivo* and *in vitro* antioxidant
	Yopaaoside C	C_17_H_26_O_12_	EU	Anti-inflammatory
Premature ovarian failure model	Bajijiasu	C_12_H_22_O_11_	EU	*In vivo* and *in vitro* antioxidant
	Deacetyl asperuloside	C_16_H_20_O_10_	EU	Antiviral
	10-Deacetyl asperulosidic acid	C_16_H_20_O_11_	TC, EU	Antiviral
	Dipsaicin	C_14_H_18_O_9_	DI	—
	3-Octen-2-one	C_8_H_14_O	AM	—
	Periplobiose	C_13_H_24_O_9_	TC	—
	Naringin DHC 4-O-β-d-glucopyranoside	C_33_H_44_O_19_	TC	—
	(+)-Pinoresinol di-O-β-D-glucopyranoside	C_32_H_42_O_16_	TC	Anti-complement and PDE inhibitory activities
	Isocitric acid	C_6_H_8_O_7_	TC	Antioxidant

CC, *Cuscuta chinensis* Lam.; PG, *Panax ginseng* C.A.Mey.; DI, *Dipsacus inermis* Wall.; EU, *Eucommia ulmoides* Oliv.; TC, *Taxillus chinensis* (DC.) Danser; MO, *Morinda officinalis* F.C.How; CP, *Codonopsis pilosula* Nannf.; AM, *Atractylodes macrocephala* Koidz.

**TABLE 3:** Potential quality markers in ZYP by spectrum–effect relationship (positive ion mode).

Pharmacological model	Identification	Chemical formula	Herb	Pharmacological activity
Migration model	Oleanolic acid 28-O-β-D-glucopyranoside	C_36_H_58_O_8_	PG	Anti-inflammatory
	4,5-Dicaffeoylquinic acid	C_25_H_24_O_12_	CC, DI, TC	Inhibits the apoptosis induced by H_2_O_2_
	Maltol	C_6_H_6_O_3_	PG	Inhibits the apoptosis induced by H_2_O_2_
	7-[4-(11-Hydroxy-undecyloxy)-phenyl]-7-pyridin-3-yl-hept-6-enoic acid ethyl ester	C_31_H_45_NO_4_	CP	—
t-BHP oxidative model	Codonopsinol B	C_13_H_19_NO_4_	MO	Inhibition of *Bacillus stearothermophilus* alpha-glucosidase
	*Dipsacus* saponin A	C_42_H_68_O_14_	DI	Inhibition of TNF-alpha-induced NF-kappaB activation in human HepG2 cells
	Cycloeucalenone	C_30_H_48_O	TC	Analgesic and anti-inflammatory
	Ar-Curcumene	C_15_H_22_	AM, EU	Anti-mycobacterial activity
ROS model	Ganoderol B	C_30_H_48_O_2_	PG	Anti-androgenic
	Ganodermenonol	C_30_H_46_O_2_	PG	Directs cytotoxicity on Meth-A and LLC tumor cell lines
Endometrial receptivity disorder model	Avicularin	C_20_H_18_O_11_	TC	Reduce inflammation and oxidative stress
	Isoquercitrin	C_21_H_20_O_12_	CC, TC	Antioxidant
	Dihydroresveratrol	C_14_H_14_O_3_	—	Antioxidant, anti-inflammatory, and cytotoxic activity
	C-Veratroylglycol	C_10_H_12_O_5_	TC	—
	3-Hydroxy-1-(4-hydroxy-3-methoxyphenyl) propan-1-one	C_10_H_12_O_4_	CP	Inhibition of COX-2 activity
Premature ovarian failure model	Quercitrin	C_21_H_20_O_11_	CC, AM	Antioxidant and Anti-inflammatory
	Codonopsinol B	C_13_H_19_NO_4_	MO	Inhibition of *Bacillus stearothermophilus* alpha-glucosidase
	*Dipsacus* saponin A	C_42_H_68_O_14_	DI	Inhibition of TNF-alpha-induced NF-kappaB activation in human HepG2 cells
	L-Pyroglutamic acid	C_5_H_7_NO_3_	MO	Inhibits energy production and lipid synthesis

CC, *Cuscuta chinensis* Lam.; PG, *Panax ginseng* C.A.Mey.; DI, *Dipsacus inermis* Wall.; EU, *Eucommia ulmoides* Oliv.; TC, *Taxillus chinensis* (DC.) Danser; MO, *Morinda officinalis* F.C.How; CP, *Codonopsis pilosula* Nannf.; AM, *Atractylodes macrocephala* Koidz.

## 5 Conclusion

In this study, a strategy was established to analyze the chemical compositions of ZYP by offline 2DLC-MS. In addition, potential quality markers were successfully identified through orthogonal design and spectrum–effect relationship analysis.

## Data Availability

The original contributions presented in the study are included in the article/Supplementary Material; further inquiries can be directed to the corresponding author.

## Abbreviations

TCM, traditional Chinese medicine; ZYP, Zishen Yutai pill; 2DLC, two-dimensional liquid chromatography; MS, mass spectrometry; CC, *Cuscuta chinensis* Lam.; PG, *Panax ginseng* C.A.Mey.; DI, *Dipsacus inermis* Wall.; EU, *Eucommia ulmoides* Oliv.; TC, *Taxillus chinensis* (DC.) Danser; MO, *Morinda officinalis* F.C.How; CP, *Codonopsis pilosula* Nannf.; AM, *Atractylodes macrocephala* Koidz.; ACC, Asini Corii Colla; LIF, leukemia inhibitory factor; MMPs, matrix metalloproteinases; TIMPs, tissue inhibitors of metalloproteinases; EGF, epidermal growth factor; IFN-γ, gamma-interferon; TNF-α, tumor necrosis factor-α; LH, luteinizing hormone; FSH, follicle-stimulating hormone; E2, estrogen; AMH, anti-mullerian hormone; t-BHP, tert-butyl hydroperoxide; ROS, reactive oxygen species; ESI, electrospray ionization; 1D, first-dimensional; 2D, second-dimensional.

## References

[B1] Aghajanova L. Hamilton A. E. Giudice L. C. (2008). Uterine receptivity to human embryonic implantation: Histology, biomarkers, and transcriptomics. Semin. Cell Dev. Biol. 19, 204–211. 10.1016/j.semcdb.2007.10.008 18035563 PMC2829661

[B2] Ajdary M. Keyhanfar F. Aflatoonian R. Amani A. Amjadi F. Zandieh Z. (2020). Design and evaluation of a novel nanodrug delivery system for reducing the side effects of clomiphene citrate on endometrium. DARU 28, 423–432. 10.1007/s40199-019-00310-2 32483681 PMC7704853

[B3] Bose S. Maji S. Chakraborty P. (2013). Quercitrin from Ixora coccinea leaves and its anti-oxidant activity. J. Pharm. Sci. Technol. 2, 72–74.

[B4] Burton G. J. Jauniaux E. (2011). Oxidative stress. Best. Pract. Res. Clin. Obstet. Gynaecol. 25, 287–299. 10.1016/j.bpobgyn.2010.10.016 21130690 PMC3101336

[B5] Cao J. L. Lei T. Wu S. J. Li H. Y. Deng Y. Lin R. Z. (2020). Development of a comprehensive method combining UHPLC-CAD fingerprint, multi-components quantitative analysis for quality evaluation of zishen Yutai pills: A step towards quality control of Chinese patent medicine. J. Pharm. Biomed. Anal. 191, 113570. 10.1016/j.jpba.2020.113570 32905860

[B6] Cao J. L. Wang S. S. Hu H. He C. W. Wan J. B. Su H. X. (2018). Online comprehensive two-dimensional hydrophilic interaction chromatography×reversed-phase liquid chromatography coupled with hybrid linear ion trap Orbitrap mass spectrometry for the analysis of phenolic acids in Salvia miltiorrhiza. J. Chromatogr. A 1536, 216–227. 10.1016/j.chroma.2017.09.041 28967384

[B7] Catalano R. D. Critchley H. O. Heikinheimo O. Baird D. T. Hapangama D. Sherwin J. R. A. (2007). Mifepristone induced progesterone withdrawal reveals novel regulatory pathways in human endometrium. Mol. Hum. Reprod. 13, 641–654. 10.1093/molehr/gam021 17584828

[B8] Chen C. R. Yan Q. X. Liu K. P. Zhou X. Q. Xian Y. J. Liang D. L. (2016). Endometrial receptivity markers in mice stimulated with raloxifene versus clomiphene citrate and natural cycles. Reprod. Sci. 23, 748–755. 10.1177/1933719115616496 26603317

[B9] Chen X. Hao C. Deng W. Bai H. Li Y. Wang Z. (2022). Effects of the zishen Yutai pill compared with placebo on live births among women in a fresh embryo transfer cycle: A randomized controlled trial. Obstet. Gynecol. 139, 192–201. 10.1097/AOG.0000000000004658 34991130 PMC8759541

[B10] Chiarello D. I. Abad C. Rojas D. Toledo F. Vazquez C. M. Mate A. (2020). Oxidative stress: Normal pregnancy versus preeclampsia. Biochim. Biophys. Acta-Mol. Basis Dis. 1866, 165354. 10.1016/j.bbadis.2018.12.005 30590104

[B11] Dang L. Zhang C. Su B. Na N. Huang Q. Zhou S. (2022). The genus *apterygothrips* priesner (thysanoptera, phlaeothripinae, haplothripini) from China, with one new species. BMC Complement. Med. Ther.-submitted. 1112, 1–9. 10.3897/zookeys.1112.85902 PMC9848647 36760624

[B12] Dong B. J. Peng C. S. Ma P. Li X. B. (2021). An integrated strategy of MS-network-based offline 2DLC-QTOF-MS/MS coupled with UHPLC-QTRAP^®^-MS/MS for the characterization and quantification of the non-polysaccharides in Sijunzi decoction. Anal. Bioanal. Chem. 413, 3511–3527. 10.1007/s00216-021-03302-x 33851227 PMC8043762

[B13] Feng Y. H. Chai X. Y. Chen Y. Y. Ning Y. Zhao Y. (2021). Network pharmacology approach for predicting targets of zishen Yutai pills on premature ovarian insufficiency. Evid. Based Complement. Altern. Med. 2021, 8215454. 10.1155/2021/8215454 PMC8357500 34394393

[B14] Feng Y. Yu Y. H. Wang S. T. Ren J. Camer D. Hua Y. Z. (2016). Chlorogenic acid protects d-galactose-induced liver and kidney injury via antioxidation and anti-inflammation effects in mice. Pharm. Biol. 54, 1027–1034. 10.3109/13880209.2015.1093510 26810301 PMC11132915

[B15] Fu Y. Zhao Z. G. Wu Y. K. Wu K. M. Xu X. U. Liu Y. (2012). Therapeutic mechanisms of Tongmai Dasheng Tablet on tripterygium glycosides induced rat model for premature ovarian failure. J. Ethnopharmacol. 139, 26–33. 10.1016/j.jep.2011.08.077 22101081

[B16] Gadicherla V. Challa S. R. Rao M. V. B. Kunda P. K. Prudhvi R. (2019). Morinda citrifolia (noni) fruit protects the exocrine pancreatic dysfunction against L-arginine induced acute pancreatitis in rats. Pharmacogn. Mag. 15, 328–334. 10.4103/pm.pm_661_18

[B17] Gao Q. Han L. Li X. Cai X. (2015). Traditional Chinese medicine, the Zishen Yutai pill, ameliorates precocious endometrial maturation induced by controlled ovarian hyperstimulation and improves uterine receptivity via upregulation of HOXA10. Evid. Based Complement. Altern. Med. 2015, 317586. 10.1155/2015/317586 PMC4352469 25792996

[B18] Hyun Y. J. Piao M. J. Kang K. A. Ryu Y. S. Zhen A. X. Cho S. J. (2019). 3,4-Dicaffeoylquinic acid protects human keratinocytes against environmental oxidative damage. J. Funct. Food. 52, 430–441. 10.1016/j.jff.2018.11.026

[B19] Keremu A. Yaoliwasi A. Tuerhong M. Kadeer N. He Y. Yiming A. (2019). Research on the establishment of chronic stress-induced premature ovarian failure the rat model and effects of Chinese medicine Muniziqi treatment. Mol. Reprod. Dev. 86, 175–186. 10.1002/mrd.23092 30512210

[B20] Kim S. S. Park R. Y. Jeon H. J. Kwon Y. S. Chun W. (2005). Neuroprotective effects of 3, 5‐dicaffeoylquinic acid on hydrogen peroxide‐induced cell death in SH‐SY5Y cells. Phytother. Res. 19, 243–245. 10.1002/ptr.1652 15934031

[B21] Knofler M. Haider S. Saleh L. Pollheimer J. Gamage T. James J. (2019). Human placenta and trophoblast development: Key molecular mechanisms and model systems. Cell. Mol. Life Sci. 76, 3479–3496. 10.1007/s00018-019-03104-6 31049600 PMC6697717

[B22] Konac E. Alp E. Onen H. I. Korucuoglu U. Biri A. A. Menevse S. (2009). Endometrial mRNA expression of matrix metalloproteinases, their tissue inhibitors and cell adhesion molecules in unexplained infertility and implantation failure patients. Reprod. Biomed. Online. 19, 391–397. 10.1016/S1472-6483(10)60174-5 19778485

[B23] Lai D. M. Wang F. Y. Yao X. F. Zhang Q. W. Wu X. X. Xiang C. (2015). Human endometrial mesenchymal stem cells restore ovarian function through improving the renewal of germline stem cells in a mouse model of premature ovarian failure. J. Transl. Med. 13, 1–13. 10.1186/s12967-015-0516-y 25964118 PMC4490699

[B24] Li H. Y. Cao J. L. Wu X. Deng Y. Ning N. Geng C. X. (2020). Multiple fingerprint profiling for quality evaluation of polysaccharides and related biological activity analysis of Chinese patent drugs: Zishen Yutai Pills as a case study. J. Ethnopharmacol. 260, 113045. 10.1016/j.jep.2020.113045 32504785

[B25] Li M. Ning N. Liu Y. Li X. Mei Q. Zhou J. (2021). The potential of Zishen Yutai pills to facilitate endometrial recovery and restore fertility after induced abortion in rats. Pharm. Biol. 59, 1505–1516. 10.1080/13880209.2021.1993272 34711116 PMC8555532

[B26] Li X. C. Jiang Q. Wang T. T. Liu J. J. Chen D. F. (2016). Comparison of the antioxidant effects of Quercitrin and Isoquercitrin: Understanding the role of the 6 ''-oh group. Molecules 21, 1246. 10.3390/molecules21091246 27657022 PMC6273918

[B27] Liang G. Yang J. Y. Liu T. T. Wang S. S. Wen Y. J. Han C. X. (2021). A multi-strategy platform for quality control and Q-markers screen of Chaiqin chengqi decoction. Phytomedicine 85, 153525. 10.1016/j.phymed.2021.153525 33740732

[B28] Lin C. Wen X. A. Sun H. B. (2016). Oleanolic acid derivatives for pharmaceutical use: A patent review. Expert Opin. Ther. Pat. 26, 643–655. 10.1080/13543776.2016.1182988 27113324

[B29] Liu C. X. Chen S. L. Xiao X. H. Zhang T. J. Hou W. B. Liao M. L. (2016). A new concept on quality marker of Chinese materia medica: Quality control for Chinese medicinal products. Zhong Cao Yao 22, 1443–1457. 10.7501/j.issn.0253-2670.2016.09.001

[B30] Lopes L. C. de Carvalho J. E. Kakimore M. Vendramini-Costa D. B. Medeiros M. A. Spindola H. M. (2014). Pharmacological characterization of solanum cernuum vell.: 31-norcycloartanones with analgesic and anti-inflammatory properties. Inflammopharmacology 22, 179–185. 10.1007/s10787-013-0182-8 23925459

[B31] Lu X. Y. Jin Y. Y. Wang Y. Z. Chen Y. L. Fan X. H. (2022). Multimodal integrated strategy for the discovery and identification of quality markers in traditional Chinese medicine. J. Pharm. Anal. 12, 701–710. 10.1016/j.jpha.2022.05.001 36320607 PMC9615540

[B32] Maharajan K. Xia Q. Duan X. Y. Tu P. F. Zhang Y. Liu K. C. (2021). Therapeutic importance of zishen Yutai pill on the female reproductive health: A review. J. Ethnopharmacol. 281, 114523. 10.1016/j.jep.2021.114523 34438031

[B33] Massin N. Meduri G. Bachelot A. Misrahi M. Kuttenn F. Touraine P. (2008). Evaluation of different markers of the ovarian reserve in patients presenting with premature ovarian failure. Mol. Cell. Endocrinol. 282, 95–100. 10.1016/j.mce.2007.11.017 18191888

[B34] Mohr E. T. B. Nascimento M. da Rosa J. S. Vieira G. N. Kretzer I. F. Sandjo L. P. (2019). Evidence that the anti-inflammatory effect of rubiadin-1-methyl ether has an immunomodulatory context. Mediat. Inflamm. 2019, 6474168. 10.1155/2019/6474168 PMC6874871 31780865

[B35] Morgan A. Kim J. H. Lee H. W. Lee S.-H. Lim C.-H. Jang H.-D. (2015). Phytochemical constituents from the aerial part of ducrosia ismaelis asch. Nat. Prod. Sci. 21, 6–13. 10.0000/nps.2015.21.1.6

[B36] Morgunov I. G. Karpukhina O. V. Kamzolova S. V. Samoilenko V. A. Inozemtsev A. N. (2018). Investigation of the effect of biologically active threo-Ds-isocitric acid on oxidative stress in Paramecium caudatum. Prep. Biochem. Biotechnol. 48, 1–5. 10.1080/10826068.2017.1381622 28976247

[B37] Shang Z. P. Xu L. L. Xiao Y. Du W. An R. Ye M. (2021). A global profiling strategy using comprehensive two-dimensional liquid chromatography coupled with dual-mass spectrometry platforms: Chemical analysis of a multi-herb Chinese medicine formula as a case study. J. Chromatogr. A 1642, 462021. 10.1016/j.chroma.2021.462021 33714771

[B38] Shi Y. Yang S. Tao L. Zhang Y. (2013). Clinical observation of Zishen Yutai pill in the treatment of diminished ovarian reserve with weak spleen and kidney. J. Shandong Univ. Traditional Chin. Med. 37, 292–294. 10.16294/j.cnki.1007-659x.2013.04.015

[B39] Torres-Santiago G. Zepeda-Vallejo L. G. Serrano-Contreras J. I.(2021). "Linking metabolic profiling, resveratrol, the gut microbiota, and antioxidant potential". in Toxicology. Elsevier, Amsterdam, Netherlands). 317–327.

[B40] Wang Z. Hao W. N. Hu J. N. Mi X. J. Han Y. Ren S. (2019). Maltol improves APAP-induced hepatotoxicity by inhibiting oxidative stress and inflammation response via NF-kappa B and PI3K/akt signal pathways. Antioxidants 8, 395. 10.3390/antiox8090395 31547366 PMC6769439

[B41] Wu R. J. Liang J. Liang Y. H. Xiong L. (2022). A spectrum-effect based method for screening antibacterial constituents in Niuhuang Shangqing Pill using comprehensive two-dimensional liquid chromatography. J. Chromatogr. B 1191, 123121. 10.1016/j.jchromb.2022.123121 35042147

[B42] Wu Z. Q. Chen D. L. Lin F. H. Lin L. Shuai O. Wang J.-Y. (2015). Effect of bajijiasu isolated from Morinda officinalis FC how on sexual function in male mice and its antioxidant protection of human sperm. J. Ethnopharmacol. 164, 283–292. 10.1016/j.jep.2015.02.016 25686781

[B43] Xie J. Chen M. F. (2017). Clinical effect of zishen Yutai pill combined with climen on premature ovarian failure. J. Hunan Univ. Chin. Med. 37, 1396–1399. 10.3969/j.issn.1674-070X.2017.12.023

[B44] Yang J. H. Tang L. H. Wei H. Song X. (2020). Efficacy of kidney nourishing pills combined with estradiol valerate in the treatment of women with premature ovarian failure and its effect on serum estrogens levels. Chongqing Med. 49, 3803–3806. 10.3969/j.issn.1671-8348.2020.22.027

[B45] Yang L. L. Xue Y. Wei J. C. Dai Q. Li P. (2021). Integrating metabolomic data with machine learning approach for discovery of Q-markers from Jinqi Jiangtang preparation against type 2 diabetes. Chin. Med. 16, 30–12. 10.1186/s13020-021-00438-x 33741031 PMC7980607

[B46] Yin N. Zhao W. Luo Q. Q. Yuan W. D. Luan X. Y. Zhang H. Q. (2018). Restoring ovarian function with human placenta-derived mesenchymal stem cells in autoimmune-induced premature ovarian failure mice mediated by treg cells and associated cytokines. Reprod. Sci. 25, 1073–1082. 10.1177/1933719117732156 28954601

[B47] Zeng J. Huang T. Xue M. Chen J. X. Feng L. L. Du R. F. (2018). Current knowledge and development of hederagenin as a promising medicinal agent: A comprehensive review. Rsc Adv. 8, 24188–24202. 10.1039/c8ra03666g 35539158 PMC9082113

[B48] Zhang Y. Yan W. Ge P.-F. Li Y. Ye Q. (2019). Study on prevention effect of Zishen Yutai pill combined with progesterone for threatened abortion in rats. Asian pac. J. Trop. Med. 9, 577–581. 10.1016/j.apjtm.2016.04.002 27262070

[B49] Zhang Z. T. Lv G. D. Du L. (2020). Avicularin reduces the expression of mediators of inflammation and oxidative stress in bradykinin-treated MG-63 human osteoblastic osteosarcoma cells. Med. Sci. Monit. 26, 921957. 10.12659/msm.921957 PMC7278328 32463805

[B50] Zheng Y. X. Zhao Y. Luo S. P. (2015). Effect observation of Zishen Yutai Pill on hypomenorrhea of kidney deficiency type. J. Chin. Med. Mater. 55, 203–205. 10.13863/j.issn1001-4454.2015.01.054

