# Supplementary material for: Identification of potential quality markers of Zishen Yutai pill based on spectrum–effect relationship analysis
Source: Front Pharmacol. 2023 Jun 15;14:1211304. doi: 10.3389/fphar.2023.1211304 (PMC10311498; doi:10.3389/fphar.2023.1211304)
Supplement: Supplementary file 1 [file DataSheet1.docx]

**Identification of potential** **quality markers of Zishen Yutai Pill based on** **spectrum-effect relationship analysis**

Sijia Wu ^a,^ ^†^, Qiuling Huang ^b, †^, Feiya Sheng ^c, †^, Lele Zhang ^c^, Liang Zou ^d,^**^*^**, Lele Yang ^a^, Jiliang Cao ^e^, Xiufei Pang ^b^, Na Ning ^b,^**^*^**, and Peng Li ^a,^**^*^**

^a^ State Key Laboratory of Quality Research in Chinese Medicine, Institute of Chinese Medical Sciences, University of Macau, Macau 999078, China

^b^ Guangzhou Baiyunshan Zhongyi Pharmaceutical Co. Ltd, Guangzhou, Guangdong, China

^c^ School of Basic Medical Sciences, Chengdu University, Chengdu, China

^d^ School of Food and Biological Engineering, Chengdu University, Chengdu, China

^e^ College of Pharmacy, Shenzhen Technology University, Shenzhen, China

^†^ These authors contributed equally to this work.

* Corresponding authors

*E-mail addresses:* [zouliangcdu@126.com](mailto:zouliangcdu@126.com) (L. Zou); [1125835438@qq.com](mailto:1125835438@qq.com) (N. Ning); [pli1978@hotmail.com](mailto:pli1978@hotmail.com) (P. Li)


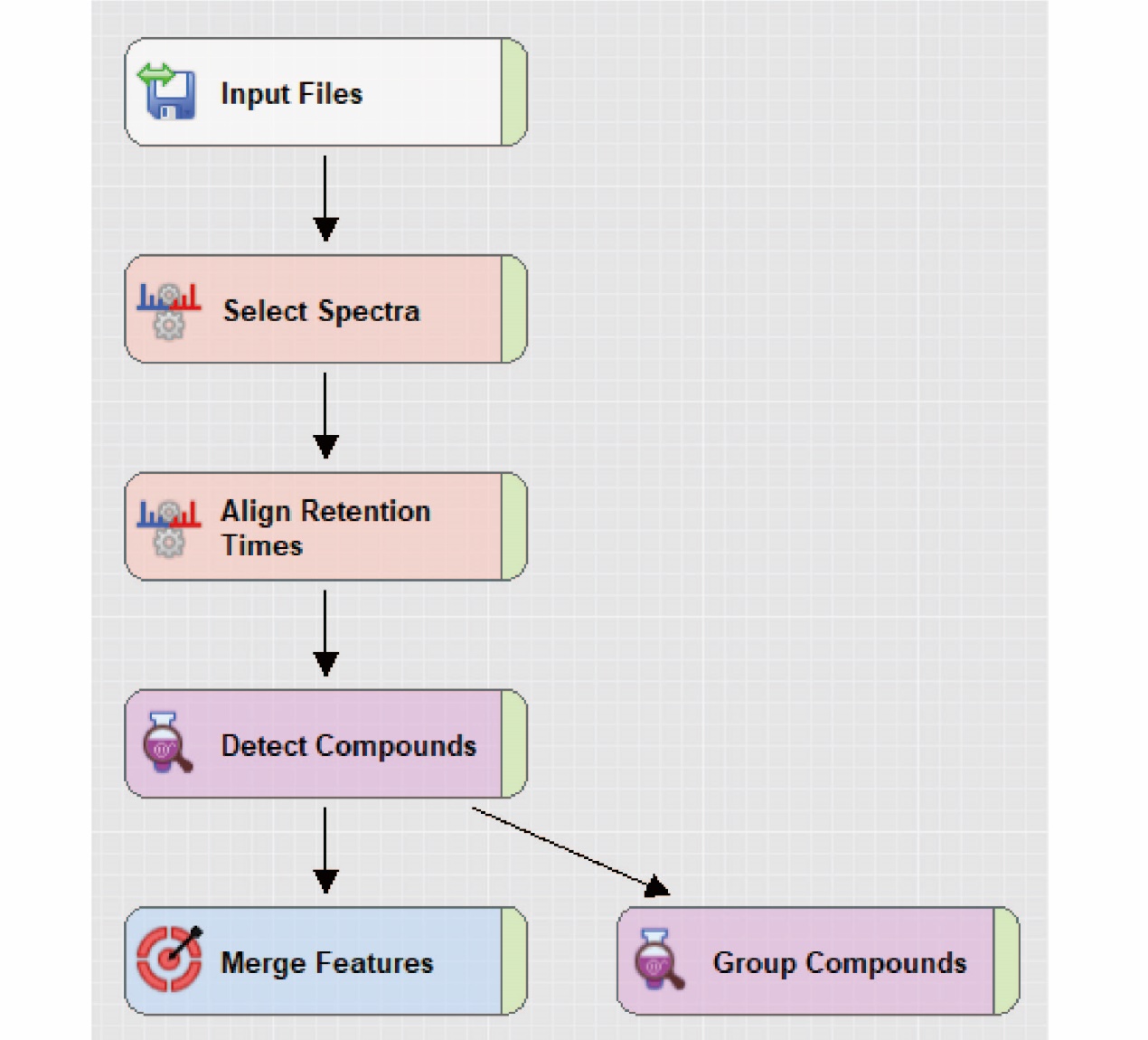


Figure. S1 Compound Discoverer software analysis Workflow Tree


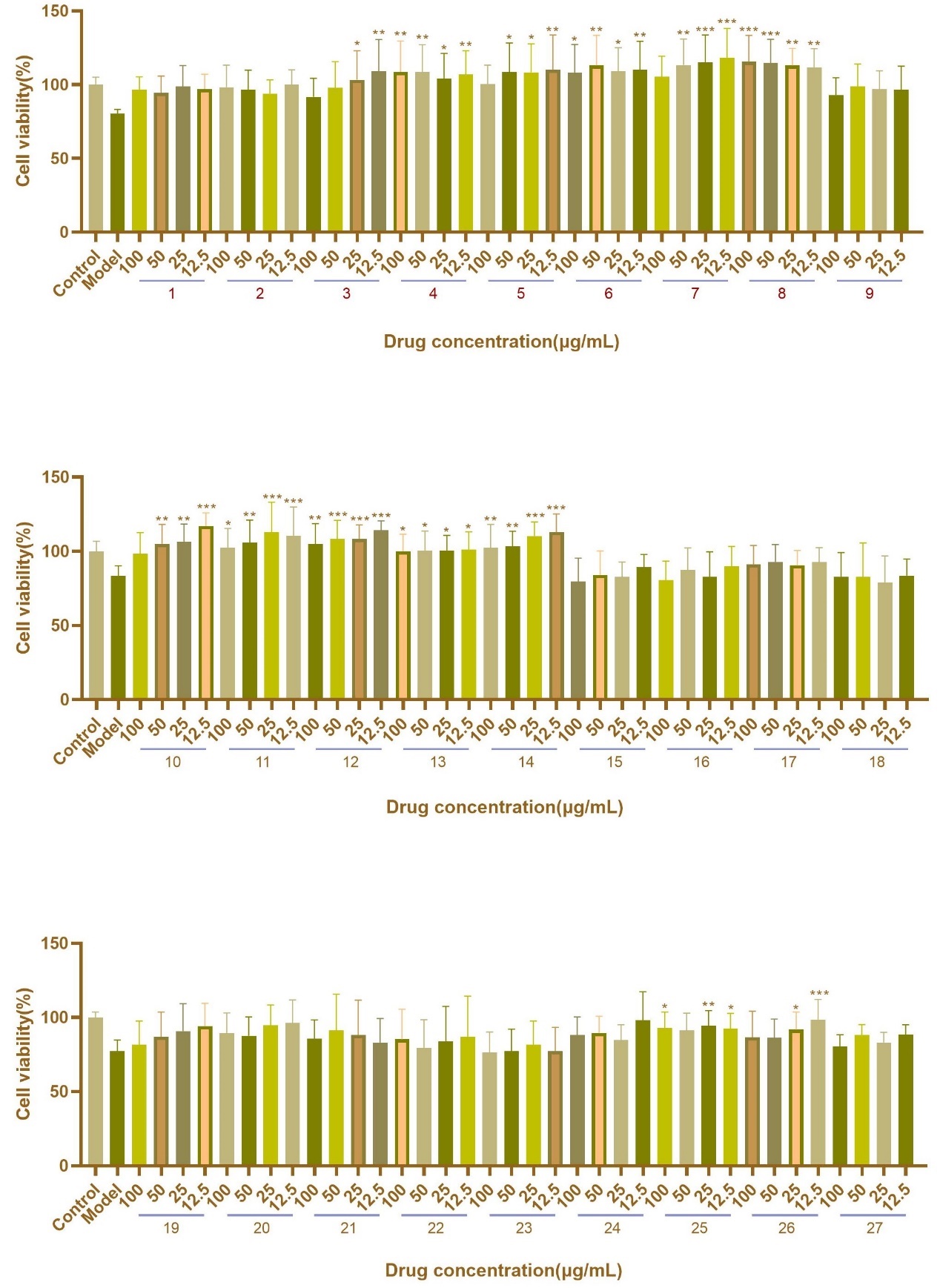


Figure. S2 Effects of ZYP orthogonal treatment on t-BHP oxidative damage model in HTR-8/SVneo cell. Values above represent the means of three experiments. Data shown represent means ± SD; * P < 0.05, ** P < 0.01, *** P < 0.001 compared to model group.


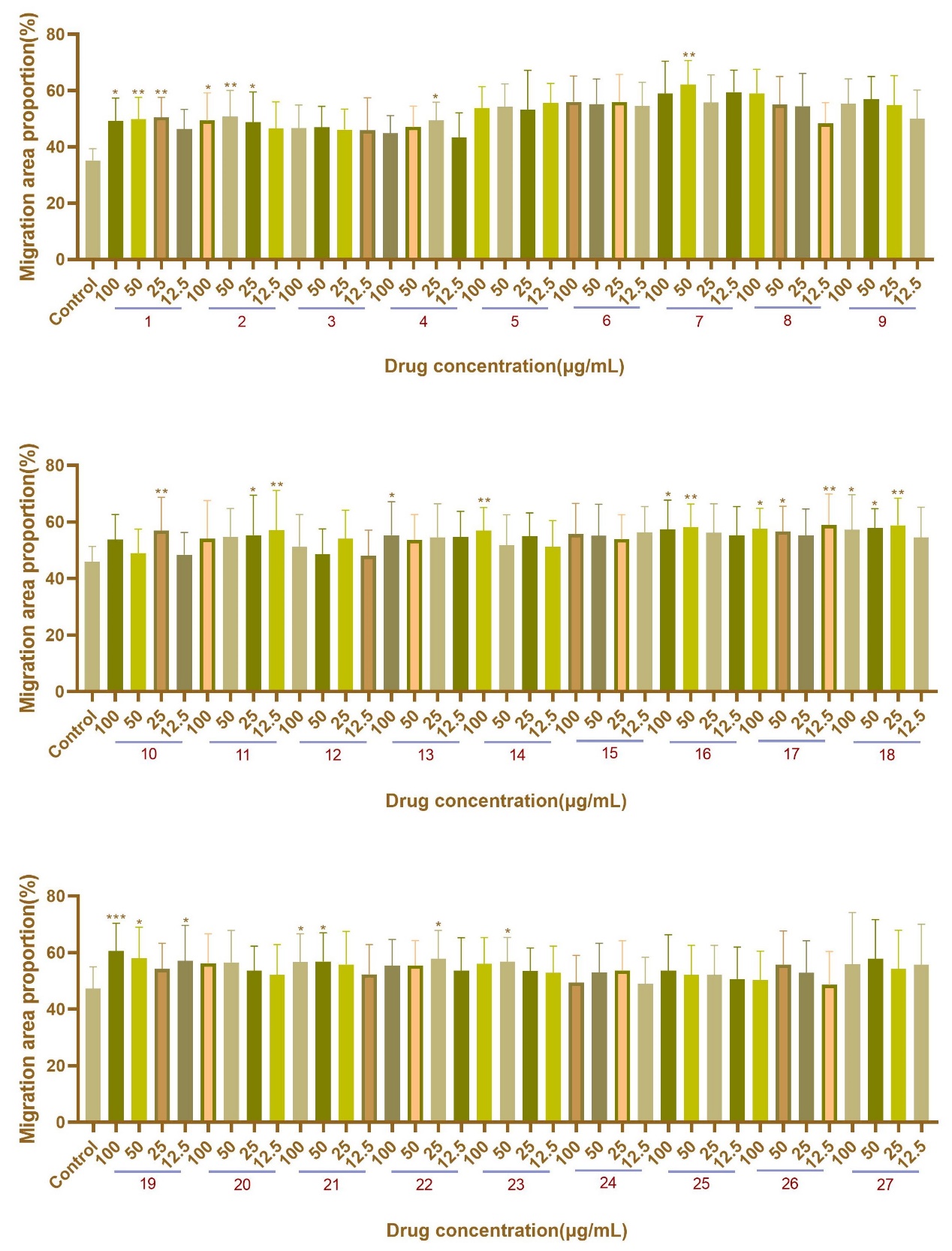


Figure. S3 Effects of ZYP orthogonal treatment on migration model in HTR-8/SVneo cell. Values above represent the means of three experiments. Data shown represent means ± SD; * P < 0.05, ** P < 0.01, *** P < 0.001 compared to control group.


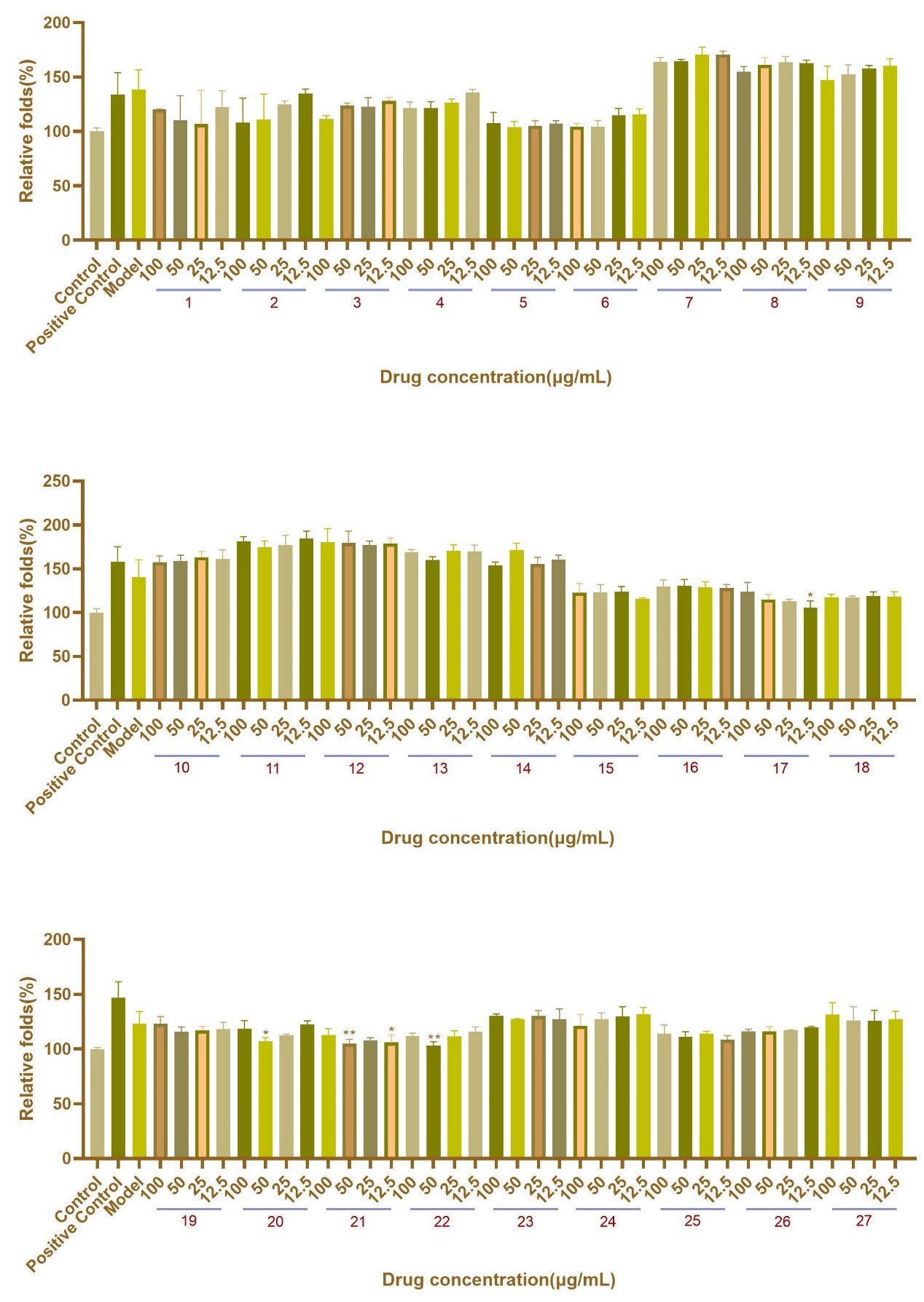


Figure. S4 Effects of ZYP orthogonal treatment on ROS model in HTR-8/SVneo cell. Values above represent the means of three experiments. Data shown represent means ± SD; * P < 0.05, ** P < 0.01, *** P < 0.001 compared to model group.
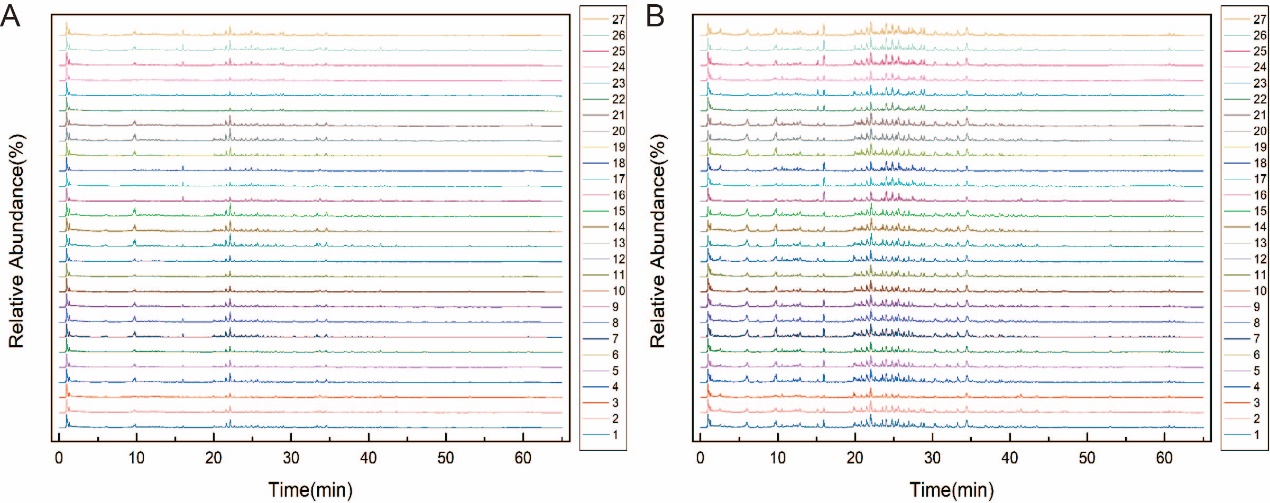


Figure. S5 UHPLC-MS spectrum of methanol extract of ZYP 27 orthogonal groups in positive**(A)** and negative**(B)** mode.

| Table S1 Primer sequence used in qPCR detection | | |
| --- | --- | --- |
| Gene | F | R |
| LIF | CGCCAATGCTCTCTTCATTTC | ATGGGAAGTCTGTCATGTTAGG |
| MMP-9 | CTGGAACTCACACGACATCTT | TCCACCTTGTTCACCTCATTT |
| TIMP-3 | GTACCGAGGCTTCAGTAAGATG | CTGTCAGCAGGTACTGGTATTT |
| EGF | AATAGTTATCCAGGATGCCC | ACGCAGTTCCCACCATCGTA |
| Actin | CATCCGTAAAGACCTCTATGCCAAC | ATGGAGCCACCGATCCACA |
| IFN-γ | GGCTGTTTCTGGCTGTTACT | TCTTCCACATCTATGCCACTTG |
| TNF-α | GGCAGGTCTACTTTGGAGTCATTGC | ACATTCGAGGCTCCAGTGAATTCGG |
| GAPDH | GGTGAAGGTCGGTGTGAACG | CTCGCTCCTGGAAGATGGTG |

LIF, leukemia inhibitory factor; MMP-9, matrix metalloproteinase-9; TIMP-3, tissue inhibitors of metalloproteinase-3; EGF, epidermal growth factor; IFN-γ, gamma-interferon; TNF-α, tumor necrosis factor-α; GAPDH, Glyceraldehyde-3-Phosphate Dehydrogenase.

| Table S2 Identification results of compounds in ZYP fractions (positive ion mode) | | | | | | | | |  |
| --- | --- | --- | --- | --- | --- | --- | --- | --- | --- |
| Peak No. | Identification | Molecular formula | Retention time/min | Measured [M+H]^+^  (m/z) | Theoretical  [M+H]^+^  (m/z) | Error (ppm) | LC/ESI–MS^n^ m/z  (% base peak) | Herb | Fraction |
| 1 | O-Cymene | C_10_H_14_ | 0.06 | 135.1158 | 135.1168 | -7.971 | 107(100), 93(62), 135(55) | SJS | 1-10 |
| 2 | Methyl palmatate | C_17_H_34_O_2_ | 0.26 | 271.2611 | 271.2632 | -7.435 | 103(100), 117(89), 149(79), 219(71) | TSZ DS | 1-12 |
| 3 | Bajijiasu | C_12_H_22_O_11_ | 0.98 | 365.1027 | 365.1054 | -7.512 | 203(100), 275(38), 347(30), 305(20) | BJT | 11, 12 |
| 4 | Nystose^*^ | C_24_H_42_O_21_ | 0.99 | 689.2059 | 689.2111 | -7.471 | 527(100), 509(23), 365(23), 347(21) | BJT | 13-16 |
| 5 | Isoleucine | C_6_H_13_NO_2_ | 1.51 | 132.101 | 132.1019 | -7.08 | 132(100), 114(18), 115(4) | DS | 1 |
| 6 | Sucrose | C_12_H_22_O_11_ | 1.83 | 365.1033 | 365.1054 | -5.841 | 203(100), 347(35), 185(26), 275(23) | BJT | 9, 10 |
| 7 | 5-hydroxy-2-hydroxymethylpyridine | C_6_H_7_NO_2_ | 1.88 | 126.054 | 126.055 | -7.656 | 108(100), 126(7) | DS | 2 |
| 8 | Betaine isomer | C_5_H_11_NO_2_ | 1.91 | 118.0856 | 118.0863 | -5.294 | 118(100), 59(3), 58(2) | / | 7 |
| 9 | Adenosine | C_10_H_13_N_5_O_4_ | 2.02 | 268.1022 | 268.104 | -6.715 | 136(100), 250(12) | RS DS | 3 |
| 10 | 5-Hydroxymethylfurfural^*^ | C_6_H_6_O_3_ | 2.02 | 127.0382 | 127.039 | -6.46 | 69(100), 109(92), 99(88), 81(15) | XD DS BZ | 6, 7 |
| 11 | L-Pyroglutamic acid | C_5_H_7_NO_3_ | 2.04 | 130.049 | 130.0499 | -6.918 | 84(100), 130(7), 102(2) | DS | 2, 4 |
| 12 | Codonopsinol | C_14_H_21_NO_5_ | 2.08 | 284.1476 | 284.1492 | -5.769 | 88(100), 266(72), 248(7), 210(7) | DS | 4, 5 |
| 13 | L-Pyroglutamic acid isomer | C_5_H_7_NO_3_ | 2.21 | 130.049 | 130.0499 | -6.687 | 84(100), 130(6), 102(3) | / | 6, 7 |
| 14 | 3-O-(3'-O-caffeoyl glucosyl)quinic acid/ 5-O-(3'-O-caffeoyl glucosyl)quinic acid/ 1-O-(3'-O-caffeoyl glucosyl)quinic acid | C_22_H_28_O_14_ | 2.22 | 517.1518 | 517.1552 | -6.559 | 163(100), 499(59), 325(34), 498(20) | DZ | 8 |
| 15 | Codonopiloside A | C_19_H_29_NO_9_ | 2.28 | 416.1891 | 416.1915 | -5.882 | 254(100), 398(71), 397(24), 236(19) | DS | 9 |
| 16 | Codonopsine | C_14_H_21_NO_4_ | 2.34 | 268.1525 | 268.1543 | -6.842 | 88(100), 250(20) | DS | 2 |
| 17 | Codonopsinol A | C_13_H_19_NO_5_ | 2.34 | 270.1318 | 270.1336 | -6.623 | 252(100), 234(18), 210(17), 126(14) | DS | 5, 6 |
| 18 | Betaine isomer | C_5_H_11_NO_2_ | 2.94 | 118.0854 | 118.0863 | -7.242 | 100(100), 118(24), 58(23), 88(17) | / | 3 |
| 19 | Neochlorogenic acid | C_16_H_18_O_9_ | 3.11 | 355.1 | 355.1024 | -6.698 | 163(100), 337(25), 267(17) | DZ DS | 4 |
| 20 | Acetic acid 3-[4-methyl-2-oxo-7-(3-phenyl-allyloxy)-2H-chromen-3-yl]-propyl ester/ 5-hydroxy-4-(1-methyl-1-p-tolylethoxy)-3,4,4a,9a-tetrahydro-1Hanthracene-2,9,10-trione | C_24_H_24_O_5_ | 3.77 | 393.1732 | 393.1697 | 8.926 | 375(100), 247(58), 239(43), 349(23) | BZ | 9 |
| 21 | Codonopiloside A isomer | C_19_H_29_NO_9_ | 3.94 | 416.189 | 416.1915 | -5.954 | 254(100), 236(21), 398(14) | / | 9 |
| 22 | Codonopsine isomer | C_14_H_21_NO_4_ | 4.28 | 268.1527 | 268.1543 | -6.282 | 88(100), 250(5), 118(3), 222(2) | / | 3, 4 |
| 23 | Threo-guaiacylglycerol/ borreriagenin | C_10_H_14_O_5_ | 4.43 | 215.0902 | 215.0914 | -5.626 | 197(100), 179(63), 109(6), 151(5) | DZ | 5 |
| 24 | 3-O-(3'-O-caffeoyl glucosyl)quinic acid/ 5-O-(3'-O-caffeoyl glucosyl)quinic acid/ 1-O-(3'-O-caffeoyl glucosyl)quinic acid | C_22_H_28_O_14_ | 4.52 | 517.1522 | 517.1552 | -5.843 | 163(100), 325(65), 307(13), 499(5) | DZ | 8 |
| 25 | 3-hydroxy-4-methoxycinnamaladehyde | C_10_H_10_O_3_ | 4.56 | 179.0693 | 179.0703 | -5.644 | 109(100), 151(74), 137(57), 123(28) | DZ | 5 |
| 26 | 3-hydroxy-1-(4-hydroxy-3-methoxyphenyl) propan-1-one isomer | C_10_H_12_O_4_ | 4.56 | 197.0797 | 197.0808 | -5.558 | 179(100), 151(31), 177(20), 109(19) | / | 5 |
| 27 | Betaine | C_5_H_11_NO_2_ | 4.67 | 118.0854 | 118.0863 | -7.242 | 100(100), 118(21), 58(21), 88(12) | DS | 2, 3 |
| 28 | Chlorogenic acid^*^ | C_16_H_18_O_9_ | 5.29 | 355.1001 | 355.1024 | -6.276 | 163(100), 267(24) | TSZ XD DZ DS | 4 |
| 29 | 6-methoxyquinoline-4-carbaldehyde | C_11_H_9_NO_2_ | 8.73 | 188.0695 | 188.0706 | -6.036 | 146(100), 144(10), 170(4), 171(1) | DS | 4 |
| 30 | 2,6-dihydroxycinnamic acid/ 4-(methoxycarbonyl) benzoic acid | C_9_H_8_O_4_ | 9.29 | 181.0483 | 181.0495 | -6.768 | 163(100), 136(5) | XD DZ BZ | 3 |
| 31 | Tryptophan | C_11_H_12_N_2_O_2_ | 9.39 | 205.0956 | 205.0972 | -7.578 | 177(100), 120(25), 187(16), 149(10) | DS | 2 |
| 32 | Pervoside A | C_16_H_20_O_8_ | 9.44 | 341.121 | 341.1231 | -6.227 | 323(100), 179(93), 197(57), 151(23) | DZ | 5 |
| 33 | Atractylenolide II isomer | C_15_H_20_O_2_ | 9.49 | 233.1522 | 233.1536 | -6.118 | 215(100), 205(36), 187(32), 191(23) | / | 6 |
| 34 | Cryptochlorogenic acid | C_16_H_18_O_9_ | 9.74 | 355.1 | 355.1024 | -6.614 | 163(100) | DZ DS | 3 |
| 35 | 4-methoxycinnamic acid isomer | C_10_H_10_O_3_ | 9.88 | 179.0694 | 179.0703 | -4.863 | 151(100), 137(78), 161(34), 133(16) | / | 9, 10 |
| 36 | 7-hydroxy-6-methoxy-coumarin | C_10_H_8_O_4_ | 10.04 | 193.0482 | 193.0495 | -6.969 | 165(100) | BJT BZ | 1 |
| 37 | Sweroside^*^ | C_16_H_22_O_9_ | 10.05 | 359.1318 | 359.1337 | -5.064 | 197(100) | XD DS | 8 |
| 38 | Kaempferol-3,7-O-diglucoside | C_27_H_30_O_16_ | 10.07 | 611.1572 | 611.1607 | -5.712 | 449(100), 287(44), 593(15) | DS | 7, 8 |
| 39 | Hydroxy-pinoresinol | C_20_H_22_O_7_ | 10.29 | 375.1419 | 375.1438 | -5.117 | 357(100), 233(20), 205(19) | DZ | 9 |
| 40 | Chlorogenic acid isomer | C_16_H_18_O_9_ | 10.37 | 355.0998 | 355.1024 | -2.599 | 337(100), 267(57), 309(48) | / | 2 |
| 41 | 4-methoxycinnamic acid | C_10_H_10_O_3_ | 10.91 | 179.0691 | 179.0703 | -6.314 | 161(100), 151(45), 137(36) | BZ | 1 |
| 42 | 3,4-Dicaffeoylquinic acid^*^ | C_25_H_24_O_12_ | 11.33 | 517.1307 | 517.1341 | -6.522 | 499(100), 499(24) | TSZ XD DZ | 4 |
| 43 | 8-Epiloganic acid | C_16_H_24_O_10_ | 11.33 | 377.1433 | 377.1442 | -2.422 | 243(100), 359(22), 331(4), 172(3) | SDH | 4 |
| 44 | Sweroside isomer | C_16_H_22_O_9_ | 11.35 | 359.1313 | 359.1337 | -6.679 | 197(100) | / | 2-7 |
| 45 | 4-methoxycinnamic acid isomer | C_10_H_10_O_3_ | 11.35 | 179.0692 | 179.0703 | -6.147 | 151(100), 109(92), 137(60), 161(23) | / | 2, 3 |
| 46 | 3-hydroxy-1-(4-hydroxy-3-methoxyphenyl) propan-1-one | C_10_H_12_O_4_ | 11.35 | 197.0796 | 197.0808 | -6.167 | 179(100), 127(52), 151(12) | BZ | 2, 3, 4 |
| 47 | Caffeic acid ethyl ester | C_11_H_12_O_4_ | 11.37 | 209.0795 | 209.0808 | -6.53 | 191(100), 177(63), 149(40), 181(13) | DZ BZ | 3 |
| 48 | Lisianthioside | C_32_H_44_O_18_ | 11.41 | 717.2553 | 717.26 | -6.679 | 698(100), 359(78) | XD | 2, 3 |
| 49 | 3-hydroxy-4-methoxycinnamaladehyde isomer | C_10_H_10_O_3_ | 11.47 | 179.0692 | 179.0703 | -5.979 | 109(100), 151(84), 137(60), 123(36) | / | 6 |
| 50 | Pyrogallol | C_6_H_6_O_3_ | 11.51 | 127.038 | 127.039 | -7.483 | 127(100), 97(94), 99(93), 85(50) | XD DZ | 2, 3 |
| 51 | Cinnamic acid | C_9_H_8_O_2_ | 11.84 | 149.0586 | 149.0597 | -7.152 | 131(100), 121(51), 149(13), 103(10) | TSZ | 1 |
| 52 | Dipsanoside A | C_66_H_90_O_37_ | 11.97 | 1475.5132 | 1475.5234 | -6.906 | 1377(100), 1103(99), 1294(91), 1440(85) | XD | 12 |
| 53 | Centaureidin isomer | C_18_H_16_O_8_ | 12.09 | 361.0892 | 361.0918 | -7.267 | 343(100), 331(44), 315(35), 247(16) | / | 2 |
| 54 | 7-hydroxy-coumarin isomer | C_9_H_6_O_3_ | 12.39 | 163.0381 | 163.039 | -5.156 | 145(100), 135(14) | / | 7 |
| 55 | (+)-1-hydroxy-2,6-bis-epi-pinoresinol | C_20_H_22_O_7_ | 12.44 | 375.1414 | 375.1438 | -6.503 | 357(100), 233(15), 205(14) | XD | 3 |
| 56 | Apigenin−6,8-di-C-pentoside | C_25_H_26_O_13_ | 12.53 | 535.1408 | 535.1446 | -7.114 | 499(100), 517(51), 517(39), 469(36) | AY | 4 |
| 57 | Balanophonin isomer | C_20_H_20_O_6_ | 12.62 | 357.131 | 357.1333 | -6.482 | 203(100), 311(76), 327(76), 339(59) | / | 3 |
| 58 | Rutin | C_27_H_30_O_16_ | 12.63 | 611.1571 | 611.1607 | -5.827 | 303(100), 593(31), 465(22), 594(13) | TSZ AY | 6, 7, 9 |
| 59 | Lanceolune A | C_11_H_10_O_4_ | 12.71 | 207.0638 | 207.0652 | -6.642 | 177(100), 189(13) | DS | 1 |
| 60 | Hyperoside^*^ | C_21_H_20_O_12_ | 12.71 | 465.0996 | 465.1028 | -6.756 | 303(100), 446(11), 447(8), 419(4) | TSZ DZ | 6, 7 |
| 61 | 3,5-Dicaffeoylquinic acid^*^ | C_25_H_24_O_12_ | 12.71 | 517.1307 | 517.1341 | -6.522 | 499(100), 469(51), 481(27), 451(16) | TSZ XD DZ | 6 |
| 62 | 3-O-Caffeoylquinic acid butyl ester | C_20_H_26_O_9_ | 12.73 | 411.1619 | 411.165 | -7.439 | 229(100), 381(46) | DS | 1 |
| 63 | Apigenin-6,8-di-C-pentoside isomer | C_25_H_26_O_13_ | 12.78 | 535.1409 | 535.1446 | -3.747 | 517(100), 499(88), 469(33), 481(28) | / | 6 |
| 64 | Cistanoside A/Jionoside A1/Jionoside A2 | C_36_H_48_O_20_ | 12.81 | 801.2766 | 801.2812 | -5.691 | 783(100), 782(85), 640(54), 764(40) | SDH | 8 |
| 65 | Uridine | C_9_H_12_N_2_O_6_ | 12.9 | 245.0793 | 245.0768 | 9.578 | 227(100), 151(99), 121(51), 199(51) | DZ BZ | 3 |
| 66 | Apigenin-6,8-di-C-pentoside isomer | C_25_H_26_O_13_ | 12.9 | 535.1411 | 535.1446 | -6.666 | 499(100), 517(89), 469(37), 481(37) | / | 5 |
| 67 | Quercetin^*^ | C_15_H_10_O_7_ | 12.91 | 303.0481 | 303.0478 | -6.036 | 257(100), 229(76), 285(56), 165(50) | TSZ DZ DS | 4 |
| 68 | Isoquercitrin | C_21_H_20_O_12_ | 12.91 | 465.0996 | 465.1028 | -6.692 | 303(100) | TSZ DZ | 4 |
| 69 | 2,3,5,4'-tetrahydroxystilbene-2-O-b-Dglucoside^*^ | C_20_H_22_O_9_ | 12.95 | 407.1309 | 407.1337 | -6.727 | 245(100), 388(10), 389(7), 227(3) | HSW | 3 |
| 70 | luteolin-7-O-β-D-glucopyranosyl-(1→6)-[(6'''-O-caffeoyl)-β-D-glucopyranoside] | C_36_H_36_O_19_ | 13.03 | 773.1884 | 773.1924 | -5.18 | 303(100), 755(55), 754(52), 471(43) | DS | 7 |
| 71 | Apigenin-O-rutinoside isomer | C_27_H_30_O_14_ | 13.04 | 579.1674 | 579.1708 | -5.995 | 561(100), 513(28), 543(25), 457(18) | / | 8 |
| 72 | Ginsenoside Rg2^*^ | C_42_H_72_O_13_ | 13.08 | 785.5002 | 785.5046 | -5.587 | 768(100), 623(29), 635(14), 406(12) | RS | 9 |
| 73 | kaempferol 3-O-rutinoside | C_27_H_30_O_15_ | 13.12 | 595.1616 | 595.1657 | -6.933 | 449(100), 287(25) | TSZ DZ | 5, 6 |
| 74 | Astragalin^*^ | C_21_H_20_O_11_ | 13.17 | 449.1048 | 449.1078 | -6.764 | 287(100) | TSZ | 3, 4, 5 |
| 75 | 4(R),15-epoxy-8β-hydroxyatractylenolideⅡ | C_15_H_20_O_4_ | 13.49 | 265.1419 | 265.1434 | -5.98 | 247(100), 229(14), 203(10), 175(10) | BZ | 4 |
| 76 | 7-hydroxy-coumarin isomer | C_9_H_6_O_3_ | 13.56 | 163.0379 | 163.039 | -6.567 | 145(100), 135(16), 163(7), 117(6) | / | 4 |
| 77 | Coumaroylquinic acid | C_16_H_18_O_8_ | 13.68 | 339.1055 | 339.1074 | -5.821 | 177(100), 321(16), 145(6), 195(4) | DZ | 6 |
| 78 | De-C-Cantleyoside | C_32_H_46_O_19_ | 13.75 | 735.2665 | 735.2706 | -5.611 | 573(100), 718(50), 717(32), 690(23) | XD | 7 |
| 79 | Kaempferol^*^ | C_15_H_10_O_6_ | 13.87 | 287.0531 | 287.055 | -6.67 | 241(100), 165(91), 213(89), 258(47) | TSZ DS | 3 |
| 80 | Atractylenolide V | C_15_H_20_O_4_ | 13.97 | 265.1417 | 265.1434 | -6.697 | 201(100), 247(77), 205(23) | BZ | 1 |
| 81 | Quercetin isomer | C_15_H_10_O_7_ | 14.01 | 303.048 | 303.0478 | -6.465 | 257(100), 229(98), 165(62), 285(61) | / | 3 |
| 82 | Apigenin-O-rutinoside | C_27_H_30_O_14_ | 14.08 | 579.1671 | 579.1708 | -6.426 | 433(100), 271(22) | AY | 4 |
| 83 | Epieucommiol | C_9_H_16_O_4_ | 14.22 | 189.1109 | 189.1121 | -6.745 | 171(100) | DZ | 1 |
| 84 | Apigenin-O-rutinoside isomer | C_27_H_30_O_14_ | 14.28 | 579.1671 | 579.1708 | -6.426 | 433(100), 271(23) | AY | 5 |
| 85 | Quercetin-3-O-rhamnosylgalactoside | C_27_H_30_O_16_ | 14.28 | 611.1568 | 611.1607 | -6.318 | 287(100), 449(45), 592(40), 383(24) | TSZ | 6 |
| 86 | Balanophonin | C_20_H_20_O_6_ | 14.52 | 357.1307 | 357.1333 | -7.154 | 339(100), 203(54), 327(36), 233(35) | DZ | 1 |
| 87 | Apigenin-O-glycuronide | C_21_H_18_O_11_ | 14.53 | 447.0887 | 447.0922 | -7.913 | 271(100) | AY | 3 |
| 88 | 7-hydroxy-coumarin isomer | C_9_H_6_O_3_ | 14.65 | 163.0378 | 163.039 | -6.996 | 145(100), 135(12), 163(8), 117(4) | / | 3, 5 |
| 89 | 4,5-Dicaffeoylquinic acid^*^ | C_25_H_24_O_12_ | 14.66 | 517.1296 | 517.1341 | -8.533 | 449(100), 498(21) | TSZ XD DZ | 2, 3 |
| 90 | Eucomegastigside A/ Eucomegastigside B/ Eriojaposide A | C_24_H_38_O_11_ | 14.9 | 503.245 | 503.2487 | -7.409 | 485(100), 281(53), 415(50), 457(49) | DZ | 4, 5 |
| 91 | Coumaroylquinic acid | C_16_H_18_O_8_ | 14.93 | 339.1054 | 339.1074 | -6.175 | 177(100), 321(21), 145(8), 195(3) | DZ | 4 |
| 92 | Tectoridin | C_22_H_22_O_11_ | 15 | 463.12 | 463.1235 | -7.467 | 301(100), 445(3), 444(2), 417(1) | DS | 3 |
| 93 | 1,2-dimethoxy-3-hydroxyanthraquinone isomer | C16H12O6 | 15.02 | 301.0686 | 301.0707 | -6.957 | 286(100), 287(6), 283(3), 255(1) | / | 3 |
| 94 | Dipsalignan C/ Dipsalignan D | C_21_H_24_O_9_ | 15.12 | 421.1463 | 421.1493 | -7.215 | 389(100), 407(41), 179(22) | XD | 3 |
| 95 | 2,3,5,4'-tetrahydroxystilbene-2-O-b-Dglucoside isomer | C_20_H_22_O_9_ | 15.12 | 407.1307 | 407.1337 | -7.243 | 389(100), 179(11), 407(11), 388(5) | / | 3 |
| 96 | Sylvestroside III/ sylvestroside IV | C_27_H_36_O_14_ | 15.16 | 585.2138 | 585.2178 | -6.787 | 423(100), 542(65), 422(50), 477(44) | XD | 6 |
| 97 | Ginsenoside Rg1^*^ | C_42_H_72_O_14_ | 15.17 | 801.4935 | 801.4995 | -7.502 | 720(100), 441(81), 784(71), 655(32) | RS | 11, 12 |
| 98 | Erythro-guaiacylglycerol | C_10_H_14_O_5_ | 15.38 | 215.09 | 215.0914 | -6.509 | 197(100), 155(61) | DZ | 1 |
| 99 | Eucophenoside | C_14_H_16_O_8_ | 15.39 | 313.0898 | 313.0918 | -6.528 | 151(100), 295(26), 169(6), 295(4) | DZ | 5 |
| 100 | (+)-Medioresinol 4-O-β-D-glucopyranoside | C_27_H_34_O_12_ | 15.6 | 551.208 | 551.2123 | -7.788 | 325(100), 533(93), 389(69), 516(23) | DZ | 3 |
| 101 | Rubiadin | C_15_H_10_O_4_ | 15.61 | 255.0635 | 255.0652 | -6.764 | 199(100), 137(74), 227(64), 237(51) | BJT | 1 |
| 102 | Tangshenoside V | C_21_H_26_O_12_ | 15.8 | 471.1462 | 471.1497 | -7.477 | 309(100), 147(98), 453(81), 291(59) | DS | 4, 5 |
| 103 | Lariciresinol | C_20_H_24_O_6_ | 15.83 | 361.1618 | 361.1646 | -7.573 | 343(100), 189(43), 317(39), 181(31) | DZ DS | 1 |
| 104 | Physcion or isomer | C_16_H_12_O_5_ | 16.44 | 285.0738 | 285.0758 | -6.875 | 270(100), 253(27), 225(16), 229(16) | BJT | 1 |
| 105 | 4-Caffoyl-5-coumaroylquinic acid | C_25_H_24_O_11_ | 16.45 | 501.1349 | 501.1391 | -8.477 | 483(100), 482(18) | TSZ | 2 |
| 106 | Quercetin isomer | C_15_H_10_O_7_ | 16.58 | 303.0478 | 303.0478 | -7.158 | 257(100), 229(76), 285(56), 165(50) | / | 1 |
| 107 | Luteolin | C_15_H_10_O_6_ | 16.67 | 287.053 | 287.055 | -6.879 | 153(100), 269(69), 241(57) | AY DZ DS BZ | 1 |
| 108 | 5,7,3',5'-Tetrahydroxy-6,4'-Dimethoxyflavone/ 5,7,4',5'-Tetrahydroxy−6,3'-Dimethoxyflavone/ Eupatolitin | C_17_H_14_O_8_ | 16.69 | 347.0736 | 347.0761 | -7.473 | 332(100), 333(17) | AY | 2 |
| 109 | Apigenin-O-glycuronide isomer | C_21_H_18_O_11_ | 16.94 | 447.089 | 447.0922 | -7.152 | 271(100) | AY | 4 |
| 110 | Baicalein | C_15_H_10_O_5_ | 17 | 271.0584 | 271.0601 | -6.124 | 253(100), 225(44), 103(25), 123(24) | DZ | 4 |
| 111 | 5,7,3',5'-Tetrahydroxy-6,4'-Dimethoxyflavone/ 5,7,4',5'-Tetrahydroxy−6,3'-Dimethoxyflavone/ Eupatolitin | C_17_H_14_O_8_ | 17.33 | 347.0737 | 347.0761 | -6.955 | 332(100), 333(10) | AY | 1 |
| 112 | Ginsenjilinol/ Ginsenoside Re5 | C_42_H_72_O_15_ | 17.35 | 817.4905 | 817.4944 | -4.719 | 732(100), 644(78), 740(73), 537(61) | RS | 8 |
| 113 | Syringyglycerol-β-syringaresinol ether 4'''-O-glucopyranoside | C_39_H_50_O_18_ | 17.45 | 807.3024 | 807.307 | -5.65 | 762(100), 764(16), 627(7), 777(7) | DZ | 6, 7 |
| 114 | 3-p-coumaroylquinic acid | C_16_H_18_O_8_ | 17.51 | 339.1053 | 339.1074 | -6.352 | 177(100), 321(16), 145(5), 195(4) | DZ | 3 |
| 115 | Quercetin isomer | C_15_H_10_O_7_ | 17.75 | 303.0477 | 303.0478 | -7.356 | 257(100), 285(68), 275(22), 274(22) | / | 2 |
| 116 | 1,3,6-trihydroxy-2-methoxyanthraquinone | C_15_H_10_O_6_ | 17.85 | 287.0529 | 287.055 | -7.297 | 287(100), 269(77), 259(40) | BJT | 2 |
| 117 | 8-methoxy-medioresinol | C_22_H_26_O_8_ | 17.9 | 419.1668 | 419.17 | -7.644 | 401(100), 369(17) | DZ | 1 |
| 118 | Eupatilin isomer | C_18_H_16_O_7_ | 18.19 | 345.0944 | 345.0969 | -7.213 | 330(100), 331(11) | / | 2 |
| 119 | Ginsenoside Rg6/F4 | C_42_H_70_O_12_ | 18.24 | 767.4896 | 767.494 | -5.79 | 423(100), 588(94), 749(59), 570(48) | RS | 9 |
| 120 | Jaceosidin^*^ | C_17_H_14_O_7_ | 18.26 | 331.079 | 331.0812 | -6.612 | 316(100) | AY | 1 |
| 121 | 6β-D-glucopyranosyloxy-5-hydroxy-7-methoxy-flavonone | C_22_H_22_O_10_ | 18.78 | 447.1254 | 447.1286 | -7.164 | 285(100) | BZ | 3, 4 |
| 122 | Ginsenoside Rg1 isomer | C_42_H_72_O_14_ | 18.79 | 801.4945 | 801.4995 | -6.205 | 784(100), 711(75), 701(70), 442(57) | / | 10 |
| 123 | Ginsenoside F2^*^ | C_42_H_72_O_13_ | 18.89 | 785.4983 | 785.5046 | -7.993 | 768(100), 725(21), 407(20), 325(15) | RS | 11 |
| 124 | Physcion or isomer | C_16_H_12_O_5_ | 18.94 | 285.0741 | 285.0758 | -5.788 | 270(100), 242(18), 271(14), 153(5) | BJT | 4 |
| 125 | Atractylenolide II isomer | C_15_H_20_O_2_ | 19.1 | 233.1519 | 233.1536 | -7.19 | 215(100), 187(25), 147(11) | / | 2 |
| 126 | Apigenin | C_15_H_10_O_5_ | 19.34 | 271.0584 | 271.0601 | -6.124 | 153(100) | TSZ DS BZ | 1 |
| 127 | 7-hydroxy-coumarin | C_9_H_6_O_3_ | 19.51 | 163.0378 | 163.039 | -7.119 | 145(100), 135(17) | DZ BZ | 2 |
| 128 | 1,2-dimethoxy-3-hydroxyanthraquinone | C_16_H_12_O_6_ | 19.99 | 301.0686 | 301.0707 | -6.758 | 286(100) | BJT DS | 1 |
| 129 | 1,6-dihydroxy-2-methoxyanthraquinone/ 1,3-dihydroxy-2-methoxyanthraquinone | C_15_H_10_O_5_ | 20.01 | 271.0584 | 271.0601 | -6.456 | 229(100) | BJT | 2 |
| 130 | 1,4a,5,7a-tetrahydro-7-hydroxymethyl-cyclopenta[c]pyran-4-carboxylic methyl ester isomer | C_11_H_14_O_4_ | 20.16 | 211.0953 | 211.0965 | -5.663 | 193(100), 179(92), 175(47), 169(39) | / | 5 |
| 131 | Akebia saponin X | C_76_H_124_O_40_ | 20.33 | 1677.7645 | 1677.7742 | -5.742 | 1620(100), 1532(36) | XD | 14 |
| 132 | Wogonin/ Oroxylin A | C_16_H_12_O_5_ | 20.44 | 285.0736 | 285.0758 | -7.717 | 270(100) | DZ BJT DS | 2 |
| 133 | Macranthoside B isomer | C_53_H_86_O_22_ | 20.48 | 1075.5601 | 1075.5684 | -7.708 | 1043(100), 1045(79), 994(26), 943(22) | / | 12 |
| 134 | Centaureidin | C_18_H_16_O_8_ | 20.63 | 361.0892 | 361.0918 | -7.267 | 346(100) | AY | 1 |
| 135 | Isorhamnetin^*^ | C_16_H_12_O_7_ | 20.65 | 317.0634 | 317.0656 | -2.189 | 302(100), 285(31), 299(18), 257(10) | TSZ | 1 |
| 136 | ​Diasesaminol | C_20_H_18_O_7_ | 20.94 | 371.1097 | 371.1125 | -7.597 | 353(100), 231(29) | TSZ | 2 |
| 137 | Macranthoidin A/ Dipsacus saponin VII | C_59_H_96_O_27_ | 21 | 1237.6145 | 1237.6212 | -5.392 | 1215(100), 1076(80), 769(64), 961(59) | XD | 11 |
| 138 | α-hederin isomer | C_41_H_66_O_12_ | 21.14 | 751.4575 | 751.4627 | -6.978 | 455(100), 437(78), 619(31), 584(14) | / | 11 |
| 139 | Oleanolic acid 28-O-β-D-glucopyranoside isomer | C_36_H_58_O_8_ | 21.18 | 619.4157 | 619.4204 | -7.741 | 438(100), 602(98), 435(93), 456(70) | / | 11 |
| 140 | Macranthoside B isomer | C_53_H_86_O_22_ | 21.18 | 1075.5618 | 1075.5684 | -6.118 | 1043(100), 752(75), 735(57), 834(54) | / | 11 |
| 141 | Nordamnacanthal | C_15_H_8_O_5_ | 21.91 | 269.0427 | 269.0444 | -6.541 | 213(100), 185(87), 251(51), 241(42) | BJT | 1 |
| 142 | Centaureidin isomer A | C_18_H_16_O_8_ | 22.3 | 361.0891 | 361.0918 | -7.516 | 346(100), 345(20), 347(3) | AY | 1 |
| 143 | Ginsenjilinol/ Ginsenoside Re5 | C_42_H_72_O_15_ | 22.47 | 817.4899 | 817.4944 | -5.539 | 457(100), 575(52), 421(47), 391(47) | RS | 7 |
| 144 | Centaureidin isomer B | C_18_H_16_O_8_ | 22.56 | 361.0892 | 361.0918 | -7.184 | 346(100), 328(58), 345(38), 311(10) | AY | 1 |
| 145 | HN saponin F isomer | C_41_H_66_O_13_ | 22.79 | 767.4528 | 767.4576 | -6.331 | 455(100), 437(38), 677(24), 750(20) | / | 10 |
| 146 | Syringaresinol | C_22_H_26_O_8_ | 23.3 | 419.1665 | 419.17 | -8.384 | 401(100), 286(66), 265(22) | DZ DS | 11, 12 |
| 147 | Macranthoside B | C_53_H_86_O_22_ | 23.55 | 1075.5629 | 1075.5684 | -5.095 | 874(100), 705(95), 650(88), 905(87) | XD | 10 |
| 148 | Physcion or isomer | C_16_H_12_O_5_ | 23.88 | 285.0739 | 285.0758 | -6.454 | 252(100), 270(83), 225(6), 253(6) | BJT | 1 |
| 149 | Ginsenoside Ra1/ Ra2 | C_58_H_98_O_26_ | 24.01 | 1233.6163 | 1233.6239 | -6.099 | 790(100), 467(55) | RS | 11 |
| 150 | 6-methylgingediol isomer | C_18_H_30_O_4_ | 24.08 | 311.2193 | 311.2217 | -7.57 | 293(100), 275(19) | / | 2 |
| 151 | Akebia saponin D^*^ | C_47_H_76_O_18_ | 24.17 | 929.5067 | 929.5104 | -4.079 | 883(100), 438(75), 345(73), 767(60) | XD | 8 |
| 152 | Ginsenoside Rg6/F4 | C_42_H_70_O_12_ | 24.32 | 767.4885 | 767.494 | -7.223 | 606(100), 444(53), 750(44), 714(35) | RS | 11 |
| 153 | HN saponin F | C_41_H_66_O_13_ | 24.4 | 767.4531 | 767.4576 | -5.848 | 455(100), 437(72), 409(17), 605(13) | XD | 9 |
| 154 | Ginsenoside Rf | C_42_H_72_O_14_ | 24.74 | 801.4948 | 801.4995 | -5.831 | 784(100), 783(96), 565(62), 598(55) | RS | 5 |
| 155 | Eupatilin isomer | C_18_H_16_O_7_ | 25.09 | 345.0945 | 345.0969 | -6.866 | 330(100), 331(6) | / | 1 |
| 156 | Caryophyllene oxide | C_15_H_24_O | 25.94 | 221.1885 | 221.19 | -6.7 | 203(100) | SJS BZ | 1 |
| 157 | Rubiadin-1-methyl ether | C_16_H_12_O_4_ | 26.04 | 269.0791 | 269.0808 | -6.449 | 254(100) | BJT | 1 |
| 158 | Asperosaponin IV | C_49_H_78_O_19_ | 26.31 | 971.5165 | 971.521 | -4.659 | 398(100), 559(96), 846(88), 809(81) | XD | 6 |
| 159 | Dipsacus saponin A | C_42_H_68_O_14_ | 26.34 | 797.4637 | 797.4682 | -5.647 | 455(100), 635(68), 680(32), 669(20) | XD | 6 |
| 160 | Wogonin/ Oroxylin A | C_16_H_12_O_5_ | 26.74 | 285.0737 | 285.0758 | -7.086 | 270(100), 271(5) | DZ BJT DS | 1, 2 |
| 161 | Eupatilin isomer | C_18_H_16_O_7_ | 26.77 | 345.0943 | 345.0969 | -7.474 | 330(100), 331(8), 327(4) | / | 2 |
| 162 | Ginsenoside Rg5/Rk1 | C_42_H_70_O_12_ | 26.84 | 767.4882 | 767.494 | -7.614 | 426(100), 588(81), 408(78), 570(41) | RS | 10 |
| 163 | Rhein | C_16_H_12_O_5_ | 27.5 | 285.074 | 285.0758 | -6.244 | 270(100), 242(15), 271(12), 153(5) | HSW | 1 |
| 164 | Torachrysone-O-(acetyl)-glucoside isomer | C_22_H_26_O_10_ | 28.3 | 451.1602 | 451.1599 | 0.768 | 395(100), 387(53), 433(51), 331(15) | / | 11, 12, 15 |
| 165 | Atractylenolide Ⅳ | C_17_H_22_O_4_ | 29.01 | 291.1573 | 291.1591 | -6.27 | 231(100), 185(73), 213(68) | BZ | 1 |
| 166 | 8β-ethoxyasterolid | C_17_H_24_O_3_ | 29.73 | 277.1782 | 277.1798 | -5.993 | 231(100), 249(92), 203(52), 137(42) | BZ | 1 |
| 167 | Ginsenoside Rg5/Rk1 | C_42_H_70_O_12_ | 29.81 | 767.4894 | 767.494 | -5.947 | 750(100), 407(92), 444(90), 588(82) | RS | 9 |
| 168 | Atractylenolide I^*^ | C_15_H_18_O_2_ | 30.34 | 231.1364 | 231.138 | -6.734 | 163(100), 185(72), 213(68), 203(61) | BZ | 1 |
| 169 | α-hederin isomer | C_41_H_66_O_12_ | 30.48 | 751.4567 | 751.4627 | -7.95 | 456(100), 437(73), 619(31), 409(15) | / | 11 |
| 170 | Dipsacus saponin C | C_64_H_104_O_30_ | 30.56 | 1353.6599 | 1353.6685 | -6.359 | 882(100) | XD | 11 |
| 171 | 6-methylgingediol | C_18_H_30_O_4_ | 31.03 | 311.2194 | 311.2217 | -7.377 | 293(100) | DS | 1 |
| 172 | Torachrysone-O-(acetyl)-glucoside | C_22_H_26_O_10_ | 31.28 | 451.1608 | 451.1599 | 2.12 | 395(100), 387(63), 433(54), 331(23) | HSW | 6, 7, 8 |
| 173 | Hyacinthin | C_8_H_8_O | 32.02 | 121.0639 | 121.0648 | -7.198 | 93(100), 121(13) | XD SJS BJT | 3, 4, 5 |
| 174 | Atractylenolactam | C_15_H_19_NO | 32.85 | 230.1524 | 230.1539 | -6.521 | 185(100), 202(92), 160(71), 174(71) | BZ | 1 |
| 175 | 2α,3α-dihydroxy-24-nor-4(23),12- oleanadien-28-oic acid | C_29_H_44_O_4_ | 32.9 | 457.3272 | 457.3312 | -8.738 | 439(100), 421(34), 411(23) | DZ | 2 |
| 176 | Dipsacus saponin B | C_59_H_96_O_26_ | 33.04 | 1221.62 | 1221.6263 | -5.124 | 1087(100) | XD | 10 |
| 177 | α-hederin isomer | C_41_H_66_O_12_ | 33.04 | 751.4579 | 751.4627 | -6.406 | 455(100), 437(78), 409(20), 279(12) | / | 10 |
| 178 | (3β,​4α)​-23-​hydroxy-​3-​[(O-​β-​D-​xylopyranosyl-​(1→4)​-​O-​β-​D-​glucopyranosyl-​(1→4)​-​O-​β-​D-​glucopyranosyl-​(1→3)​-​O-​6-​deoxy-​α-​L-​mannopyranosyl-​(1→2)​-​α-​L-​arabinopyranosyl)​oxy]​-​Olean-​12-​en-​28-​oic acid | C_58_H_94_O_26_ | 33.3 | 1207.6043 | 1207.6106 | -5.266 | 641(100), 1189(65) | XD | 10 |
| 179 | Emodin^*^ | C_15_H_10_O_5_ | 33.53 | 271.0582 | 271.0601 | -6.899 | 229(100), 201(11) | DS HSW | 1 |
| 180 | 3-O-α-l-arabinopyranosyl oleanolic acid isomer | C_47_H_76_O_17_ | 33.54 | 913.5099 | 913.5155 | -6.171 | 600(100), 841(79), 787(70), 411(24) | XD | 6 |
| 181 | Dipsacus saponin A isomer | C_42_H_68_O_14_ | 33.92 | 797.4631 | 797.4682 | -6.336 | 363(100), 701(74), 599(68), 441(67) | / | 5 |
| 182 | Palmitic acid | C_16_H_32_O_2_ | 34.1 | 279.23 | 279.2319 | -6.829 | 261(100), 243(48), 165(20), 163(17) | XD BJT BZ | 1 |
| 183 | Dipsacus saponin R | C_53_H_86_O_21_ | 34.61 | 1059.5675 | 1059.5734 | -5.602 | 898(100), 703(80), 624(30) | XD | 9 |
| 184 | 3-O-α-l-arabinopyranosyl oleanolic acid | C_47_H_76_O_17_ | 34.65 | 913.5112 | 913.5155 | -4.77 | 865(100), 620(89), 751(76), 438(64) | XD | 7, 8 |
| 185 | α-hederin isomer | C_41_H_66_O_12_ | 34.73 | 751.4585 | 751.4627 | -5.594 | 455(100), 437(70), 619(26), 425(10) | / | 7, 9 |
| 186 | Oleanolic acid 28-O-β-D-glucopyranoside isomer | C_36_H_58_O_8_ | 34.87 | 619.4166 | 619.4204 | -6.272 | 602(100), 435(78), 409(60), 442(47) | / | 7 |
| 187 | α-Linolenic acid | C_18_H_30_O_2_ | 35.26 | 279.23 | 279.2319 | -6.721 | 261(100), 243(51), 95(22), 209(20) | BJT | 1 |
| 188 | HN saponin F isomer | C_41_H_66_O_13_ | 35.27 | 767.4529 | 767.4576 | -6.096 | 438(100), 455(65), 749(32), 351(15) | / | 5 |
| 189 | Dipsacus saponin N | C_46_H_74_O_16_ | 35.53 | 883.5003 | 883.505 | -5.266 | 522(100), 751(96), 438(62), 865(49) | XD | 5, 6 |
| 190 | α-hederin isomer | C_41_H_66_O_12_ | 35.59 | 751.4581 | 751.4627 | -6.087 | 455(100), 438(56), 605(18), 619(16) | / | 5 |
| 191 | Trichosanic acid | C_18_H_30_O_2_ | 36.15 | 279.23 | 279.2319 | -6.506 | 261(100), 243(40), 137(15), 123(14) | BJT | 1 |
| 192 | Coronaric acid | C_18_H_32_O_3_ | 36.22 | 297.2404 | 297.2424 | -7.036 | 279(100) | DS | 1 |
| 193 | Atractylenolide II^*^ | C_15_H_20_O_2_ | 36.27 | 233.152 | 233.1536 | -6.976 | 215(100), 187(57), 151(40) | DS BZ | 1 |
| 194 | α-hederin | C_41_H_66_O_12_ | 36.34 | 751.4576 | 751.4627 | -6.739 | 437(100), 456(100), 619(26), 409(22) | XD | 3 |
| 195 | Oleanolic acid 28-O-β-D-glucopyranoside | C_36_H_58_O_8_ | 36.39 | 619.4161 | 619.4204 | -7.047 | 436(100), 610(42), 601(41), 437(41) | RS | 3 |
| 196 | Cauloside A | C_35_H_56_O_8_ | 41.02 | 605.4006 | 605.4048 | -6.979 | 560(100), 559(20), 437(11), 189(7) | XD | 7 |
| 197 | Ajugoside/ Reptoside/ 8-epi-loganin | C_17_H_26_O_10_ | 41.78 | 391.1616 | 391.1599 | 4.465 | 382(100), 253(78), 237(28), 279(19) | DZ | 6, 10 |
| 198 | Linolenic acid | C_18_H_30_O_2_ | 42.04 | 279.2297 | 279.2319 | -7.581 | 261(100), 243(74), 137(42), 123(39) | BJT | 1 |
| 199 | Palmitic acid isomer | C_16_H_32_O_2_ | 42.4 | 279.2299 | 279.2319 | -7.151 | 261(100), 243(62), 223(43), 209(30) | BJT | 1 |
| 200 | Methyl pentadecanoate | C_18_H_30_O_2_ | 42.81 | 279.2299 | 279.2319 | -6.936 | 243(100), 261(98), 95(38), 195(38) | TSZ | 1 |
| 201 | Dibutyl terephthalate | C_16_H_22_O_4_ | 43.56 | 301.1392 | 301.141 | -6.111 | 245(100), 132(24), 265(18), 283(17) | DS | 19, 20 |
| 202 | 8β-ethoxyasterolid isomer | C_17_H_24_O_3_ | 45.62 | 277.1777 | 277.1798 | -7.544 | 235(100), 177(8), 259(3), 137(3) | / | 3 |
| 203 | 8β-ethoxyasterolid isomer | C_17_H_24_O_3_ | 45.91 | 277.1777 | 277.1798 | -7.544 | 235(100), 149(2), 173(1), 145(1) | / | 4, 7, 10 |
| 204 | 8β-ethoxyasterolid isomer | C_17_H_24_O_3_ | 46.34 | 277.1777 | 277.1798 | -7.653 | 235(100) | / | 9 |
| 205 | Linolenic acid | C_18_H_30_O_2_ | 47.43 | 279.2299 | 279.2319 | -7.151 | 261(100), 183(44), 243(27), 233(16) | BJT | 1 |
| 206 | 6-(2-dodecyloxy-propionyloxy)-3,8-dithia-cyclopenta[a]indene-2-carboxylic acid 4'-octyloxy-biphenyl-4-yl ester/ 6-(2-octyloxy-propionyloxy)-3,8-dithia-cyclopenta[a]indene-2-carboxylic acid 4'-dodecyloxy-biphenyl-4-yl ester | C_46_H_58_O_6_S_2_ | 47.54 | 771.3718 | 771.3748 | -3.873 | 441(100), 353(42), 669(20) | BZ | 1 |
| 207 | Oleic acid | C_18_H_34_O_2_ | 48.53 | 283.2611 | 283.2632 | -7.438 | 265(100), 247(25) | BJT | 1 |
| 208 | Artselaenin C | C_9_H_12_O_4_ | 53.07 | 185.0795 | 185.0808 | -7.377 | 129(100) | DZ | 2, 3 |
| 209 | Eucommidiol | C_9_H_12_O_4_ | 53.43 | 185.0796 | 185.0808 | -6.891 | 129(100), 167(1), 111(1), 143(1) | DZ | 4, 6-8 |
| 210 | Artselaenin C isomer | C_9_H_12_O_4_ | 53.97 | 185.0795 | 185.0808 | -7.161 | 129(100), 111(1), 144(1) | / | 5, 9 |
| 211 | 1,4a,5,7a-tetrahydro-7-hydroxymethyl-cyclopenta[c]pyran-4-carboxylic methyl ester | C_11_H_14_O_4_ | 55.18 | 211.095 | 211.0965 | -7.226 | 127(100), 85(62), 97(43) | DZ | 1 |
| 212 | Azelaic acid | C_9_H_16_O_4_ | 55.75 | 189.1107 | 189.1121 | -7.485 | 171(100), 153(21), 127(13) | AY DS | 2 |
| 213 | Biatractylenolide | C_30_H_38_O_4_ | 55.84 | 463.2803 | 463.2843 | -8.669 | 231(100), 233(53), 445(21) | BZ | 1 |
| 214 | Eucommiol | C_9_H_16_O_4_ | 56.08 | 189.1105 | 189.1121 | -8.648 | 171(100), 153(21), 125(15), 85(14) | DZ | 4, 6-10 |
| 215 | Foliasalacioside B1/ Eucomegastigside C/ Eucomegastigside D | C_24_H_40_O_11_ | 57.83 | 505.2625 | 505.2643 | -3.559 | 423(100) | DZ | 4, 5 |
| 216 | Δ7-stigmasterol | C_29_H_46_O | 58.15 | 411.3586 | 411.3621 | -8.661 | 393(100), 163(27), 239(20) | DS | 2 |
| 217 | 1,3-linolein-2-olein isomer | C_57_H_100_O_6_ | 61.97 | 903.7346 | 903.7412 | -8.167 | 600(100), 626(75), 604(42), 578(31) | / | 18 |
| 218 | 1,3-linolein-2-olein | C_57_H_100_O_6_ | 63.13 | 903.7346 | 903.7412 | -6.652 | 624(100), 622(69), 600(25), 602(20) | DS | 14 |
| 219 | Ginsenoside Rs6/ Rs7 | C_38_H_62_O_9_ | 64.05 | 663.4473 | 663.4467 | 1.01 | 608(100), 552(68), 495(22) | RS | 11, 12 |
| 220 | Stigmasta-5,22-dien-3-one | C_29_H_46_O | 64.24 | 411.3582 | 411.3621 | -9.633 | 275(100), 259(84), 164(52), 394(50) | DS | 1 |
| 221 | Codonocerebroside A | C_46_H_87_NO_10_ | 64.59 | 814.634 | 814.6403 | -7.739 | 383(100), 468(77), 355(52), 735(41) | DS | 17 |

| Table S3 Identification results of compounds in ZYP fractions (negative ion mode) | | | | | | | | |  |
| --- | --- | --- | --- | --- | --- | --- | --- | --- | --- |
| Peak No. | Identification | Molecular formula | Retention time/min | Measured [M-H]^-^  (m/z) | Theoretical  [M-H]^-^  (m/z) | Error (ppm) | LC/ESI–MS^n^ m/z  (% base peak) | Herb | Fraction |
| 222 | 8-O-4′ diferulic acid | C_14_H_10_Cl_4_ | 0.93 | 316.946 | 316.9453 | 2.09 | 249(100), 181(18), 233(12), 113(6) | DS | 1, 3, 5, 7, 8 |
| 223 | D-mannitol | C_6_H_14_O_6_ | 0.94 | 181.0707 | 181.0707 | -0.025 | 163(100), 101(58), 119(46), 89(43) | BZ | 6 |
| 224 | Mannose | C_6_H_12_O_6_ | 0.95 | 179.0551 | 179.055 | 0.645 | 161(100), 131(8), 89(6), 97(6) | BJT DS | 1-7 |
| 225 | Hypoxanthine | C_5_H_4_N_4_O | 0.95 | 135.0293 | 135.0301 | -6.349 | 117(100), 75(69), 89(25), 71(2) | DS | 3 |
| 226 | Raffinose | C_18_H_32_O_16_ | 0.96 | 503.1592 | 503.1607 | -2.824 | 485(100), 323(60), 341(46), 179(64) | DS | 13, 14, 15 |
| 227 | Quinic acid isomer | C_7_H_12_O_6_ | 0.99 | 191.0549 | 191.055 | -0.652 | 127(100), 85(77), 173(64), 93(57) | / | 4, 5 |
| 228 | Isocitric acid | C_6_H_8_O_7_ | 1 | 191.0186 | 191.0186 | -0.309 | 85(100), 173(28), 101(18), 147(13) | DZ | 3 |
| 229 | Fumaric acid | C_4_H_4_O_4_ | 1.05 | 115.0032 | 115.0026 | 5.347 | 71(100), 115(1) | BJT | 2 |
| 230 | Malic acid | C_4_H_6_O_5_ | 1.07 | 133.0136 | 133.0131 | 3.159 | 115(100), 89(23), 71(7), 87(4) | BJT | 1, 2, 3 |
| 231 | Quinic acid | C_7_H_12_O_6_ | 1.17 | 191.055 | 191.055 | -0.128 | 101(100), 145(46), 129(37), 173(9) | AY | 2 |
| 232 | Yopaaoside C/isomer / Digiferruginol-11-O-β-primeveroside | C_17_H_26_O_12_ | 1.25 | 421.1328 | 421.1341 | -3.093 | 375(100), 403(4), 305(3), 385(3) | BJT | 5 |
| 233 | Succinic acid | C_4_H_6_O_4_ | 1.32 | 117.0188 | 117.0182 | 4.656 | 73(100), 99(40), 75(18), 117(6) | BJT DS BZ | 1 |
| 234 | Gallic acid | C_7_H_6_O_5_ | 1.47 | 169.0134 | 169.0131 | 1.54 | 125(100) | DZ HSW | 2 |
| 235 | Deacetylasperuloside | C_16_H_20_O_10_ | 1.55 | 371.0963 | 371.0973 | -2.676 | 163(100), 325(96), 119(3), 353(2) | BJT | 3 |
| 236 | p-coumaric acid isomer | C_9_H_8_O_3_ | 1.63 | 163.0393 | 163.039 | 1.775 | 119(100), 145(2), 85(1), 101(1) | TSZ RS DZ | 3 |
| 237 | 5-Hydroxy-2-furaldehyde | C_5_H_4_O_3_ | 1.97 | 111.0083 | 111.0077 | 5.671 | 67(100), 83(15), 93(4), 65(2) | DS | 2 |
| 238 | Catalpol | C_15_H_22_O_10_ | 2.12 | 361.1119 | 361.1129 | -2.889 | 315(100), 343(51), 153(35), 199(30) | DZ | 3 |
| 239 | Quinic acid isomer | C_7_H_12_O_6_ | 2.13 | 191.055 | 191.055 | -0.024 | 101(100), 145(39), 129(34), 173(8) | AY | 2 |
| 240 | Dipsaicin | C_14_H_18_O_9_ | 2.16 | 329.0862 | 329.0867 | -1.515 | 167(100), 285(15), 311(7), 203(6) | XD | 3 |
| 241 | 3,4-dihydroxy benzoic acid | C_7_H_6_O_4_ | 2.21 | 153.0185 | 153.0182 | 1.992 | 109(100), 135(1), 81(1) | DZ | 1, 2 |
| 242 | 2,6-dimethoxyquinone isomer | C_8_H_8_O_4_ | 2.25 | 167.0341 | 167.0339 | 1.046 | 123(100), 149(9), 125(4), 69(3) | / | 1 |
| 243 | Geniposidic acid^*^ | C_16_H_22_O_10_ | 2.5 | 373.1117 | 373.1129 | -3.225 | 211(100), 123(61), 167(36) | DZ | 4, 5 |
| 244 | Maleic acid | C_4_H_4_O_4_ | 2.7 | 115.0031 | 115.0026 | 4.564 | 71(100), 87(39), 97(20), 73(11) | DS | 1 |
| 245 | Acteoside | C_26_H_40_O_17_ | 2.94 | 623.2157 | 623.2182 | -3.973 | 461(100), 477(70), 459(33), 315(8) | SDH | 11 |
| 246 | Glucosyringic acid | C_15_H_20_O_10_ | 3.1 | 359.0961 | 359.0973 | -3.183 | 197(100), 196(11), 182(5), 313(4) | DZ | 3 |
| 247 | Deacetyl asperulosidic acid | C_16_H_22_O_11_ | 3.3 | 389.1066 | 389.1078 | -3.181 | 345(100), 183(14), 209(13), 165(6) | DZ BJT | 5 |
| 248 | 2-furancarboxylic acid | C_5_H_4_O_3_ | 3.39 | 111.0082 | 111.0077 | 4.41 | 67(100), 83(7), 111(6), 93(4) | DS | 1, 2 |
| 249 | Deacetylasperuloside isomer | C_16_H_20_O_10_ | 3.57 | 371.0962 | 371.0973 | -2.919 | 163(100), 325(94), 119(2) | / | 3 |
| 250 | p-coumaric acid glucoside isomer | C_15_H_18_O_8_ | 3.57 | 325.091 | 325.0918 | -2.35 | 163(100), 187(9), 119(7), 307(7) | / | 3 |
| 251 | 2,6-dimethoxyquinone isomer | C_8_H_8_O_4_ | 3.6 | 167.0341 | 167.0339 | 1.046 | 123(100), 149(2), 125(1) | / | 1 |
| 252 | Quinic acid isomer | C_7_H_12_O_6_ | 3.65 | 191.0551 | 191.055 | 0.186 | 127(100), 85(84), 93(61), 173(52) | / | 3 |
| 253 | 3-(3,4-Dihydroxyphenyl)propionic acid isomer | C_9_H_10_O_4_ | 3.66 | 181.0495 | 181.0495 | -0.361 | 163(100), 137(19), 135(8), 123(3) | / | 2 |
| 254 | 2,6-dimethoxyquinone | C_8_H_8_O_4_ | 3.85 | 167.0341 | 167.0339 | 1.106 | 123(100), 95(2), 139(1), 149(1) | BZ | 1 |
| 255 | 4-hydroxy benzoic acid | C_7_H_6_O_3_ | 3.91 | 137.0237 | 137.0233 | 2.55 | 93(100), 109(46), 107(42), 119(20) | DS | 4 |
| 256 | Decaffeoyl-verbascoside isomer | C_20_H_30_O_12_ | 4 | 461.1637 | 461.1654 | -3.519 | 315(100), 135(58), 415(29), 443(22) | / | 5 |
| 257 | Protocatechuic acid^*^ | C_7_H_6_O_4_ | 4.03 | 153.0185 | 153.0182 | 1.665 | 109(100), 108(3) | AY DZ BZ | 1 |
| 258 | 3-hydroxybenzoic acid | C_7_H_6_O_3_ | 4.08 | 137.0236 | 137.0233 | 2.112 | 93(100), 95(15), 109(14), 137(11) | DZ | 2, 3, 5 |
| 259 | Deacetylasperuloside isomer | C_16_H_20_O_10_ | 4.14 | 371.0963 | 371.0973 | -2.757 | 191(100), 147(80), 163(57), 209(35) | / | 4 |
| 260 | Salicylic acid | C_7_H_6_O_3_ | 4.23 | 137.0237 | 137.0233 | 2.769 | 93(100), 137(3), 109(1) | RS | 1 |
| 261 | Ulmoside C | C_14_H_18_O_10_ | 4.31 | 345.0806 | 345.0816 | -2.878 | 183(100), 124(3), 255(3), 327(2) | DZ | 4 |
| 262 | Aucubin isomer | C_15_H_22_O_9_ | 4.63 | 345.1169 | 345.118 | -3.096 | 299(100), 327(4), 269(4), 283(3) | / | 3 |
| 263 | Periplobiose isomer | C_13_H_24_O_9_ | 4.81 | 323.1329 | 323.1337 | -2.255 | 119(100), 277(85), 179(80), 113(78) | / | 4 |
| 264 | p-hydroxycinnamic acid(P-Coumaric Acid) | C_9_H_8_O_3_ | 4.91 | 163.0392 | 163.039 | 1.284 | 163(100), 119(56), 135(50), 145(50) | RS | 1 |
| 265 | Syringaldehyde | C_9_H_10_O_4_ | 5.21 | 181.0496 | 181.0495 | 0.302 | 137(100), 122(38), 163(31), 123(15) | DS | 1 |
| 266 | Catechin | C_15_H_14_O_6_ | 5.23 | 289.07 | 289.0707 | -2.368 | 215(100), 245(79), 173(60), 151(32) | DZ | 2 |
| 267 | Ferulic acid isomer | C_10_H_10_O_4_ | 5.63 | 193.0496 | 193.0495 | 0.128 | 149(100), 178(59), 134(35), 85(6) | / | 3 |
| 268 | Quinic acid isomer | C_7_H_12_O_6_ | 6.02 | 191.055 | 191.055 | -0.024 | 127(100), 85(80), 173(74), 93(63) | / | 3 |
| 269 | Yopaaoside C/isomer / Digiferruginol-11-O-β-primeveroside | C_17_H_26_O_12_ | 6.06 | 421.1327 | 421.1341 | -3.164 | 375(100) | BJT | 5 |
| 270 | Vanillin | C_8_H_8_O_3_ | 6.24 | 151.0392 | 151.039 | 1.651 | 109(100), 151(69), 136(44), 107(8) | DS | 1 |
| 271 | Esculetin isomer | C_9_H_6_O_4_ | 6.32 | 177.0183 | 177.0182 | 0.253 | 133(100), 177(17), 149(9), 105(9) | / | 1 |
| 272 | Asperulosidic acid | C_18_H_24_O_12_ | 6.54 | 431.117 | 431.1184 | -3.253 | 251(100), 165(28), 339(19), 269(18) | BJT | 3, 4 |
| 273 | Protocatechuic acid methyl ester | C_8_H_8_O_4_ | 6.71 | 167.0341 | 167.0339 | 1.046 | 123(100), 152(62), 108(12), 109(3) | DZ | 1 |
| 274 | p-hydroxy benzaldehyde | C_7_H_6_O_2_ | 7.05 | 121.0288 | 121.0284 | 3.586 | 80(100), 92(55), 106(28) | DS | 1, 2, 4-8 |
| 275 | Shikimic acid | C_7_H_10_O_5_ | 7.53 | 173.0446 | 173.0444 | 1.041 | 93(100), 111(53), 155(51), 71(23) | DS | 3 |
| 276 | p-coumaric acid isomer | C_9_H_8_O_3_ | 7.57 | 163.0393 | 163.039 | 1.898 | 119(100) | TSZ RS DZ | 3 |
| 277 | p-coumaric acid glucoside | C_15_H_18_O_8_ | 7.59 | 325.0911 | 325.0918 | -2.258 | 163(100), 119(12), 265(4), 235(3) | DS | 3 |
| 278 | Quercetin-3-O-β-D-glucopyranoside (1→2)-β-D-glucopyranoside | C_27_H_30_O_17_ | 7.93 | 625.1379 | 625.1399 | -3.176 | 463(100), 301(37), 462(25), 343(7) | DZ | 9 |
| 279 | (-)-olivil-4'-O-β-D-glucopyranoside isomer | C_26_H_34_O_12_ | 8.01 | 537.1944 | 537.1967 | -4.194 | 375(100), 491(2), 501(2), 327(2) | / | 4, 5 |
| 280 | Periplobiose | C_13_H_24_O_9_ | 8.12 | 323.1326 | 323.1337 | -3.183 | 101(100), 113(85), 119(78), 161(64) | DZ | 4, 5 |
| 281 | Vanillic acid | C_8_H_8_O_4_ | 8.18 | 167.0341 | 167.0339 | 1.226 | 125(100), 123(100), 151(72), 139(19) | RS XD DZ DS | 1 |
| 282 | Esculetin | C_9_H_6_O_4_ | 8.26 | 177.0183 | 177.0182 | 0.084 | 149(100), 133(98), 177(49), 105(44) | AY | 1 |
| 283 | Teaberry oil | C_8_H_8_O_3_ | 8.3 | 151.0392 | 151.039 | 1.717 | 123(100), 151(88), 136(85), 107(46) | SJS | 1 |
| 284 | Syringic acid | C_9_H_10_O_5_ | 8.34 | 197.0443 | 197.0444 | -0.862 | 153(100), 182(95), 179(12), 138(11) | DS | 1 |
| 285 | Monotropein | C_16_H_22_O_11_ | 8.42 | 389.1066 | 389.1078 | -3.258 | 345(100), 209(36), 121(16), 165(14) | BJT | 5 |
| 286 | Asperulosidic acid ethyl ester/ 3,4-dihydro-3β-ethoxyasperuloside | C_20_H_28_O_12_ | 8.55 | 459.1484 | 459.1497 | -2.75 | 163(100), 265(71), 145(38), 307(33) | DZ | 6 |
| 287 | Quercetin-3-O-β-D-galactoside-7-O-β-D-glucoside | C_27_H_30_O_17_ | 8.64 | 625.1376 | 625.1399 | -3.768 | 463(100), 301(37), 462(18), 343(4) | TSZ | 9 |
| 288 | Hydroxyjasmonic acid-O-sulphate | C_12_H_18_O_7_S | 8.65 | 305.0691 | 305.069 | 0.557 | 225(100), 261(55), 97(48), 287(34) | AY | 1, 2, 3 |
| 289 | 3-(3,4-Dihydroxyphenyl)propionic acid | C_9_H_10_O_4_ | 8.75 | 181.0496 | 181.0495 | 0.137 | 137(100), 163(41), 153(2), 121(2) | DZ | 1 |
| 290 | Medioresinol vanillic acid ether diglucopyranoside isomer | C_41_H_50_O_20_ | 8.9 | 861.2822 | 861.2812 | 1.161 | 843(100), 581(32), 337(25), 697(21) | / | 6, 7, 8 |
| 291 | Quinic acid isomer | C_7_H_12_O_6_ | 8.95 | 191.055 | 191.055 | 0.029 | 127(100), 85(87), 173(66), 93(53) | / | 3 |
| 292 | Arbutin | C_12_H_16_O_7_ | 9.02 | 271.0806 | 271.0812 | -2.321 | 227(100), 197(68), 153(52), 253(18) | TSZ | 1 |
| 293 | Scandoside methyl ester | C_17_H_24_O_11_ | 9.07 | 403.1223 | 403.1235 | -2.996 | 359(100), 179(16), 197(7), 223(5) | DZ | 4 |
| 294 | Eriodictyol chalcone | C_23_H_30_O_14_ | 9.1 | 529.154 | 529.1552 | -2.252 | 511(100), 367(29), 179(20), 211(16) | AY | 9 |
| 295 | Vanillin isomer | C_8_H_8_O_3_ | 9.16 | 151.0393 | 151.039 | 2.247 | 109(100), 108(49), 151(43), 123(41) | / | 1 |
| 296 | Medioresinol vanillic acid ether diglucopyranoside isomer | C_41_H_50_O_20_ | 9.24 | 861.2825 | 861.2812 | 1.509 | 843(100), 581(24), 701(18), 337(17) | / | 6, 7, 8 |
| 297 | Asperulosidic acid ethyl ester/ 3,4-dihydro-3β-ethoxyasperuloside | C_20_H_28_O_12_ | 9.25 | 459.1479 | 459.1497 | -3.948 | 161(100), 179(38), 281(32), 313(13) | DZ | 5 |
| 298 | 4-[2-(xylopyranosyloxy)ethyl]phenyl-xylopyranoside | C_18_H_26_O_10_ | 9.3 | 401.1429 | 401.1442 | -3.424 | 269(100), 161(25), 239(21), 357(9) | DZ | 3, 4, 5 |
| 299 | p-coumaric acid | C_9_H_8_O_3_ | 9.59 | 163.0392 | 163.039 | 1.53 | 119(100) | TSZ RS DZ | 1 |
| 300 | Hydroxyjasmonic acid hexose | C_18_H_28_O_9_ | 9.6 | 387.164 | 387.165 | -2.554 | 207(100), 163(57), 369(22), 225(7) | AY | 3 |
| 301 | Purpureaside C/Echinacoside | C_35_H_46_O_20_ | 9.63 | 785.2475 | 785.2499 | -3.018 | 623(100), 767(2), 605(2), 477(2) | SDH | 9, 10 |
| 302 | Cuscutoside C isomer | C_26_H_28_O_12_ | 9.64 | 531.1475 | 531.1497 | -4.222 | 369(100), 431(8), 270(8), 513(6) | / | 4, 5 |
| 303 | Isoschaftoside isomer | C_26_H_28_O_14_ | 9.64 | 563.1376 | 563.1395 | -3.377 | 473(100), 503(61), 383(56), 353(56) | / | 6 |
| 304 | Deacetyl asperulosidic acid methyl ester | C_17_H_24_O_11_ | 9.81 | 403.1219 | 403.1235 | -3.964 | 357(100), 179(50), 195(47), 385(44) | DZ BJT | 1-6 |
| 305 | (7R, 8S, 7'R, 8'S)-prinsepiol-4-O-β-D-glucopyranoside | C_26_H_32_O_13_ | 9.82 | 551.1738 | 551.1759 | -3.787 | 389(100), 193(35), 341(18), 515(8) | XD | 4 |
| 306 | 3-(3-hydroxyphenyl) propionic acid isomer | C_9_H_10_O_3_ | 9.87 | 165.0548 | 165.0546 | 0.965 | 123(100), 150(28), 165(14), 121(13) | / | 1 |
| 307 | (+)-pinoresinol di-O-β-D-glucopyranoside isomer | C_32_H_42_O_16_ | 9.87 | 681.2365 | 681.2389 | -3.613 | 519(100), 357(54), 518(50), 662(8) | / | 7 |
| 308 | 3-O-feruloylquinic acid/ 5-Feruoyl quinic acid | C_17_H_20_O_9_ | 9.89 | 367.1013 | 367.1024 | -2.911 | 191(100), 173(36), 193(8), 323(2) | AY DZ DS | 2 |
| 309 | Isoacteoside/Acteoside/Forsythoside A | C_26_H_40_O_17_ | 9.98 | 623.2161 | 623.2182 | -3.283 | 577(100), 579(11), 461(7), 605(6) | SDH | 10 |
| 310 | Morinlongosides C isomer | C_22_H_32_O_15_ | 10 | 535.1639 | 535.1657 | -3.488 | 489(100), 373(24), 355(9), 293(7) | BJT | 5 |
| 311 | Medioresinol vanillic acid ether diglucopyranoside isomer | C_41_H_50_O_20_ | 10.01 | 861.2833 | 861.2812 | 2.508 | 843(100), 697(25), 641(19), 539(14) | / | 6, 8 |
| 312 | (-)-olivil-4'-O-β-D-glucopyranoside | C_26_H_34_O_12_ | 10.03 | 537.1949 | 537.1967 | -3.281 | 375(100), 345(41), 327(12), 493(8) | DZ | 4 |
| 313 | Loliolide | C_11_H_16_O_3_ | 10.09 | 195.1015 | 195.1016 | -0.414 | 151(100), 59(58), 177(57), 165(20) | DZ | 1 |
| 314 | Glucosyringic acid isomer | C_15_H_20_O_10_ | 10.11 | 359.0964 | 359.0973 | -2.404 | 315(100), 341(87), 341(44), 313(40) | / | 2 |
| 315 | 3-(3-hydroxyphenyl) propionic acid isomer | C_9_H_10_O_3_ | 10.13 | 165.0548 | 165.0546 | 1.147 | 147(100), 119(1), 103(1), 121(1) | / | 1 |
| 316 | Decaffeoyl-verbascoside isomer | C_20_H_30_O_12_ | 10.13 | 461.1639 | 461.1654 | -3.258 | 415(100), 269(1), 417(1), 375(1) | / | 4 |
| 317 | 6′-O-β-D-apiofuranosyl sweroside | C_21_H_30_O_13_ | 10.17 | 489.1589 | 489.1603 | -2.856 | 293(100), 471(67), 195(47), 233(47) | XD | 4 |
| 318 | Isorhamnetin-O-dihexoside | C_29_H_36_O_16_ | 10.18 | 639.1896 | 639.192 | -3.725 | 431(100), 621(13), 477(11), 593(10) | TSZ | 5 |
| 319 | Deacetylasperuloside isomer | C_16_H_20_O_10_ | 10.19 | 371.0962 | 371.0973 | -3 | 249(100), 231(7), 353(5), 191(4) | / | 4 |
| 320 | Morinlongosides C isomer | C_22_H_32_O_15_ | 10.21 | 535.1638 | 535.1657 | -3.6 | 489(100), 517(5), 373(4), 355(3) | BJT | 4 |
| 321 | Isoschaftoside | C_26_H_28_O_14_ | 10.22 | 563.1378 | 563.1395 | -3.146 | 473(100), 503(71), 383(65), 443(65) | AY | 6 |
| 322 | Genipin gentiobioside | C_23_H_34_O_15_ | 10.25 | 549.1792 | 549.1814 | -4 | 503(100), 387(20), 532(16), 505(13) | DS | 4 |
| 323 | (+)-cycloolivil | C_20_H_24_O_7_ | 10.33 | 375.1425 | 375.1438 | -3.491 | 360(100), 327(71), 345(33), 357(26) | DZ | 1 |
| 324 | Purpureaside C/Echinacoside | C_35_H_46_O_20_ | 10.36 | 785.2478 | 785.2499 | -2.636 | 623(100), 461(3), 767(2), 740(2) | SDH | 9, 10 |
| 325 | 3,4-dihydro-3β-ethoxydesacetylasperuloside | C_18_H_26_O_11_ | 10.38 | 417.1379 | 417.1391 | -3.088 | 255(100), 357(50), 213(24), 195(19) | DZ | 3 |
| 326 | Ferulic acid^*^ | C_10_H_10_O_4_ | 10.41 | 193.0496 | 193.0495 | 0.076 | 149(100), 123(58), 121(41), 175(23) | DZ DS BZ | 1, 2 |
| 327 | Coumaroyl-Tricaffeoylquinic Acid | C_37_H_44_O_21_ | 10.47 | 823.2258 | 823.2291 | -4.014 | 661(100), 660(17), 498(12) | TSZ | 10 |
| 328 | p-coumaric acid glucoside isomer | C_15_H_18_O_8_ | 10.48 | 325.0911 | 325.0918 | -2.258 | 163(100), 119(61), 161(48), 101(13) | / | 3 |
| 329 | Apigenin-C-hexaose-C-pentosideb isomer | C_26_H_28_O_14_ | 10.51 | 563.1376 | 563.1395 | -3.377 | 473(100), 443(100), 383(97), 353(87) | / | 6, 7 |
| 330 | Decaffeoyl-verbascoside isomer | C_20_H_30_O_12_ | 10.55 | 461.1638 | 461.1654 | -3.323 | 415(100), 443(2), 299(1), 191(1) | / | 5 |
| 331 | (+)-pinoresinol di-O-β-D-glucopyranoside^*^ | C_32_H_42_O_16_ | 10.58 | 681.2362 | 681.2389 | -3.965 | 519(100), 357(31), 518(16), 635(13) | DZ | 7 |
| 332 | Coumaroyl-Tricaffeoylquinic Acid isomer | C_37_H_44_O_21_ | 10.58 | 823.2252 | 823.2291 | -4.828 | 487(100), 649(65), 662(57), 661(34) | / | 10 |
| 333 | (7R, 8S, 7'R, 8'S)-prinsepiol-4-O-β-D-glucopyranoside isomer | C_26_H_32_O_13_ | 10.6 | 551.1737 | 551.1759 | -4.005 | 389(100), 507(14), 193(13) | / | 4 |
| 334 | Casticin isomer | C_19_H_18_O_8_ | 10.63 | 373.0907 | 373.0918 | -2.932 | 355(100), 343(22), 301(14), 329(10) | AY | 2 |
| 335 | Naringin SDHC 4-O-β-d-glucopyranoside | C_33_H_44_O_19_ | 10.72 | 743.2354 | 743.2393 | -5.322 | 697(100), 699(66), 581(53), 726(35) | DZ | 6, 8 |
| 336 | Apigenin-C-hexaose-C-pentosideb | C_26_H_28_O_14_ | 10.77 | 563.1373 | 563.1395 | -4.016 | 473(100), 503(80), 353(57), 383(47) | AY | 5, 7 |
| 337 | Eucomoside C | C_27_H_32_N_2_O_11_ | 10.77 | 559.1901 | 559.1922 | -3.784 | 379(100), 335(78), 361(32), 291(31) | DZ | 10 |
| 338 | Decaffeoyl-verbascoside | C_20_H_30_O_12_ | 10.81 | 461.1639 | 461.1654 | -3.193 | 415(100), 443(9), 417(5), 191(4) | SDH | 3, 4 |
| 339 | Eucommioside II | C_15_H_26_O_9_ | 10.85 | 349.1484 | 349.1493 | -2.574 | 187(100), 259(7), 305(7), 161(7) | DZ | 2 |
| 340 | Cantleyine | C_11_H_13_NO_3_ | 10.87 | 206.0812 | 206.0812 | -0.096 | 164(100) | XD | 1 |
| 341 | Methyl chlorogenate | C_17_H_20_O_9_ | 10.94 | 367.1013 | 367.1024 | -2.911 | 191(100), 179(56), 135(20), 161(6) | DZ | 2 |
| 342 | 3-[4-(2-[4-glucopyranosyloxy-3-methoxyphenyl)-2-hydroxy-1-(hydroxymethyl)ethoxy]-3,5−dimethoxyphenyl]-2-propen-1-ylglucopyranoside | C_33_H_46_O_18_ | 10.96 | 729.2568 | 729.26 | -4.389 | 683(100), 521(45), 567(26), 711(26) | DZ | 8 |
| 343 | 3-(3-hydroxyphenyl) propionic acid | C_9_H_10_O_3_ | 11.01 | 165.0549 | 165.0546 | 1.45 | 109(100), 136(85), 121(64), 165(54) | DZ | 1 |
| 344 | 4,8,9,10-tetrahydroxy-3,6,7-trimethoxy-2-anthryl-glucopyranoside | C_23_H_26_O_13_ | 11.03 | 509.1273 | 509.129 | -3.353 | 297(100), 311(42), 197(10), 491(6) | DZ | 2 |
| 345 | Purpureaside C/Echinacoside | C_35_H_46_O_20_ | 11.07 | 785.2468 | 785.2499 | -3.948 | 623(100), 461(3), 605(2), 741(1) | SDH | 9, 10 |
| 346 | l-hydroxypinoresinol 4'-di-O-glucopyranoside | C_26_H_32_O_12_ | 11.09 | 535.1794 | 535.181 | -3.032 | 373(100), 343(6), 517(5), 491(5) | DZ | 3 |
| 347 | Ulmoside | C_21_H_32_O_14_ | 11.11 | 507.1692 | 507.1708 | -3.178 | 463(100), 489(83), 461(64), 488(58) | DZ | 2 |
| 348 | Vitexin | C_21_H_20_O_10_ | 11.24 | 431.096 | 431.0973 | -3 | 311(100), 341(14), 269(18) | AY | 4 |
| 349 | Prunin isomer | C_21_H_22_O_10_ | 11.25 | 433.1116 | 433.1129 | -3.055 | 323(100), 161(59), 221(17), 312(15) | / | 3 |
| 350 | Thunberginol C | C_15_H_12_O_5_ | 11.29 | 271.0598 | 271.0601 | -1.291 | 253(100), 256(41), 215(31), 243(30) | DZ | 1 |
| 351 | Citrusin B isomer | C_27_H_36_O_13_ | 11.3 | 567.2053 | 567.2072 | -3.345 | 521(100), 549(98), 405(84), 523(43) | / | 4 |
| 352 | Cuscutoside B | C_31_H_36_O_16_ | 11.34 | 663.1892 | 663.192 | -4.239 | 501(100), 337(3), 307(2), 543(1) | TSZ | 5, 6 |
| 353 | 7-Deoxyloganic acid | C_16_H_24_O_9_ | 11.35 | 359.1327 | 359.1337 | -2.614 | 197(100), 153(22), 135(14), 109(6) | XD | 3, 6 |
| 354 | Eucommioside I | C_15_H_26_O_9_ | 11.36 | 349.1484 | 349.1493 | -2.488 | 187(100), 305(8), 331(5), 161(5) | DZ | 2, 3 |
| 355 | 3-(3-hydroxyphenyl) propionic acid isomer | C_9_H_10_O_3_ | 11.39 | 165.0548 | 165.0546 | 1.329 | 123(100), 121(80), 150(65), 165(44) | / | 1 |
| 356 | Kaempferol‑3‑β‑D‑glucuronide/ Luteolin−7-glycuronic acid | C_21_H_18_O_12_ | 11.47 | 461.0702 | 461.0715 | -2.803 | 285(100), 415(5), 357(4), 327(3) | TSZ AY | 4 |
| 357 | 3-[4-(2-[4-glucopyranosyloxy-3-methoxyphenyl)-2-hydroxy-1-(hydroxymethyl)ethoxy]-3,5-dimethoxyphenyl]-2-propen-1-ylglucopyranoside isomer | C_33_H_46_O_18_ | 11.48 | 729.2581 | 729.26 | -2.634 | 683(100), 710(47), 684(37), 711(33) | / | 7 |
| 358 | 6β-hydroxyl-1β,3β-dimethoxyartsclaenin III isomer | C_11_H_18_O_5_ | 11.49 | 229.107 | 229.1071 | -0.437 | 211(100), 99(15), 167(13), 193(9) | / | 1 |
| 359 | 3-glucopyranosyloxy-5,7-dihydroxy-2-(4-hydroxy-3-methoxyphenyl)-4H-1-benzopyran-4-one | C_22_H_22_O_12_ | 11.68 | 477.1013 | 477.1028 | -3.065 | 431(100), 269(36), 459(6), 433(4) | DZ | 2 |
| 360 | Citrusin B | C_27_H_36_O_13_ | 11.69 | 567.2053 | 567.2072 | -3.451 | 549(100), 523(65), 405(64), 521(61) | DZ | 3 |
| 361 | Hexyl-β-D-glucopyranosyl-(1→2)-β-D-glucopyranoside | C_18_H_34_O_11_ | 11.73 | 425.2005 | 425.2017 | -2.983 | 263(100), 161(23), 159(4), 143(3) | DS | 5 |
| 362 | Epieucommiol | C_10_H_18_O_5_ | 11.76 | 217.107 | 217.1071 | -0.415 | 155(100), 171(17), 199(16), 137(10) | DZ | 1, 2 |
| 363 | 4-[[6-O-(4-hydroxy-3,5-dimethoxybenzoyl)-glucopyranosyl]oxy]-3-methoxybenzoic acid | C_23_H_26_O_13_ | 11.78 | 509.1272 | 509.129 | -3.53 | 327(100), 341(40), 183(8), 491(4) | DZ | 2 |
| 364 | Acanthoside D | C_34_H_46_O_18_ | 11.86 | 741.257 | 741.26 | -4.075 | 323(100), 723(78), 695(71), 579(35) | DZ | 7 |
| 365 | (-)-Epicatechin isomer | C_15_H_14_O_6_ | 11.98 | 289.07 | 289.0707 | -2.472 | 271(100), 245(57), 177(47), 161(24) | / | 1 |
| 366 | Eucomoside B | C_25_H_31_NO_11_ | 12.01 | 520.1793 | 520.1813 | -3.878 | 234(100), 164(67), 190(65), 355(61) | DZ | 5, 6 |
| 367 | Hedyotol C di-O-glucopyranoside | C_43_H_56_O_21_ | 12.01 | 907.3197 | 907.323 | -3.675 | 745(100), 890(84), 805(69), 885(30) | DZ | 8 |
| 368 | Isorhamnetin-3-O-glucoside isomer | C_22_H_22_O_12_ | 12.08 | 477.1011 | 477.1028 | -3.526 | 315(100), 300(14), 357(4), 431(3) | / | 4 |
| 369 | 3-[4-(2-[4-glucopyranosyloxy-3-methoxyphenyl)-2-hydroxy-1-(hydroxymethyl)ethoxy]-3,5-dimethoxyphenyl]-2-propen-1-ylglucopyranoside isomer | C_33_H_46_O_18_ | 12.1 | 729.2569 | 729.26 | -4.307 | 682(100), 684(85), 566(81), 567(78) | / | 7 |
| 370 | Jionoside B1/Jionoside B2 | C_37_H_50_O_20_ | 12.11 | 813.2781 | 813.2812 | -3.8 | 637(100), 619(23), 651(6), 491(6) | SDH | 6 |
| 371 | Cuscutoside C isomer | C_26_H_28_O_12_ | 12.12 | 531.1488 | 531.1497 | -1.699 | 369(100), 431(97), 269(15) | / | 4 |
| 372 | 6β-hydroxyl-1β,3β-dimethoxyartsclaenin III | C_11_H_18_O_5_ | 12.18 | 229.1069 | 229.1071 | -0.655 | 211(100), 99(15), 167(13), 193(7) | DZ | 1 |
| 373 | C-veratroylglycol | C_10_H_12_O_5_ | 12.18 | 211.0601 | 211.0601 | -0.047 | 139(100), 149(40), 167(35), 123(20) | DZ | 1 |
| 374 | Asperulosidic acid | C_18_H_24_O_12_ | 12.25 | 431.1169 | 431.1184 | -3.462 | 413(100), 357(99), 387(94), 387(61) | DZ | 2 |
| 375 | 1-hydroxypinoresinol 4',4''-di-O-β-D-glucopyranoside isomer | C_32_H_42_O_17_ | 12.27 | 697.2306 | 697.2338 | -4.641 | 517(100), 535(91), 679(82), 571(45) | / | 4 |
| 376 | 3-O-caffeoyl-5-O-(3'-O-caffeoyl glucosyl)quinic acid | C_31_H_34_O_17_ | 12.29 | 677.1683 | 677.1712 | -4.276 | 505(100), 353(66), 687(62), 514(38) | DZ | 4, 5 |
| 377 | Dipsalignan B isomer | C_20_H_22_O_8_ | 12.3 | 389.1218 | 389.1231 | -3.248 | 195(100), 193(32), 371(14), 374(13) | / | 1 |
| 378 | Isorhamnetin-3-O-glucoside | C_22_H_22_O_12_ | 12.3 | 477.101 | 477.1028 | -3.589 | 314(100), 315(59), 357(14), 285(8) | TSZ | 3 |
| 379 | Cantleyoside isomer | C_33_H_46_O_19_ | 12.32 | 745.2526 | 745.255 | -3.214 | 583(100), 459(12) | / | 6 |
| 380 | Apigenin-O-rutinoside | C_27_H_30_O_14_ | 12.33 | 577.153 | 577.1552 | -3.867 | 269(100) | AY | 4, 5 |
| 381 | Ulmoside D isomer | C_25_H_40_O_13_ | 12.34 | 547.2363 | 547.2385 | -3.997 | 311(100), 457(74), 487(40), 191(32) | / | 5 |
| 382 | Hedyotol C di-O-glucopyranoside | C_43_H_56_O_21_ | 12.38 | 907.32 | 907.323 | -3.334 | 611(100), 459(43), 745(39), 818(33) | DZ | 8 |
| 383 | Sylvestroside I | C_33_H_48_O_19_ | 12.41 | 747.2675 | 747.2706 | -4.209 | 585(100), 485(44), 459(23), 357(15) | XD | 7 |
| 384 | Jionoside B1/Jionoside B2 | C_37_H_50_O_20_ | 12.42 | 813.2776 | 813.2812 | -4.402 | 637(100), 619(22), 491(6), 473(3) | SDH | 6 |
| 385 | Dehydrodiconiferyl alcohol 4-γ'-di-O-β-D-glucopyranoside | C_32_H_42_O_16_ | 12.46 | 681.236 | 681.2389 | -4.244 | 323(10), 645(24), 663(12), 635(11) | DZ | 5 |
| 386 | Pinoresinol-4-O-glucoside | C_26_H_32_O_11_ | 12.47 | 519.1845 | 519.1861 | -3.04 | 357(100), 501(3), 475(2), 489(2) | TSZ DZ | 2, 3 |
| 387 | (7R, 8S, 7'R, 8'S)-fraxiresinol-4'-O-β-D-glucopyranoside | C_27_H_34_O_13_ | 12.47 | 565.1896 | 565.1916 | -3.41 | 521(100), 403(70), 547(70), 529(40) | XD | 2, 3 |
| 388 | ​Hexenyl D-​glucopyranoside | C_12_H_22_O_6_ | 12.52 | 261.1329 | 261.1333 | -1.282 | 187(100), 125(43), 169(6), 217(2) | DS | 1, 2 |
| 389 | (+)-Epipinoresinol/ Pinoresinol | C_20_H_22_O_6_ | 12.53 | 357.1324 | 357.1333 | -2.562 | 357(100) | TSZ DZ | 2, 3 |
| 390 | Acanthoside D isomer | C_34_H_46_O_18_ | 12.63 | 741.2565 | 741.26 | -4.736 | 323(100), 417(30), 469(15), 723(13) | / | 5 |
| 391 | Isomer of Emodin-O-glucoside | C_21_H_20_O_10_ | 12.64 | 431.0959 | 431.0973 | -3.208 | 269(100), 268(16), 311(3) | HSW | 3 |
| 392 | 1-hydroxypinoresinol 4',4''-di-O-β-D-glucopyranoside isomer | C_32_H_42_O_17_ | 12.65 | 697.2307 | 697.2338 | -4.469 | 535(100), 679(91), 678(63), 651(61) | / | 4 |
| 393 | Ulmoside D isomer | C_25_H_40_O_13_ | 12.69 | 547.2364 | 547.2385 | -3.888 | 311(100), 458(28), 293(25), 503(23) | / | 5 |
| 394 | Dipsanoside C/ Dipsanoside D/ Dipsanoside E/ Dipsanoside F | C_36_H_52_O_21_ | 12.77 | 819.2874 | 819.2917 | -5.279 | 657(100), 587(43), 495(41), 743(24) | XD | 7 |
| 395 | MeO-Cantleyoside | C_34_H_48_O_20_ | 12.8 | 775.2621 | 775.2655 | -3.44 | 581(100), 403(76), 595(40), 341(21) | XD | 7 |
| 396 | Ulmoside D | C_25_H_40_O_13_ | 12.82 | 547.2364 | 547.2385 | -3.888 | 311(100), 529(31), 503(24), 191(15) | DZ | 4 |
| 397 | Dipsalignan B | C_20_H_22_O_8_ | 12.84 | 389.1218 | 389.1231 | -3.326 | 195(100), 193(28), 371(18), 374(13) | XD | 1 |
| 398 | 2-hydroxy-1-hydroxymethylanthraquinone | C_17_H_14_O_6_ | 12.86 | 313.0697 | 313.0707 | -3.177 | 269(100), 203(11), 295(5), 267(4) | BJT | 1 |
| 399 | Syringaresinol-O-β-D-glucopyranoside | C_28_H_36_O_13_ | 12.87 | 579.2051 | 579.2072 | -3.69 | 417(100) | DZ | 2, 3 |
| 400 | 3-Octen-2-one | C_8_H_14_O | 12.96 | 125.0966 | 125.0961 | 3.824 | 97(100), 57(12), 80(6), 83(5) | SJS | 3 |
| 401 | Dipsanoside C/ Dipsanoside D/ Dipsanoside E/ Dipsanoside F | C_36_H_52_O_21_ | 12.96 | 819.2872 | 819.2917 | -5.498 | 657(100), 587(27), 495(26), 776(11) | XD | 6 |
| 402 | (7R, 8S, 7'R, 8'S)-fraxiresinol-4'-O-β-D-glucopyranoside isomer | C_27_H_34_O_13_ | 12.97 | 565.1894 | 565.1916 | -3.852 | 357(100), 519(22) | / | 2 |
| 403 | Daphylloside | C_19_H_26_O_12_ | 13.13 | 445.1326 | 445.1341 | -3.196 | 401(100), 399(80), 427(57), 195(46) | DZ | 1 |
| 404 | Dipsanoside C/ Dipsanoside D/ Dipsanoside E/ Dipsanoside F | C_36_H_52_O_21_ | 13.19 | 819.2885 | 819.2917 | -4.009 | 657(100), 495(50), 587(48), 459(22) | XD | 7 |
| 405 | Medioresinol vanillic acid ether diglucopyranoside | C_41_H_50_O_20_ | 13.2 | 861.2772 | 861.2812 | -4.656 | 699(100), 341(23), 311(7), 357(3) | DZ | 5 |
| 406 | Pinoresinol vanillic acid ether diglucopyranoside | C_40_H_48_O_19_ | 13.28 | 831.2677 | 831.2706 | -3.495 | 669(100), 311(13), 343(6), 813(6) | DZ | 5 |
| 407 | 3,4-dihydro-3β-ethoxydesacetylasperuloside isomer | C_18_H_26_O_11_ | 13.33 | 417.1378 | 417.1391 | -3.303 | 209(100), 371(36), 399(25), 373(21) | / | 2 |
| 408 | Syringaresinol vanillic acid ether diglucopyranoside | C_42_H_52_O_21_ | 13.39 | 891.2875 | 891.2917 | -4.706 | 729(100), 566(5), 311(4), 403(3) | DZ | 5 |
| 409 | Cantleyoside | C_33_H_46_O_19_ | 13.43 | 745.2518 | 745.255 | -4.274 | 513(100), 583(65), 459(33), 565(14) | XD | 5, 6 |
| 410 | 1F-fructofuranosylnystose/ Inulopentaose/ 1F-Fructofuranosylnystose | C_30_H_52_O_26_ | 13.45 | 827.2716 | 827.2663 | 6.409 | 665(100), 809(4), 765(2), 809(2) | BJT | 5 |
| 411 | 5-hydroxy-4′,6,7-trimethoxy flavone | C_18_H_16_O_6_ | 13.49 | 327.0855 | 327.0863 | -2.643 | 312(100), 283(22), 297(13), 309(11) | DS | 1 |
| 412 | Kaempferol 3-O-rhamnoside | C_21_H_20_O_10_ | 13.51 | 431.0958 | 431.0973 | -3.348 | 285(100), 269(17), 284(17) | RS | 2 |
| 413 | Biatractylenolide Ⅱisomer | C_34_H_42_O_8_ | 13.62 | 577.2832 | 577.2796 | 6.245 | 531(100), 415(3), 400(1), 559(1) | / | 5 |
| 414 | Dipsalignan A/ 8-hydroxy-medioresinol | C_21_H_24_O_8_ | 13.63 | 403.1378 | 403.1387 | -2.367 | 343(100), 373(58), 388(9), 355(6) | XD DZ | 1 |
| 415 | Syringaresinol vanillic acid ether diglucopyranoside isomer | C_42_H_52_O_21_ | 13.74 | 891.2886 | 891.2917 | -3.472 | 729(100), 311(6), 874(6), 609(3) | / | 5 |
| 416 | 1F-fructofuranosylnystose/ Inulopentaose/ 1F-Fructofuranosylnystose | C_30_H_52_O_26_ | 13.89 | 827.2712 | 827.2663 | 5.89 | 665(100), 809(14), 808(9), 783(9) | BJT | 4, 5 |
| 417 | Macranthoin G | C_26_H_26_O_12_ | 13.93 | 529.1321 | 529.1341 | -3.728 | 367(100), 161(7), 353(6), 511(2) | DZ | 2 |
| 418 | Jionoside B1/Jionoside B2 | C_37_H_50_O_20_ | 14.02 | 813.2771 | 813.2812 | -5.005 | 652(100), 769(75), 650(64), 632(58) | SDH | 5, 7 |
| 419 | Syringin | C_17_H_24_O_9_ | 14.05 | 371.1325 | 371.1337 | -3.014 | 327(100), 137(56), 353(30), 325(28) | DZ DS BZ | 2 |
| 420 | Cuscutoside C | C_26_H_28_O_12_ | 14.27 | 531.1476 | 531.1497 | -3.996 | 351(100), 339(78), 191(27), 215(19) | TSZ | 2 |
| 421 | 1F-fructofuranosylnystose/ Inulopentaose/ 1F-Fructofuranosylnystose | C_30_H_52_O_26_ | 14.31 | 827.2714 | 827.2663 | 6.107 | 665(100), 488(17), 487(9), 307(8) | BJT | 5 |
| 422 | 1-deoxyeucommiol | C_9_H_16_O_3_ | 14.41 | 171.1017 | 171.1016 | 0.755 | 153(100), 127(86), 125(62), 97(7) | DZ | 1 |
| 423 | 1F-fructofuranosylnystose/ Inulopentaose/ 1F-Fructofuranosylnystose | C_30_H_52_O_26_ | 14.48 | 827.271 | 827.2663 | 5.672 | 665(100), 809(4), 664(3), 765(2) | BJT | 4 |
| 424 | Dipsanoside C/ Dipsanoside D/ Dipsanoside E/ Dipsanoside F | C_36_H_52_O_21_ | 14.54 | 819.2885 | 819.2917 | -4.009 | 656(100), 657(67), 732(65), 775(42) | XD | 5 |
| 425 | Glycerol-syringaresinol ether 4''-glucopyranoside | C_38_H_48_O_17_ | 14.61 | 775.277 | 775.2808 | -4.819 | 613(100), 565(12), 417(7), 595(4) | DZ | 3 |
| 426 | Methyl 3,5-dicaffeoylquinate | C_26_H_26_O_12_ | 14.62 | 529.1321 | 529.1341 | -3.615 | 367(100), 353(10), 173(9), 335(8) | XD | 2, 9 |
| 427 | Glycerol-medioresinol ether 4''-glucopyranoside | C_37_H_46_O_16_ | 14.7 | 745.2669 | 745.2702 | -4.43 | 583(100), 535(11), 387(6), 565(2) | DZ | 3 |
| 428 | 1F-fructofuranosylnystose/ Inulopentaose/ 1F-Fructofuranosylnystose | C_30_H_52_O_26_ | 14.82 | 827.2709 | 827.2663 | 5.515 | 665(100), 809(13), 767(9), 797(6) | BJT | 4 |
| 429 | 4-Feruloyl-5-caffeoylquinicacid | C_26_H_26_O_12_ | 14.92 | 529.1319 | 529.1341 | -4.068 | 353(100), 367(36), 173(7), 203(6) | TSZ | 2 |
| 430 | Cuscutoside D | C_37_H_46_O_21_ | 14.99 | 825.2396 | 825.2448 | -6.258 | 369(100), 663(73), 323(11) | TSZ | 8 |
| 431 | Codonopsinol C | C_12_H_17_NO_4_ | 15.03 | 238.1072 | 238.1074 | -0.733 | 164(100), 152(41), 194(15), 124(13) | DS | 1 |
| 432 | Tangshenoside VI | C_39_H_50_O_19_ | 15.03 | 821.2822 | 821.2863 | -4.975 | 777(100), 804(81), 775(70), 790(62) | DS | 3 |
| 433 | Lobetyolinin isomer | C_26_H_38_O_13_ | 15.03 | 557.2209 | 557.2229 | -3.549 | 203(100), 395(22), 511(20), 515(11) | / | 3 |
| 434 | 1F-fructofuranosylnystose/ Inulopentaose/ 1F-Fructofuranosylnystose | C_30_H_52_O_26_ | 15.07 | 827.2712 | 827.2663 | 5.962 | 665(100), 664(7), 502(4), 501(3) | BJT | 4 |
| 435 | Glycerol-syringaresinol ether 4'''-glucopyranoside | C_38_H_48_O_17_ | 15.33 | 775.277 | 775.2808 | -4.897 | 613(100), 417(8), 565(6), 387(3) | DZ | 3 |
| 436 | Martynoside | C_31_H_40_O_15_ | 15.36 | 651.2259 | 651.2283 | -3.773 | 475(100), 505(30), 457(23), 633(13) | SDH | 2, 3 |
| 437 | d-Sesamin | C_20_H_18_O_6_ | 15.39 | 353.101 | 353.102 | -2.704 | 338(100), 294(13), 309(12), 322(8) | TSZ | 1 |
| 438 | 1-hydroxypinoresinol 4',4''-di-O-β-D-glucopyranoside | C_32_H_42_O_17_ | 15.43 | 697.2306 | 697.2338 | -4.641 | 679(100), 661(36), 652(30), 651(29) | DZ | 2, 3 |
| 439 | 5-hydroxy-4′,6,7-trimethoxy flavone isomer | C_18_H_16_O_6_ | 15.51 | 327.0856 | 327.0863 | -2.093 | 283(100), 309(6), 147(4), 312(3) | / | 1 |
| 440 | Glycerol-medioresinol ether 4'''-glucopyranoside | C_37_H_46_O_16_ | 15.57 | 745.2667 | 745.2702 | -4.765 | 583(100), 535(16), 565(4), 709(4) | DZ | 3 |
| 441 | Lobetyolinin | C_26_H_38_O_13_ | 15.67 | 557.2205 | 557.2229 | -4.213 | 395(100), 511(57), 513(40), 521(39) | DS | 2 |
| 442 | Atractyloside A isomer | C_21_H_36_O_10_ | 15.73 | 447.2209 | 447.2225 | -3.541 | 315(100), 161(20), 131(3), 397(2) | / | 4 |
| 443 | Apigenin-7-O-β-D-glucopyranoside | C_21_H_20_O_10_ | 15.79 | 431.0958 | 431.0973 | -3.44 | 369(100), 287(88), 269(73), 413(26) | DS | 1 |
| 444 | 3-Caffeoyl-4-feruoyl-quinic acid | C_26_H_26_O_12_ | 15.91 | 529.132 | 529.1341 | -3.841 | 367(100), 179(8), 349(5), 161(4) | AY | 2 |
| 445 | E-2-Nonenal | C_9_H_16_O | 15.93 | 139.1121 | 139.1117 | 2.72 | 57(100), 111(78), 69(23), 95(21) | SJS | 1 |
| 446 | Prunin | C_21_H_22_O_10_ | 15.99 | 433.1115 | 433.1129 | -3.402 | 271(100) | DZ | 2 |
| 447 | Neokurarinol isomer | C_27_H_34_O_7_ | 16.14 | 469.2262 | 469.2221 | 8.845 | 423(100) | / | 4 |
| 448 | Atractyloside A | C_21_H_36_O_10_ | 16.22 | 447.221 | 447.2225 | -3.34 | 315(100), 161(18), 159(2), 301(2) | BZ | 3 |
| 449 | 6-(3-hydroxy-propionyloxy)atractylenolid III | C_18_H_22_O_6_ | 16.42 | 333.1324 | 333.1333 | -2.746 | 261(100), 305(26), 203(4), 189(2) | BZ | 2 |
| 450 | Ethylsyringin | C_19_H_28_O_9_ | 16.44 | 399.1635 | 399.165 | -3.705 | 261(100), 353(29), 355(25), 384(23) | DS | 1 |
| 451 | Martynoside isomer | C_31_H_40_O_15_ | 16.5 | 651.2253 | 651.2283 | -4.709 | 475(100), 505(91), 265(22), 329(19) | SDH | 2, 3 |
| 452 | Alternariol | C_14_H_10_O_5_ | 16.62 | 257.0441 | 257.0444 | -1.244 | 149(100), 227(62), 239(10), 213(10) | DZ | 1 |
| 453 | Wogonoside isomer | C_22_H_20_O_11_ | 16.71 | 459.0909 | 459.0922 | -2.849 | 283(100), 413(26), 175(18), 421(7) | / | 3 |
| 454 | 3,4,5-Tricaffeoylquinic acid | C_34_H_30_O_15_ | 16.73 | 677.1472 | 677.1501 | -4.337 | 515(100), 353(8), 497(3), 659(1) | TSZ | 3 |
| 455 | Neokurarinol | C_27_H_34_O_7_ | 16.93 | 469.2258 | 469.2221 | 7.864 | 423(100), 451(1) | DS | 4 |
| 456 | Syringaresinol vanillic acid ether glucopyranoside | C_36_H_42_O_16_ | 17.03 | 729.2356 | 729.2389 | -4.541 | 311(100), 341(49), 403(28), 373(20) | DZ | 2 |
| 457 | Asperuloside tetraacetate | C_26_H_30_O_15_ | 17.06 | 581.1473 | 581.1501 | -4.846 | 443(100), 461(19), 499(11), 269(11) | BJT | 3 |
| 458 | Medioresinol vanillic acid ether glucopyranoside | C_35_H_40_O_15_ | 17.13 | 699.225 | 699.2283 | -4.729 | 341(100), 343(7), 327(4), 311(4) | DZ | 2 |
| 459 | Isomer of Emodin-O-glucoside | C_21_H_20_O_10_ | 17.19 | 431.0957 | 431.0973 | -3.719 | 269(100), 311(8), 293(5), 413(2) | HSW | 2, 3 |
| 460 | Pinoresinol vanillic acid ether glucopyranoside | C_34_H_38_O_14_ | 17.41 | 669.215 | 669.2178 | -4.112 | 311(100), 343(12), 327(6), 639(3) | DZ | 2 |
| 461 | Naringenin | C_15_H_12_O_5_ | 17.42 | 271.0596 | 271.0601 | -1.734 | 151(100), 177(21), 227(8), 253(6) | DZ | 1 |
| 462 | Biatractylenolide Ⅱ isomer | C_34_H_42_O_8_ | 17.67 | 577.2826 | 577.2796 | 5.189 | 531(100), 560(2), 489(2), 533(2) | / | 4 |
| 463 | l-hydroxypinoresinol 4''-di-O-glucopyranoside | C_26_H_32_O_12_ | 17.69 | 535.1789 | 535.181 | -3.948 | 373(100), 173(3), 199(2), 489(2) | DZ | 2 |
| 464 | Wogonoside | C_22_H_20_O_11_ | 17.74 | 459.0906 | 459.0922 | -3.437 | 283(100), 175(16), 413(6), 268(6) | DZ | 2, 3 |
| 465 | Ginsenoside 20-Glc-Rf/ Ginsenoside Re1/ Ginsenoside Re2/ Ginsenoside Re3/ Notoginsenoside N | C_48_H_82_O_19_ | 17.9 | 961.5331 | 961.5367 | -3.72 | 915(100), 747(50), 869(35), 559(35) | RS | 7 |
| 466 | d-Sesamin isomer | C_20_H_18_O_6_ | 18.01 | 353.1008 | 353.102 | -3.383 | 338(100), 309(2), 229(2), 335(1) | / | 1 |
| 467 | Biatractylenolide Ⅱ | C_34_H_42_O_8_ | 18.04 | 577.2823 | 577.2796 | 4.669 | 531(100), 534(7), 509(6), 399(4) | BZ | 4 |
| 468 | cis/trans-Linalool oxide | C_10_H_18_O_2_ | 18.13 | 169.1225 | 169.1223 | 0.85 | 151(100), 169(57), 125(29) | SJS | 1 |
| 469 | Cuscutoside A | C_31_H_36_O_16_ | 18.3 | 663.1889 | 663.192 | -4.616 | 369(100), 500(3), 516(2), 645(1) | TSZ | 3 |
| 470 | Hedyotol C/ Hedyotol D | C_31_H_36_O_11_ | 18.7 | 583.2156 | 583.2174 | -2.998 | 535(100), 387(24), 357(16), 553(12) | DZ | 1 |
| 471 | Ginsenoside 20-Glc-Rf/ Ginsenoside Re1/ Ginsenoside Re2/ Ginsenoside Re3/ Notoginsenoside N | C_48_H_82_O_19_ | 18.95 | 961.5325 | 961.5367 | -4.292 | 916(100), 917(14), 827(11), 436(10) | RS | 8 |
| 472 | 1-hydroxy-anthraquinone | C_14_H_8_O_3_ | 19.01 | 223.0388 | 223.039 | -0.81 | 179(100), 223(93), 205(93), 195(61) | BJT | 1 |
| 473 | Hexyl-β-D-glucopyranosyl-(1→6)-β-D-glucopyranoside | C_18_H_34_O_11_ | 19.12 | 425.2 | 425.2017 | -4.064 | 379(100), 381(25), 407(9), 335(6) | DS | 2 |
| 474 | Apigenin-O-rutinoside isomer | C_27_H_30_O_14_ | 19.44 | 577.1528 | 577.1552 | -4.075 | 369(100), 531(6) | / | 2 |
| 475 | Medioresinol | C_21_H_24_O_7_ | 19.56 | 387.1427 | 387.1438 | -2.84 | 161(100), 133(9), 369(6), 343(4) | DZ | 1 |
| 476 | Hedyotol C/ Hedyotol D | C_31_H_36_O_11_ | 19.77 | 583.215 | 583.2174 | -4.044 | 535(100), 387(73), 357(42), 373(20) | DZ | 1 |
| 477 | 6-(3-hydroxy-propionyloxy)atractylenolid III isomer | C_18_H_22_O_6_ | 20.09 | 333.1323 | 333.1333 | -3.016 | 289(100), 245(64), 227(20), 271(17) | / | 1 |
| 478 | 9,12,13-trihydroxy-10,15-octadecadienoic acid | C_18_H_32_O_5_ | 20.59 | 327.2157 | 327.2166 | -2.752 | 171(100), 229(44), 291(41), 211(27) | DS | 1, 2 |
| 479 | Lancemaside B/ Lancemaside E/ Codonolaside III | C_63_H_100_O_31_ | 20.64 | 1351.6116 | 1351.6165 | -3.634 | 912(100), 911(30), 1307(15), 1333(10) | DS | 11 |
| 480 | 9,12,13-Trihydroxy octadecadienoic acid | C_18_H_32_O_5_ | 20.81 | 327.2157 | 327.2166 | -2.66 | 229(100), 211(61), 291(53), 171(44) | AY | 1, 2 |
| 481 | Codonopsinol B | C_13_H_19_NO_4_ | 20.92 | 252.1227 | 252.123 | -1.287 | 179(100), 193(52), 208(13), 150(12) | DS | 1 |
| 482 | Emodin-(acetyl)-O-glucoside | C_23_H_22_O_11_ | 21.05 | 473.1061 | 473.1078 | -3.652 | 269(100), 311(8), 293(5), 25(3) | HSW | 2 |
| 483 | Apigenin-O-rutinoside isomer | C_27_H_30_O_14_ | 21.09 | 577.1556 | 577.1552 | 0.69 | 541(100) | / | 4, 5 |
| 484 | Codonopsinol B isomer | C_13_H_19_NO_4_ | 21.44 | 252.1222 | 252.123 | -3.151 | 193(100), 208(21), 234(5), 225(5) | / | 1 |
| 485 | 1-hydroxy-anthraquinone isomer | C_14_H_8_O_3_ | 21.63 | 223.0388 | 223.039 | -0.765 | 179(100), 178(92), 223(39), 195(36) | BJT | 1 |
| 486 | Diethyl phthalate | C_12_H_14_O_4_ | 21.92 | 221.0807 | 221.0808 | -0.793 | 149(100), 177(69), 71(43), 147(32) | XD | 15, 16 |
| 487 | 12-senecioyloxytetradeca-2E,8E,10Etrien-4,6-diyne-1,14-diacetate | C_23_H_26_O_6_ | 22.09 | 397.166 | 397.1646 | 3.664 | 353(100), 369(8), 379(7), 172(4) | BZ | 2 |
| 488 | Anthraquinone-2-carboxylic acid | C_15_H_8_O_4_ | 22.21 | 251.0336 | 251.0339 | -1.017 | 207(100), 225(18) | BJT | 1 |
| 489 | Biatractylenolide Ⅱ isomer | C_34_H_42_O_8_ | 22.32 | 577.2827 | 577.2796 | 5.293 | 531(100), 415(3), 560(3), 423(2) | / | 3 |
| 490 | 5,7-Dihydroxy-8,2'-dimethoxyflavone | C_17_H_14_O_6_ | 22.45 | 313.0699 | 313.0707 | -2.602 | 298(100), 269(6), 283(2), 299(1) | AY | 1 |
| 491 | Malonyl-ginsenoside Ra3/ R4 | C_62_H_102_O_30_ | 22.66 | 1325.6326 | 1325.6372 | -3.506 | 1307(100), 1235(53), 954(35), 1120(33) | RS | 11 |
| 492 | 9,12,13-Trihydroxy octadecenoic acid isomer | C_18_H_34_O_5_ | 22.75 | 329.2314 | 329.2323 | -2.644 | 229(100), 211(66), 311(32), 293(31) | / | 1-4 |
| 493 | 5,6,9-trihydroxy-octadec-7-enoic acid | C_18_H_34_O_5_ | 23.01 | 329.2314 | 329.2323 | -2.735 | 229(100), 211(66), 311(33), 293(28) | DS | 1, 2, 3 |
| 494 | Eriojaposide B | C_25_H_40_O_11_ | 23.17 | 515.2465 | 515.2487 | -4.208 | 662(100), 492(49), 323(28), 986(22) | DZ | 6 |
| 495 | Eupatilin^*^ | C_18_H_16_O_7_ | 23.21 | 343.0802 | 343.0812 | -3 | 328(100), 313(1) | AY | 1 |
| 496 | 9,12,13-Trihydroxy octadecenoic acid | C_18_H_34_O_5_ | 23.33 | 329.2314 | 329.2323 | -2.735 | 229(100), 211(60), 311(47), 293(38) | AY DS | 1, 2, 3 |
| 497 | 9,10,13-trihydroxy-(E)-11-octadecenoic acid | C_18_H_34_O_5_ | 23.6 | 329.2313 | 329.2323 | -2.918 | 171(100), 293(51), 311(36) | DS | 1, 2 |
| 498 | Dictamnoside A isomer | C_21_H_36_O_9_ | 23.89 | 431.226 | 431.2276 | -3.662 | 261(100), 187(81), 125(12), 413(2) | / | 1 |
| 499 | Gomisin N | C_23_H_28_O_6_ | 24.04 | 399.182 | 399.1802 | 4.422 | 384(100), 355(17), 353(13), 342(12) | RS | 1, 2 |
| 500 | 3-​O-​β-​D-​glucopyranosyl (1 → 3)​-​α-​L-​rhamnopyranosyl (1 → 2)​-​α-​L-​arabinopyranosyl hederagenin 28-​O-​β-​D-​glucopyranosyl (1 → 6)​-​β-​D-​glucopyranosyl ester | C_59_H_98_O_27_ | 24.32 | 1237.6112 | 1237.6212 | -8.051 | 1091(100), 1075(29), 1092(28), 621(23) | XD | 10 |
| 501 | Dictamnoside A | C_21_H_36_O_9_ | 24.35 | 431.226 | 431.2276 | -3.592 | 261(100), 187(100), 125(14), 387(3) | BZ | 1 |
| 502 | Malonyl-ginsenoside Rb1 | C_57_H_94_O_26_ | 24.43 | 1193.5901 | 1193.595 | -4.079 | 1175(100), 1103(31), 1083(14) | RS | 10 |
| 503 | Aster saponin Hb isomer | C_47_H_74_O_18_ | 24.52 | 925.4751 | 925.4791 | -4.367 | 321(100), 604(74) | / | 8 |
| 504 | Ginsenoside Ro isomer | C_48_H_76_O_19_ | 24.8 | 955.4852 | 955.4897 | -4.748 | 794(100), 523(20), 614(14), 569(11) | RS | 8 |
| 505 | Koryoginsenoside R2 | C_54_H_92_O_24_ | 24.85 | 1123.5851 | 1123.5895 | -3.916 | 961(100), 1078(100) | RS | 10-13 |
| 506 | Dihydroxydehydrodiconiferyl alcohol | C_20_H_22_O_6_ | 24.9 | 357.1354 | 357.1333 | 5.923 | 237(100), 339(98), 313(20), 243(17) | DZ | 1 |
| 507 | Casticin | C_19_H_18_O_8_ | 25.04 | 373.0906 | 373.0918 | -3.173 | 358(100), 343(4), 329(3), 355(1) | AY | 1 |
| 508 | Malonyl-ginsenoside Rb2/Rc | C_56_H_92_O_25_ | 25.18 | 1163.5796 | 1163.5844 | -4.129 | 1073(100), 1132(69), 605(68), 486(53) | RS | 10 |
| 509 | Aster saponin Hb isomer | C_47_H_74_O_18_ | 25.32 | 925.476 | 925.4791 | -3.384 | 764(100), 614(51), 720(18), 702(17) | / | 7 |
| 510 | Dipsacus saponin J isomer | C_65_H_106_O_31_ | 25.36 | 1381.658 | 1381.6634 | -3.961 | 1352(100), 1057(87), 603(80), 749(67) | XD | 10 |
| 511 | Ginsenoside Ro | C_48_H_76_O_19_ | 25.43 | 955.4861 | 955.4897 | -3.795 | 909(100), 730(9), 586(6), 794(6) | RS | 7 |
| 512 | Lancemaside C | C_58_H_92_O_27_ | 25.44 | 1219.5695 | 1219.5742 | -3.906 | 650(100), 874(52), 422(52), 516(50) | DS | 9 |
| 513 | Lancemaside D | C_53_H_84_O_23_ | 25.45 | 1087.5297 | 1087.532 | -2.119 | 871(100), 918(80), 690(65), 1070(58) | DS | 5 |
| 514 | Malonyl-ginsenoside Rb1 isomer | C_57_H_94_O_26_ | 25.52 | 1193.5913 | 1193.595 | -3.057 | 941(100), 895(51), 766(28), 929(27) | / | 10 |
| 515 | Koryoginsenoside R2 isomer | C_54_H_92_O_24_ | 25.61 | 1123.5847 | 1123.5895 | -4.236 | 745(100), 1069(76), 1101(41), 1086(29) | / | 10-13 |
| 516 | 1-hydroxy-2-methylanthraquinone/ 2-methoxyanthraquinone | C_15_H_10_O_3_ | 25.73 | 237.0544 | 237.0546 | -0.804 | 237(100), 209(49), 208(9), 193(8) | BJT | 1 |
| 517 | Ginsenoside Rb2^*^ | C_53_H_90_O_22_ | 25.79 | 1077.5783 | 1077.584 | -5.336 | 946(100), 784(60), 622(15), 916(15) | RS | 10 |
| 518 | Aster saponin Hb isomer | C_47_H_74_O_18_ | 25.8 | 925.4746 | 925.4791 | -4.961 | 764(100), 614(19), 702(11), 523(7) | / | 7 |
| 519 | Asperosaponin B isomer | C_51_H_80_O_20_ | 25.81 | 1011.5119 | 1011.5159 | -3.975 | 407(100), 604(37), 851(11), 757(9) | / | 8 |
| 520 | Ginsenoside Ro isomer | C_48_H_76_O_19_ | 25.87 | 955.4862 | 955.4897 | -3.722 | 909(100), 585(17), 819(17), 937(11) | RS | 7 |
| 521 | Calycopterin | C_19_H_18_O_8_ | 25.88 | 373.0909 | 373.0918 | -2.45 | 358(100), 343(3), 359(1) | TSZ | 9 |
| 522 | Ginsenoside Rb3^*^ | C_53_H_90_O_22_ | 25.9 | 1077.5786 | 1077.584 | -5.002 | 946(100), 784(58), 622(28), 766(15) | RS | 10 |
| 523 | Aster saponin Hb | C_47_H_74_O_18_ | 25.93 | 925.4758 | 925.4791 | -3.643 | 569(100), 614(46), 794(8), 656(5) | DS | 5, 6 |
| 524 | Koryoginsenoside R2 isomer | C_54_H_92_O_24_ | 25.94 | 1123.5846 | 1123.5895 | -4.352 | 1082(100), 1063(84), 604(38), 1105(26) | / | 10, 11, 12 |
| 525 | Malonyl-ginsenoside Rb2/Rc | C_56_H_92_O_25_ | 26.01 | 1163.5795 | 1163.5844 | -4.232 | 852(100), 479(78), 1119(71), 945(46) | RS | 10 |
| 526 | 13-hydroxyl-atractylenolide Ⅱ/ 8-epiatractylenolide Ⅲ | C_15_H_20_O_3_ | 26.35 | 247.1325 | 247.1329 | -1.461 | 203(100), 205(6) | BZ | 1 |
| 527 | Lancemaside D isomer | C_53_H_84_O_23_ | 26.5 | 1087.5275 | 1087.532 | -4.133 | 573(100), 1042(90), 1015(77), 1040(47) | / | 6 |
| 528 | Ginsenoside Ro isomer | C_48_H_76_O_19_ | 26.6 | 955.4861 | 955.4897 | -3.795 | 909(100), 541(35), 632(23), 407(14) | RS | 7 |
| 529 | Lancemaside D isomer | C_53_H_84_O_23_ | 27.06 | 1087.5286 | 1087.532 | -3.131 | 845(100), 698(48), 967(36), 883(22) | / | 6 |
| 530 | Lancemaside C isomer | C_58_H_92_O_27_ | 27.11 | 1219.57 | 1219.5742 | -3.504 | 1201(100), 811(68), 632(62), 1132(60) | / | 9 |
| 531 | Malonyl-ginsenoside Rd | C_51_H_84_O_21_ | 27.77 | 1031.5377 | 1031.5421 | -4.281 | 726(100), 1001(97), 952(93), 591(85) | RS | 9 |
| 532 | Dipsacus saponin K | C_53_H_86_O_23_ | 27.79 | 1089.5415 | 1089.5476 | -5.612 | 1046(100), 901(71), 730(63), 647(46) | XD | 8 |
| 533 | Foetidissimoside A isomer | C_52_H_82_O_22_ | 27.97 | 1057.5164 | 1057.5214 | -4.766 | 1011(100), 688(20), 957(7), 733(6) | / | 5 |
| 534 | Atractylenolide III^*^ | C_15_H_20_O_3_ | 27.98 | 247.1326 | 247.1329 | -1.096 | 203(100), 229(4) | DS BZ | 1 |
| 535 | Malonyl-ginsenoside Rd isomer | C_51_H_84_O_21_ | 28.81 | 1031.5391 | 1031.5421 | -2.982 | 991(100), 988(80), 941(70), 1001(45) | / | 9 |
| 536 | Foetidissimoside A | C_52_H_82_O_22_ | 29.72 | 1057.5158 | 1057.5214 | -5.343 | 745(100), 853(80), 1011(79), 544(76) | DS | 4, 5 |
| 537 | Dipsacus saponin J | C_65_H_106_O_31_ | 29.8 | 1381.6581 | 1381.6634 | -3.874 | 1220(100), 1236(23), 1074(20), 750(16) | XD | 11 |
| 538 | Ginsenoside Rd^*^ | C_48_H_82_O_19_ | 29.94 | 961.5336 | 961.5367 | -3.21 | 916(100), 784(9), 717(2), 673(2) | RS | 8 |
| 539 | Dipsacus saponin J isomer | C_65_H_106_O_31_ | 30 | 1381.657 | 1381.6634 | -4.67 | 1220(100), 1236(56), 1074(36), 1058(21) | XD | 11 |
| 540 | Ulmoidol | C_29_H_42_O_5_ | 30.13 | 469.2932 | 469.2949 | -3.432 | 425(100), 397(42), 343(36), 371(34) | DZ | 2 |
| 541 | Ginsenoside Ro isomer | C_48_H_76_O_19_ | 30.83 | 955.4861 | 955.4897 | -3.795 | 910(100), 360(32), 472(29), 755(28) | RS | 7 |
| 542 | Ulmoidol isomer | C_29_H_42_O_5_ | 31.04 | 469.2925 | 469.2949 | -4.924 | 451(100), 405(36), 333(35), 387(28) | / | 2 |
| 543 | Ginsenoside Ro isomer | C_48_H_76_O_19_ | 31.07 | 955.4866 | 955.4897 | -3.22 | 909(100), 793(28), 911(20), 909(19) | RS | 7 |
| 544 | Ginsenoside Ra8 | C_57_H_94_O_23_ | 31.14 | 1145.6065 | 1145.6102 | -3.287 | 1923(100), 1026(83), 1489(68), 664(68) | RS | 6 |
| 545 | Aster saponin Hb isomer | C_47_H_74_O_18_ | 32.53 | 925.4745 | 925.4791 | -5.026 | 879(100), 556(4), 555(3), 764(3) | / | 5 |
| 546 | Asperosaponin B | C_51_H_80_O_20_ | 33.14 | 1011.5109 | 1011.5159 | -4.934 | 912(100), 750(17), 603(16), 586(4) | XD | 8 |
| 547 | 14-​Acetoxy-​12-​senecioyloxytetradec​a-​2,​8,​10-​trien-​4,​6-​diyn-​1-​ol | C_21_H_24_O_5_ | 33.22 | 355.1529 | 355.154 | -3.211 | 311(100), 337(1) | BZ | 1 |
| 548 | 9,10-dyhydroxy-12-octadecenoic acid | C_18_H_34_O_4_ | 33.47 | 313.2367 | 313.2373 | -2.062 | 295(100), 183(51), 277(20), 195(11) | DS | 1 |
| 549 | 2-[4-(2-hydroxy-ethoxy)-1-isobutyl-1,4,6-trimethyl-hept-2-ynyloxy]-ethanol | C_18_H_34_O_4_ | 34.08 | 313.2365 | 313.2373 | -2.541 | 201(100), 295(50), 277(40), 171(29) | BZ | 1 |
| 550 | Atractylenolide III with Atractylone-9a,2-dehydration compound | C_30_H_38_O_3_ | 34.9 | 445.2778 | 445.2737 | 9.092 | 301(100), 271(87), 319(83), 139(78) | BZ | 1 |
| 551 | Asperosaponin E | C_59_H_96_O_25_ | 34.93 | 1203.6101 | 1203.6157 | -4.64 | 1058(100), 1042(36), 734(36), 896(16) | XD | 10 |
| 552 | Akebia saponin Y | C_64_H_104_O_29_ | 35.08 | 1335.6578 | 1335.658 | -0.085 | 1190(100), 734(41), 1058(38), 1204(28) | XD | 10-13 |
| 553 | 14-senecioyloxytetradeca-2E,8Z,10Etrien-4,6-diyne-1-ol isomer | C_19_H_22_O_3_ | 35.13 | 297.151 | 297.1485 | 8.242 | 183(100), 197(7), 184(6), 297(4) | / | 1, 2 |
| 554 | Asperosaponin F isomer | C_53_H_86_O_20_ | 35.14 | 1041.561 | 1041.5629 | -1.758 | 1837(100), 996(97), 713(95), 430(46) | / | 5 |
| 555 | Asperosaponin G isomer | C_47_H_76_O_16_ | 35.17 | 895.4998 | 895.505 | -5.743 | 690(100), 734(28), 852(24), 587(11) | / | 5 |
| 556 | 6-(3-hydroxy-propionyloxy)atractylenolid III isomer | C_18_H_22_O_6_ | 35.34 | 333.1333 | 333.1333 | 0.016 | 265(100), 289(1), 315(1) | / | 1-8 |
| 557 | 14-senecioyloxytetradeca-2E,8Z,10Etrien-4,6-diyne-1-ol | C_19_H_22_O_3_ | 35.44 | 297.1509 | 297.1485 | 8.04 | 183(100), 212(5), 184(5), 297(4) | BZ | 1-8 |
| 558 | Echinocystic acid-3-O-(6’-O-methyl)-β-D-glucuronopyranoside | C_37_H_58_O_10_ | 35.83 | 661.3923 | 661.3946 | -3.469 | 485(100), 530(69), 511(63), 471(29) | DS | 2-5 |
| 559 | 14-senecioyloxytetradeca-2E,8Z,10Etrien-4,6-diyne-1-ol isomer | C_19_H_22_O_3_ | 36.25 | 297.151 | 297.1485 | 8.443 | 184(100), 183(74), 297(11), 233(11) | / | 1-8 |
| 560 | Asperosaponin F | C_53_H_86_O_20_ | 36.53 | 1041.5581 | 1041.5629 | -4.571 | 734(100), 896(65), 587(52), 456(33) | XD | 8 |
| 561 | Asperosaponin G | C_47_H_76_O_16_ | 37 | 895.502 | 895.505 | -3.297 | 734(100), 587(55), 455(26), 716(12) | XD | 5, 6 |
| 562 | Asperosaponin F isomer | C_53_H_86_O_20_ | 37.16 | 1041.5586 | 1041.5629 | -4.11 | 996(100), 997(11), 827(6), 857(6) | / | 5 |
| 563 | Atractylenolatam with 6-hydroxy-3,3a-dihydro atractylenolide III-1,6-dehydration compound | C_30_H_39_NO_4_ | 37.91 | 476.2762 | 476.2795 | -7.108 | 402(100), 153(82), 171(16), 385(6) | BZ | 5 |
| 564 | Atractylenother | C_15_H_22_O_2_ | 38.16 | 233.1532 | 233.1536 | -1.572 | 233(100), 189(23), 165(20), 217(19) | BZ | 1 |
| 565 | Selina-4(14),7(11)-dien-8-one | C_15_H_22_O_2_ | 38.17 | 233.1535 | 233.1536 | -0.499 | 233(100), 187(48), 165(43), 215(30) | BZ | 5 |
| 566 | Albigenic acid/ Echinocystic acid | C_30_H_48_O_4_ | 38.39 | 471.3453 | 471.3469 | -3.281 | 453(100), 425(57), 427(47), 409(39) | DS | 1 |
| 567 | Albigenic acid/ Echinocystic acid | C_30_H_48_O_4_ | 39.48 | 471.3453 | 471.3469 | -3.408 | 453(100), 427(8), 411(6), 409(5) | DS | 1 |
| 568 | Hederagenin | C_30_H_48_O_4_ | 39.81 | 471.3452 | 471.3469 | -3.684 | 393(100), 405(36), 453(7), 410(7) | XD | 1, 2 |
| 569 | 12-senecioyloxytetradeca-2E,8Z,10Etrien-4,6-diyne-1,14-diacetate | C_23_H_26_O_6_ | 40.19 | 397.1607 | 397.1646 | -9.706 | 329(100), 351(8), 335(3), 333(2) | BZ | 1 |
| 570 | Dibutyl terephthalate | C_16_H_22_O_4_ | 42.23 | 277.143 | 277.1434 | -1.428 | 233(100), 121(42) | DS | 1 |
| 571 | 3,4-Bis(4-hydroxy-3-methoxyphenyl)-1,6-hexanediol | C_20_H_26_O_6_ | 42.46 | 361.1644 | 361.1646 | -0.374 | 293(100) | DS | 2, 3, 5-8 |
| 572 | Stigmasetyl-b-Dglucoside sulfate | C_35_H_58_O_9_S | 42.89 | 653.3717 | 653.3718 | -0.123 | 397(100), 415(42), 235(9), 305(6) | DS | 5 |
| 573 | Corosolic acid | C_30_H_48_O_4_ | 44.62 | 471.3455 | 471.3469 | -3.026 | 423(100), 407(36), 421(31), 425(14) | DZ | 1 |
| 574 | 3,27-Dihydroxyolean-12-en-28-oic acid | C_30_H_48_O_4_ | 45.06 | 471.3451 | 471.3469 | -3.748 | 405(100), 440(71), 393(52), 411(23) | BJT | 1 |
| 575 | Stigmasterol sulfate | C_29_H_48_O_4_S | 46.85 | 491.3191 | 491.319 | 0.27 | 255(100), 253(9), 235(3), 447(1) | DS | 2 |
| 576 | 3-hydroxy-propionic acid tridecyl ester isomer | C_16_H_32_O_3_ | 48.14 | 271.2262 | 271.2268 | -2.106 | 225(100), 252(20), 226(14), 253(14) | / | 1, 2 |
| 577 | 3β,5-alkenyl-spirostol | C_27_H_42_O_3_ | 49.38 | 413.3039 | 413.305 | -2.738 | 396(100), 369(86), 372(86), 351(68) | BJT | 1 |
| 578 | Betulinic acid | C_30_H_48_O_3_ | 49.85 | 455.3501 | 455.352 | -4.045 | 397(100), 395(99), 261(99), 412(96) | DZ | 1 |
| 579 | 3-hydroxy-propionic acid tridecyl ester | C_16_H_32_O_3_ | 50.6 | 271.2262 | 271.2268 | -2.106 | 225(100), 253(4), 223(1) | BZ | 1, 2 |
| 580 | Ursolic acid | C_30_H_48_O_3_ | 50.75 | 455.3505 | 455.352 | -3.254 | 407(100), 387(10), 437(8), 448(6) | XD DZ | 1 |
| 581 | Ginsenoside Rh1 isomer | C_36_H_62_O_9_ | 54.12 | 637.429 | 637.431 | -3.122 | 592(100), 431(74), 401(56), 370(49) | RS | 2 |
| 582 | (Z,Z)-9,12-octadecadienoic acid/ 1-(2-hydroxy-2-methyl-propyl)-2,5,5,8a-tetramethyl-1,2,4a,5,6,7,8,8a-octahydronaphthalen-2-ol | C_18_H_32_O_2_ | 54.63 | 279.2313 | 279.2319 | -1.922 | 261(100), 261(6), 259(6), 243(5) | BJT BZ | 1 |
| 583 | Ginsenoside Rh1 | C_36_H_62_O_9_ | 55.09 | 637.4285 | 637.431 | -3.984 | 547(100), 548(77), 569(70), 365(34) | RS | 2 |
| 584 | 14-​Acetoxy-​12-​senecioyloxytetradec​a-​2,​8,​10-​trien-​4,​6-​diyn-​1-​ol isomer | C_21_H_24_O_5_ | 57.46 | 355.1558 | 355.154 | 4.955 | 311(100), 337(26), 293(17), 117(14) | / | 1, 2, 4-8 |
| 585 | Ginsenoside Rk3 | C_36_H_60_O_8_ | 59.32 | 619.4175 | 619.4204 | -4.787 | 552(100), 581(31), 283(26), 573(22) | RS | 2 |
| 586 | Methyl heptadecanoate | C_18_H_36_O_2_ | 60.05 | 283.2623 | 283.2632 | -2.919 | 263(100), 265(91), 283(82), 239(61) | TSZ | 19 |
| 587 | Stearic acid | C_18_H_36_O_2_ | 60.07 | 283.2627 | 283.2632 | -1.613 | 265(100) | DS | 16, 18 |
| 588 | Ginsenoside Rh4 | C_36_H_60_O_8_ | 60.25 | 619.4174 | 619.4204 | -4.981 | 573(100), 415(97), 253(72), 351(14) | RS | 2 |
| 589 | Ginsenoside Rh2 | C_36_H_62_O_8_ | 60.7 | 621.4332 | 621.4361 | -4.707 | 576(100), 417(79), 237(68), 485(20) | RS | 2 |

*: Indicates that the compound has been confirmed by the standard.
